# Superdurable, Flexible Ceramic Nanofibers for Sustainable Passive Radiative Cooling

**DOI:** 10.1021/acsnano.5c05958

**Published:** 2025-07-31

**Authors:** Dai-Chi Chen, Ching-Wen Hwang, Ching Yin Chang, Chia-Ling Kuo, Hsuen-Li Chen, Pin-Hui Lan, Meng-Ting Tsai, Tzu-Wei Wang, Dehui Wan

**Affiliations:** † Institute of Biomedical Engineering, 34881 National Tsing Hua University, Hsinchu 300044, Taiwan; ‡ Department of Materials Science and Engineering, 33561 National Taiwan University, Taipei 106319, Taiwan; § Center of Atomic Initiative for New Materials, National Taiwan University, Taipei 106319, Taiwan; ∥ Department of Materials Science and Engineering, 34881 National Tsing Hua University, Hsinchu City 300044, Taiwan; ⊥ Institute of Oral Biology, College of Dentistry, National Yang Ming Chiao Tung University, Taipei 112304, Taiwan

**Keywords:** ceramic nanofibers, passive radiative cooling, flame resistance, electrospinning, environmental aging resistance

## Abstract

Passive daytime radiative cooling can mitigate global warming but requires durable and resilient materials for real-world applications. Here, a robust superhydrophobic ZrO_2_–Al_2_O_3_ nanofiber (sh-ZANF) membrane is fabricated via electrospinning followed by fluorine-free surface modification. Optically engineered sh-ZANF attains an extremely high solar reflectivity of 97.7% due to strong scattering at numerous fiber/air interfaces with a high refractive index contrast (*n*_fiber_ = 2.04, *n*_air_ = 1). sh-ZANF also possesses a high atmospheric transparency window emissivity of 95.6% originating from phonon-polariton resonances of abundant Al–O/Zr–O bonds without a strong Reststrahlen effect. The optimal sh-ZANF membrane demonstrates subambient cooling of 6.6 °C and a maximum cooling power of 125 W/m^2^ under 817 W/m^2^ solar irradiance. Coverage by sh-ZANF cools building models, automobile models, and hand-held cameras under sunlight by 14.7 °C, 16.8 °C, and 11.1 °C, respectively. Equipping buildings with sh-ZANF is estimated to save more than 10 MJ/m^2^ annually and reduce CO_2_ emission by up to 27%. Moreover, these all-ceramic nanofibers can withstand temperatures exceeding 1400 °C, safeguarding buildings and their occupants during fire emergencies. Our sh-ZANF also displays attractive self-cleaning properties and successfully passes accelerated environmental aging tests, suggesting its applicability for future energy-efficient and sustainable cooling strategies.

## Graphical abstract

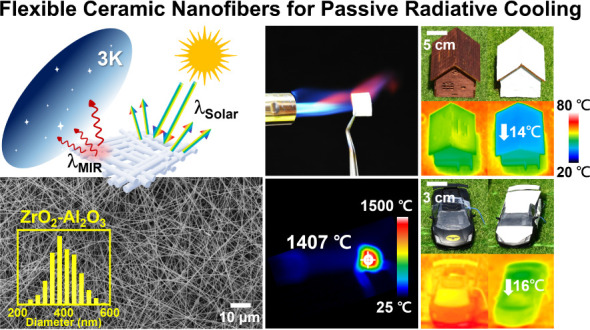

## 1 Introduction

Global warming poses numerous environmental challenges including rising sea levels, heatwaves, and wildfires.^1–3^ The frequency and severity of heatwaves have increased alarmingly, exacerbating wildfires, endangering property and human life, and hindering evacuation efforts.^1^ This impact is particularly pronounced in regions such as Hawaii, Western United States, and Europe.^1,4^ Despite significant efforts to mitigate global warming, electricity demand continues to rise sharply. In particular, the power consumption of air-conditioning systems is expected to triple by 2050,^5^ and the rapid development of artificial intelligence^6^ is further accelerating the increase in energy demand. Innovative passive cooling technologies can mitigate the energy demand fueled by climate crises.

Passive daytime radiative cooling (PDRC) systems are energy efficient,^7,8^ operating by enhancing sunlight reflectance to minimize solar heat gain, while promoting mid-infrared (MIR) emittance (particularly within the atmospheric transparent window (ATW)) to dissipate heat to outer space (∼3 K). Early photonic coolers based on inorganic materials leveraged phonon-polariton resonance to selectively maximize the ATW emittance, and were combined with a metal layer to reflect sunlight.^9^ However, their mass production is limited by the complicated and expensive nanoprecise fabrication processes. In comparison, polymer-based thermal emitters are simple, affordable, scalable, flexible, and have precisely tunable structures, suitable for fabrication as porous coatings,^10–12^ meta-structured membranes,^13,14^ particle-embedded matrices,^15–17^ dielectric paints,^18,19^ *etc*. These systems utilize their disordered nano- and microstructures to enhance sunlight reflection through strong scattering, coupled with improved ATW emissivity due to the molecular vibrations of polymer chains. However, for prolonged usage in real-world environments, these polymeric emitters must overcome several challenges including susceptibility to UV degradation,^20^ yellowing,^21^ and flammability.

For sustainable outdoor applications in extreme environments,^22^ PDRC materials must exhibit exceptional cooling performance, robust resistance to environmental aging,^23,24^ self-cleaning capabilities,^23^ and flame retardancy.^25,26^ Chen et al. immobilized SiO_2_ particles on cellulose fibers to improve thermal stability and flame inhibition.^25^ Deng et al. packed fluorosilane-coated TiO_2_ nanoparticles as UV absorbers to mitigate polymer yellowing,^23^ and the resultant superhydrophobic surface displayed desirable antisoiling properties. However, organic fluorine compounds are environmentally unfriendly,^27^ whereas TiO_2_ nanoparticles heat up under direct sunlight. Researchers have explored functionalized ceramic fillers in polymeric matrices but failed to address the inherent drawbacks of polymeric materials. A more fundamental solution is using all-ceramic materials in radiative cooling applications.

All-ceramic PDRC emitters, such as porous coatings,^28,29^ stacked particle films,^30–32^ nanofiber membranes,^33–35^ and aerogels,^36,37^ offer high solar reflectivity, low sunlight absorptivity, and strong UV resilience owing to their enhanced interfacial refractive index differences and wide bandgaps. More importantly, they have inherent thermal stability and blaze prevention properties that are crucial for residential building envelope materials.^38,39^ For instance, a hierarchically porous Al_2_O_3_ coating fabricated through phase inversion achieved extremely high solar reflectance (*R*_solar_ = 99.6%) and ATW emittance (ε_ATW_ = 96.5%), coupled with a high temperature resistance of ∼1000 °C.^29^ Notably, its *R*_solar_ only declined by 1.3% after one-year outdoor exposure. Hu et al. reported a solution-processable glass/Al_2_O_3_ coating with *R*_solar_ = 96%, ε_ATW_ = 95%, and a subambient cooling of 3.5 °C under a solar intensity (*I*_solar_) of 790 W/m^2^. When used with an external glass protective layer, this coating experienced only 3% reduction in *R*_solar_ after a 3-year-equivalent antisoiling experiment.^28^ However, both ceramic coatings are brittle and inflexible, limiting their practical applications.^40,41^ Tsai et al. developed a flexible PDRC membrane composed of electrospun SiO_2_ nanofibers with an average diameter of 420 ± 84 nm via optical engineering.^33^ The cooling membranes demonstrated a high *R*_solar_ of 97% and an ε_ATW_ of 90%, together with exceptional weather adaptability and flame resistance (∼1200 °C), suggesting durability in outdoor environments. However, the MIR emittance of SiO_2_-based emitters is low because of their strong Reststrahlen reflection in the ATW.^42^ Similarly, Xin et al. reported that while electrospun Al_2_O_3_ nanofiber membranes achieved a high *R*_solar_ of 96%, their ε_ATW_ remained constrained to 87%.^35^ Also, Li et al. developed a stacked Al_2_O_3_/SiO_2_ nanofibrous aerogel demonstrating a slightly improved ε_ATW_ of 93% although still limited by the Reststrahlen effect,^36^ and excellent temperature resistance from −190 to 1100 °C. Nevertheless, actual temperatures in various fire scenarios can exceed 1300 °C,^38,39^ starkly highlighting the inadequacy of recently reported ceramic PDRC materials that are typically composed of SiO_2_ and/or Al_2_O_3_. Therefore, it remains challenging to develop PDRC materials with excellent cooling performance, long-term durability and resilience in extreme environments.

In this study, a membrane of superhydrophobic ZrO_2_–Al_2_O_3_ nanofiber (sh-ZANF) was designed as a durable PDRC material for sustainable real-world applications. ZrO_2_ can overcome the aforementioned limitations, given its high refractive index in the solar band (*n* = 2.07), wide bandgap (5.7 eV), and exceptionally high melting point (2370 °C), providing superior sunlight scattering and flame resistance compared to Al_2_O_3_, SiO_2_, and TiO_2_. However, ZrO_2_ nanofibers are brittle and lack efficient ATW emittance. Incorporating a small amount of Al_2_O_3_ can enhance the fiber flexibility and improve the MIR optical properties. First, systematic optical calculations were performed using Mie theory and the two-dimensional finite-difference time-domain (2D-FDTD) method. Compared to the SiO_2_ and Al_2_O_3_ counterparts, ZANF exhibits superior solar reflectivity owing to its significantly enhanced scattering efficiency, as well as higher MIR emissivity, especially in ATW regions, by mitigating the Reststrahlen effect.^43^ Experimentally, the optimal sh-ZANF membrane demonstrated excellent flexibility, ultralight weight, and exceptional optical properties, as well as outstanding fire resistance to provide protection to covered architectural structures during fire emergencies. Finally, anticorrosion, antisoiling, and anti-UV tests and accelerated environmental aging assessments were conducted to evaluate the durability of sh-ZANF under diverse and challenging open-air conditions. Hence, sh-ZANF demonstrates long-term viability as a carbon-neutral PDRC material, particularly for weight-sensitive applications and irregular surfaces such as building envelopes, automobile coverings, and wearable electronics shielding.

## 2 Results and Discussion

### 2.1 Design of sh-ZANF for Sustainable Passive Radiative Cooling

Figure 1A highlights the characteristics of sh-ZANF for real-world applications, including scalability, ultralight weight, blaze prevention, and weather resistance. The all-ceramic nanofibrous membranes were fabricated via a sol–gel/electrospinning process employing Zr­(CH_3_COOH)_4_, AlCl_3_·6H_2_O, and Al­(O-*i*Pr)_3_ as precursors and poly­(ethylene oxide) (PEO) as a cospinning agent, followed by calcination at 800 °C (Figures S1 and 1B). Superhydrophobicity was achieved through a fluorine-free surface modification process with dimethoxydimethylsilane (DMDMS) and tetraethoxysilane (TEOS).^44^ Figure 1C shows the highly porous nanofibrous morphology, featuring an average fiber diameter of 404 ± 58 nm (200–600 nm) and a porosity of 93%. In Figure 1D, scanning transmission electron microscopy (STEM) images with energy dispersive spectrometry (EDS) elemental mapping confirmed the presence of Zr, Al, O, and Si in a single representative sh-ZANF. Quantitative analysis using inductively coupled plasma-mass spectrometry (ICP-MS) revealed a composition of 91 wt % ZrO_2_ and 9 wt % Al_2_O_3_, which is consistent with the precursor composition (Figure S2). Note that sh-ZANF provides extremely strong sunlight scattering owing to the increased refractive index contrast at its numerous fiber/air interfaces. Meanwhile, the abundant Zr–O and Al–O covalent bonds can induce phonon-polariton resonances,^45^ collectively contributing to the desired MIR emittance for effective heat radiation. Consequently, the sh-ZANF membrane has significant potential for PDRC.

**Figure 1:**
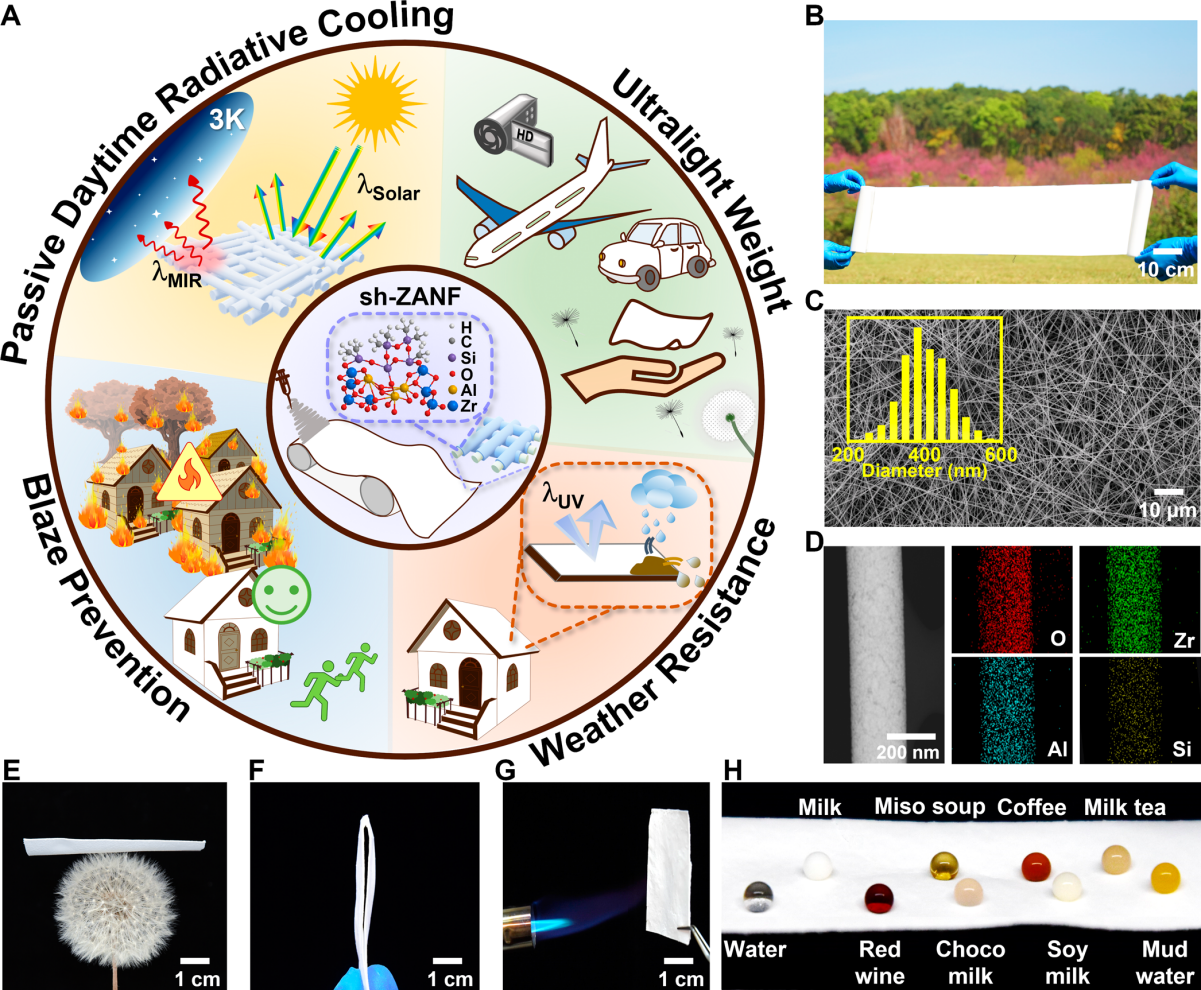
(A) Schematic representation of sh-ZANF with remarkable properties (passive daytime radiative cooling, ultralight weight, weather resistance, and blaze prevention) fabricated through a scalable electrospinning technique followed by fluorine-free hydrophobic surface modification. (B) Photograph of a large-scale sh-ZANF membrane. (C) SEM image of homogeneously distributed sh-ZANFs with an average diameter of 404 ± 58 nm. (D) STEM image with EDS mapping of a representative single sh-ZANF. (E–G) Photographs highlighting the (E) lightweight (denisty ∼ 0.163 g/cm^3^), (F) flexibility, and (G) blaze prevention of sh-ZANF. (H) Hydrophobicity of sh-ZANF when exposed to different liquids: water, milk, red wine, miso soup, choco milk, coffee, soy milk, milk tea, and mud water.

The sh-ZANF nanofibrous membrane is ultralight (Figure 1E) and remarkably flexible (Figure 1F), enabling expanded cooling applications on various surfaces such as buildings, aircraft, vehicles, wearables, and hand-held electronics.^46^ More importantly, sh-ZANF is nonflammable when placed directly under butane fire (Figure 1G), attributed to its inherent fireproof ceramic components of ZrO_2_ and Al_2_O_3_. Therefore, it offers strong resistance to ultrahigh temperatures, which is crucial for building safety, particularly in the context of wildfire disasters. Furthermore, sh-ZANF can repel many types of aqueous droplets (Figure 1H), suggesting a self-cleaning ability against mud slurry or acid rain. Notably, the fluorine-free hydrophobic coating step is more eco-friendly than traditional fluorine-based methods.^7,20,21,23,27,29,33,44^ Finally, the large bandgaps of ZrO_2_ and Al_2_O_3_ provide sh-ZANF with excellent UV resistance under direct sunlight exposure. Overall, we expect sh-ZANF to function as a sustainable, ultralightweight, flame-retardant, and weather-resistant radiator in exterior architectural PDRC materials.

### 2.2 Theoretical Investigation of Optical Properties of sh-ZANF

To systematically elucidate their optical behavior, three types of ceramic single fibers (ZANF, Al_2_O_3_ and SiO_2_) and their stacked fibrous membranes were theoretically investigated using Mie theory and 2D-FDTD calculations, respectively. First, the effective complex refractive index (*N* = *n* + *ik*) of ZANF was obtained from the Maxwell Garnett mixing rule (Figure S3).^47^ Note that the *n* value of ZANF is approximately 2.04 in the solar band, significantly exceeding those of Al_2_O_3_ (1.72–1.81) and SiO_2_ (1.42–1.48) nanofibers, as shown in Figure S3A. Figure 2A reveals the Mie scattering efficiency across the solar band (0.3–2.5 μm) of a single ceramic nanofiber at the diameters of 100–2000 nm. ZANF demonstrated a substantial increase in scattering efficiency when the fiber diameter ranged from 200 to 1500 nm. This behavior closely resembled that of ZrO_2_ nanofibers, as the high ZrO_2_ content (91 wt %) in ZANF results in optical properties predominantly governed by the intrinsic *n* and *k* values of ZrO_2_ (Figure S3). More importantly, ZANF exhibited superior sunlight scattering capabilities compared to Al_2_O_3_ and SiO_2_ nanofibers, particularly in the near-infrared (NIR) band. Additionally, the MIR absorption spectra of ZANF highlighted its potential as an effective broadband emitter (Figure S4).

**Figure 2:**
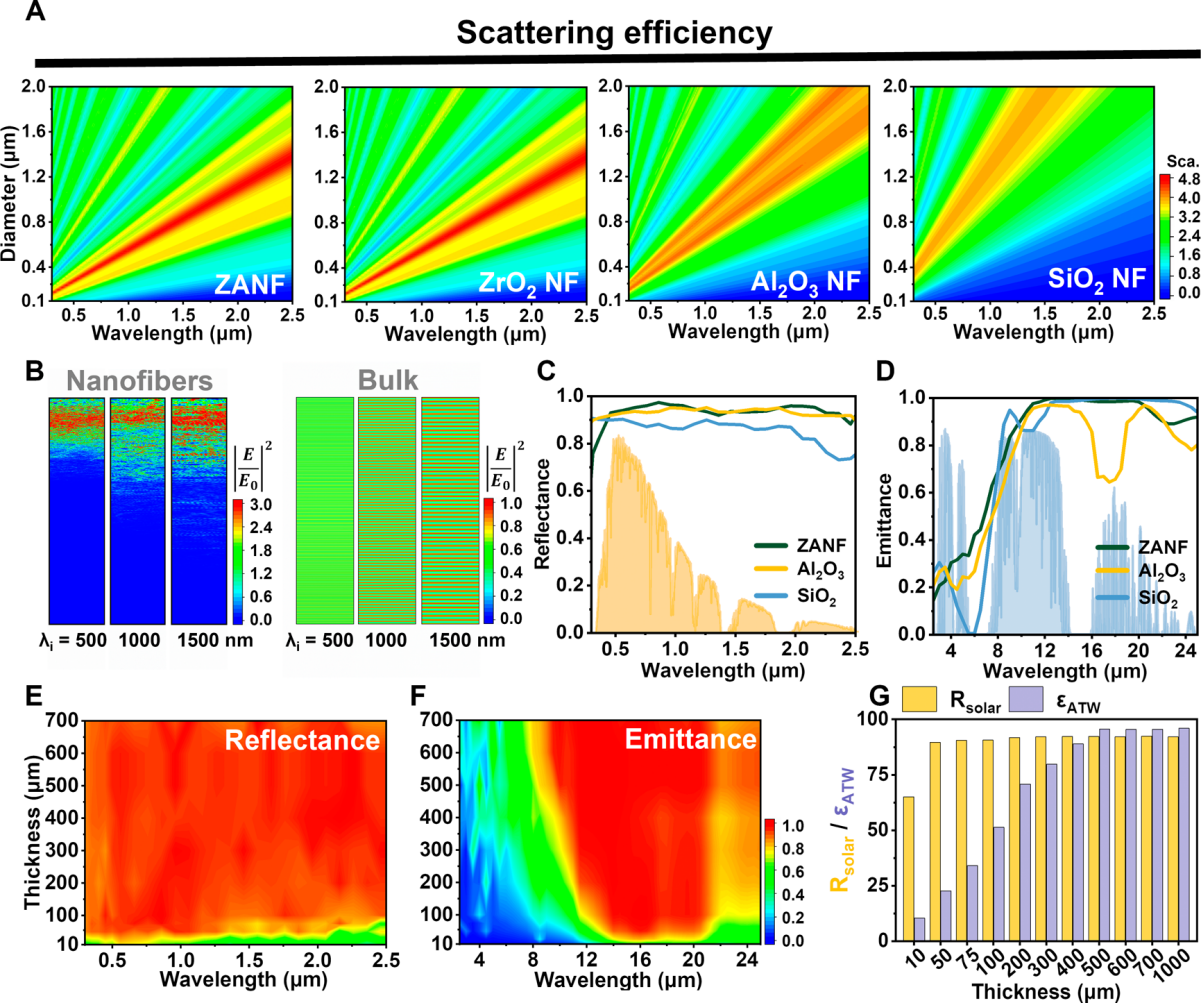
(A) Calculated scattering efficiencies at solar wavelengths (0.3–2.5 μm) for ZANF, ZrO_2_ nanofibers, Al_2_O_3_ nanofibers, and SiO_2_ nanofibers with diameters varying from 100 to 2000 nm. (B) Cross-sectional views of electric field distribution of a ZANF membrane (thickness = 300 μm, width = 8 μm) and the bulk counterpart (thickness = 10 μm, width = 8 μm) in 2D-FDTD simulations. Incident light wavelength (λ_i_): 500, 1000, and 1500 nm. (C,D) Calculated (C) solar reflectance and (D) MIR emittance spectra of ZANF, Al_2_O_3_, and SiO_2_ nanofibrous membranes with fixed thickness of 500 μm and porosity of 90%. (E,F) Calculated (E) solar reflectance and (F) MIR emittance spectra of ZANF membranes with thicknesses of 10–700 μm. (G) Comparison of calculated R_solar_ and ε_ATW_ values of ZANF of various thicknesses.

While optical simulations of single nanofibers can provide useful insights, practical applications involve densely stacked and interwoven nanofiber networks forming bulk membranes, which require more comprehensive modeling approaches. 2D-FDTD optical simulations were carried out for the theoretical analysis of stacked nanofibers by employing a randomly close-packed circle model (Figure S5). Figure 2B illustrates the electric field intensity profiles at three representative incident solar wavelengths (500, 1000, and 1500 nm), revealing significant light-matter interactions and strong backscattering with the piled nanostructures, in contrast to the unimpeded propagation through the bulk counterpart. Figure 2C,D display the calculated solar reflectance and MIR emittance spectra of ceramic nanofiber membranes at a thickness of 500 μm, respectively. As expected, the ZANF membrane possessed desirable reflectance values across the solar region, outperforming Al_2_O_3_ and SiO_2_ membranes (Figure 2C). According to Figure 2D, although the Al_2_O_3_ and SiO_2_ nanostructures demonstrated high emittance values at wavelengths greater than 8 μm, they suffered from significant emittance dips within the atmospheric transparent windows of 16–18 and 8–12 μm due to intrinsic Reststrahlen reflection^43^ (Figure S7), thus restricting their heat radiation capacity. In contrast, ZANF achieved exceptionally high emittance values across a wide MIR region of 8–25 μm, which was attributed to decreasing *n* values, moderate *k* values, and absence of Reststrahlen effect in the atmospheric transparent windows (Figures S3 and S7). Further discussion of the Reststrahlen effect can be found in the Supporting Information.

Furthermore, Figure 2E,F depicts the theoretical spectroscopic behaviors of ZANF membranes with increasing thicknesses from 10 to 700 μm, and the corresponding solar reflectance (*R*_solar_) and ATW emittance (ε_ATW_) values were calculated (Figure 2G). The reflectance across the solar band rapidly grew with membrane thickness and achieved saturation at 500 μm with *R*_solar_ = 0.923 (for comparison, this value was 0.917 for Al_2_O_3_ and 0.854 for SiO_2_ membranes at the same thickness). Meanwhile, the MIR emittance sharply increased with thickness, especially within the ATW region, plateauing at 500 μm with ε_ATW_ = 0.957 (0.909 for Al_2_O_3_ and 0.931 for SiO_2_). These optical simulation results clearly indicate that our ZANF has outstanding PDRC potential superior to that of commonly used ceramic emitters (i.e., Al_2_O_3_ and/or SiO_2_).

### 2.3 Microstructure and Spectroscopic Properties of sh-ZANF for Optimal PDRC Performance

First, ZrO_2_–Al_2_O_3_ nanofibers with varying Al_2_O_3_ contents (0 to 11.5 wt %) were fabricated with a fixed membrane thickness of 50 μm. The corresponding solar and MIR spectra are presented in Figure S10. As the Al_2_O_3_ content increased, the solar reflectance gradually decreased, whereas the MIR emittance increased significantly, particularly in the ATW region. The optimal Al_2_O_3_ content for sh-ZANF was determined to be 9 wt % after balancing the optical cooling performance. Notably, while both nanofibers exhibited similar solar reflectance (*R*_solar_ ≈ 91–93%), sh-ZANF demonstrated significantly higher emittance than ZrO_2_ (ε_ATW_ = 69.2% vs 44%), suggesting improved cooling capability. Then, the microscale structure of a representative single sh-ZANF was investigated using high-resolution transmission electron microscopy (HR-TEM), as shown in Figure 3A. Randomly arranged nanocrystals were observed in high-magnification images [Figure 3A (ii),(iii)]. The crystalline regions were connected by an amorphous region [indicated by white dashed circles in Figure 3A (ii)], which played a crucial role in the exceptional flexibility. In Figure 3A­(iii), the (101) and (200) crystallite planes were identified with lattice fringe distances of ∼0.296 and ∼0.255 nm, respectively, indicating tetragonal phase zirconia (t-ZrO_2_) in the nanocrystals.^48,49^ X-ray diffraction (XRD) and Raman spectroscopy were used to confirm the full crystalline structure. Figure 3B shows the characteristic diffraction peaks of t-ZrO_2_ at 2θ values of 30.2° (101), 35.1° (110), 50.4° (200), and 60.1° (211) (JCPDS No. 80-2155) for sh-ZANF. Moreover, the Raman spectrum exhibited distinct vibration bands at 270, 316, 460, and 645 cm^–1^, indicating a full t-ZrO_2_ phase^50^ (Figure 3C). Furthermore, deconvolution of the X-ray photoelectron spectroscopy (XPS) data confirmed the existence of Zr–O, Al–O, and Zr–O–Al bonds (Figure S11).^51,52^ These observations clearly indicate the coexistence of t-ZrO_2_ and amorphous Al_2_O_3_ domains within sh-ZANF. In contrast, the HR-TEM, XRD, Raman, and XPS analyses collectively revealed that pure ZrO_2_ nanofibers consist of a monoclinic (m-ZrO_2_) phase^53,54^ with a lower symmetry in the structure (Figures S12 and 3B,C). The pure ZrO_2_ nanofibers also demonstrated extreme brittleness (Figure 3D), which is consistent with previous studies^55,56^ and strongly restricts their application. On the other hand, hybridization of Al_2_O_3_ in ZrO_2_ nanostructures provided sh-ZANF with more stable crystallites^57^ and mitigated the brittleness arising from the distorted *m*-ZrO_2_ lattice,^58^ resulting in substantial improvements in fiber flexibility. Therefore, the tensile stress–strain curve of the flexible sh-ZANF film showed linear elastic behavior in the early stages, with a Young’s modulus of 120 MPa and a mechanical strength of 2.48 MPa (Figure 3D), which are comparable to those of robust ceramic nanofibers.^48,56,59,60^

**Figure 3:**
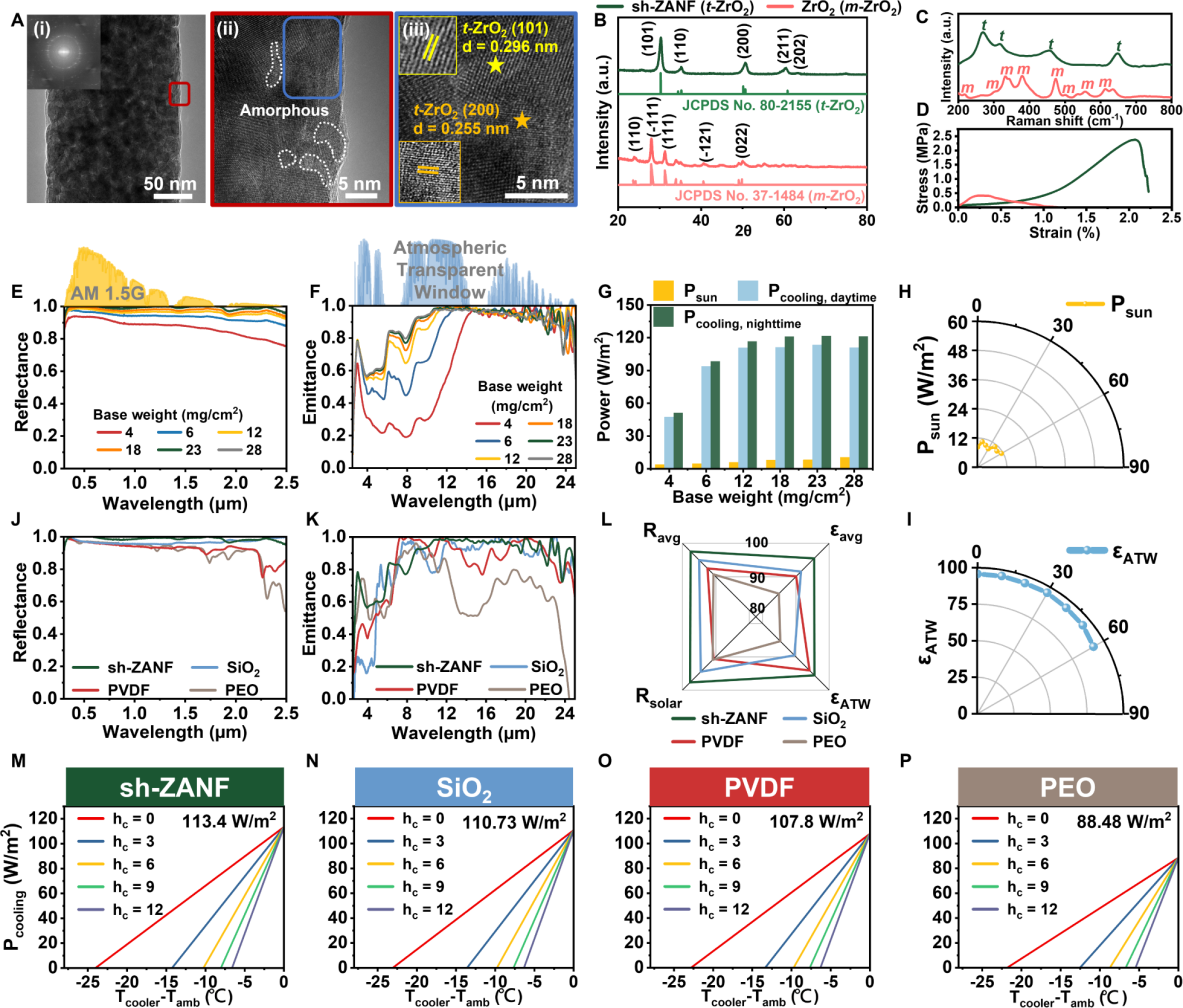
(A) HR-TEM images of sh-ZANF. (i) Randomly arranged crystalline structures. (ii) Magnified view of the region outlined in red in (i). Areas outlined by white dashes are the amorphous regions. (iii) Magnified view of the region outlined in blue in (ii), where the t-ZrO_2_ crystallite planes of (101) and (200) are distinguished by their lattice fringes. (B) XRD patterns, (C) Raman spectra, and (D) tensile stress–strain curves of sh-ZANF and ZrO_2_ nanofiber membranes. (E) Solar reflectance and (F) MIR emittance spectra of sh-ZANF with various base weights. (G) Values of P_sun_, P_cooling, daytime_, and P_cooling, nighttime_ of sh-ZANF with various base weights. (H) P_sun_ and (I) ε_ATW_ values of the optimal sh-ZANF at various incident angles. (J) Solar reflectance and (K) MIR emittance spectra of sh-ZANF, SiO_2_, PVDF, and PEO nanofibrous membranes. (L) Comparison of *R*_avg_, ε_avg_, *R*_solar_, and ε_ATW_ for different nanofiber membranes calculated from (J,K). (M–P) Calculated equilibrium temperature and P_cooling_ of (M) sh-ZANF, (N) SiO_2_, (O) PVDF, and (P) PEO nanofiber membranes with various h_c_ values (0–12 W/m^2^ K) at *T*_a__mb_ = 303 K during the daytime.

Figure 3E,F illustrates the measured spectral reflectance and emittance of sh-ZANF with base weights varying from 4 to 28 mg/cm^2^ at solar and MIR wavelengths, respectively. There were significant increases in solar reflectance and MIR emittance upon increasing the base weight, and saturation was reached above 23 mg/cm^2^ (membrane thickness = 475 μm, *R*_solar_ = 97.7% and ε_ATW_ = 95.6%). Notably, the MIR emittance, particularly that within 8–11 μm, improved drastically as the base weight increased from 4 to 18 mg/cm^2^, primarily due to the reduction in transmittance, eventually presenting a high emittance profile without notable Reststrahlen effect (Figures S13C,D and 3F). The experimentally observed spectral trends were similar to those predicted by optical simulations (Figure 2E,F).

To further evaluate the cooling capability of sh-ZANF, *P*_cooling_ during the daytime and nighttime was calculated using the following formula:

1 $$P_{\mathrm{cooling}}\left(T\right)=P_{\mathrm{rad}}\left(T\right)-P_{\mathrm{sun}}-P_{\mathrm{atm}}\left(T_{\mathrm{amb}}\right)-P_{\mathrm{cond}+\mathrm{conv}}\left(T,T_{\mathrm{amb}}\right)$$

where *T* and *T*_amb_ are the surface temperatures of the PDRC material and ambient air, respectively. *P*_cooling_(*T*) is the theoretical net cooling power; *P*_rad_(*T*) is the radiation power of the emitter at temperature *T*; and *P*_sun_ and *P*_atm_(*T*_amb_) are the solar absorption power and atmospheric radiation power at *T*_amb_, respectively. *P*_cond+conv_ *(T, T*_amb_) is the nonradiative power loss caused by heat conduction and convection. As depicted in Figure 3G, *P*_cooling_ in both daytime and nighttime demonstrated substantial growth with increasing base weight, accompanied by a trivial increase in *P*_sun_. Importantly, sh-ZANF with an optimal base weight of 23 mg/cm^2^ achieved exceptional *P*_cooling_ values at both nighttime (121.6 W/m^2^) and daytime (113.4 W/m^2^) with only a *P_sun_* value of 8.2 W/m^2^. In addition, it maintained *P*_sun_ values below 12 W/m^2^ and high emittance (e.g., ε_ATW_ ∼ 0.92 at 60°) for various incident angles, suggesting weak angular dependence of its spectral behaviors in both solar and MIR regions (Figure 3H,I).

Furthermore, we compared the solar reflectance and MIR emittance spectra of sh-ZANF with those of other high-performance ceramic and polymeric nanofibrous membranes of similar thicknesses, including SiO_2_, polyvinylidene difluoride (PVDF), and PEO. The results are depicted in Figures 3J,K and S14. As shown in Figure 3J, sh-ZANF exhibited significantly superior reflectance across the entire solar band, whereas that of other materials was restricted by insufficient scattering due to low *n* values and inevitable NIR absorption by organic functional groups, especially for PVDF and PEO nanofibers. In addition, sh-ZANF displayed desirable high and broadband MIR emittance, particularly compared with that of SiO_2_ nanofibers (Figure 3K). Therefore, among all samples, our sh-ZANF possessed the highest *R*_solar_, average solar band reflectance (*R*_avg_), ε_ATW_, and average MIR emittance (ε_avg_) at 97.7%, 97.6%, 95.6%, and 95.5% (Figure 3L). These optical features highlight the advantages of sh-ZANF over other nanofibrous emitters for effective passive radiative cooling, as shown in Figure 3M–P. The daytime *P*_cooling_ value of sh-ZANF (113.4 W/m^2^) was not only higher than that of SiO_2_ (110.7 W/m^2^), PVDF (107.8 W/m^2^), and PEO (88.5 W/m^2^) fibrous membranes, but also superior to those of previous state-of-art PDRC emitters.^23,27,28,33,34,36,42,61–66^ In addition, the maximum subambient temperature difference (*T*_cooler_ *– T*_amb_) was compared for samples at various nonradiative heat transfer coefficients (*h*_c_ = 0, 3, 6, 9, and 12 W/m^2^ K). The estimated *T*_cooler_ *– T*_amb_ of sh-ZANF progressively declined from 24.0, 14.3, 10.3, 8.0, to 6.6 °C as *h*_c_ increased (0–12 W/m^2^ K), but the values remain higher compared to other samples. Furthermore, five additional batches of sh-ZANF samples were fabricated, achieving an average *R*_solar_ of 98.4 ± 0.7%, ε_ATW_ of 94.9 ± 0.5%, *P*_cooling_ of 109.3 ± 1.9 W/m^2^, and *P*_sun_ of 10.7 ± 1.9 W/m^2^, thereby confirming excellent reproducibility across batches (Figure S15). The above results strongly imply that among ceramic and polymeric coolers, sh-ZANF has excellent potential as large-scale, flexible, and superior PDRC radiators.

### 2.4 Ultrahigh Temperature Resistance of sh-ZANF for Architectural Applications

Thermal stability and high-temperature resistance are critical for PDRC materials, particularly for applications in buildings and aerospace.^46^ However, most reported PDRC materials fail to meet these stringent requirements. As depicted in Figure 4A, the sh-ZANF membrane (thickness ∼ 400 μm) withstood thermal shock exceeding 1400 °C from a butane torch without deformation or cracking. It also exhibited excellent insulation under thermal stress (Figure 4B). In Figure 4C, sh-ZANF demonstrates remarkable fire resistance: when directly exposed to a blazing heat source (∼1400 °C) for 1200 s, it showed neither ignition nor any discernible deterioration. In contrast, the SiO_2_ nanofibrous membrane deformed significantly after 400 s, and the PEO nanofibers ignited within only 0.5 s. These results demonstrated the excellent thermal stability and fire retardancy of sh-ZANF, which could extend the escape time and hinder fire propagation during emergencies. For instance, Figure 4D,E demonstrates that a bare stainless-steel sheet (a common structural material in buildings) experienced a phase transition from austenite to martensite owing to heat exposure and sudden cooling,^67^ causing reductions in flexural strength and modulus by 26% and 28%, respectively (Figure 4E). Coverage with sh-ZANF efficiently protected this sheet from surface oxidization and structural phase transitions under direct blaze exposure. The excellent flame-protection ability of sh-ZANF for aluminum sheets is also clearly shown in Figure S18. Further, sh-ZANF prevented the deformation and collapse of a stainless-steel house model when it was used as an exterior architectural material (red arrows in Figure 4F). A fire resistance test was conducted according to the ISO 834 specification^68^ (Figure S19A). As shown in Figures 4G and S19B,C, changes in the spectra and appearance of samples (i.e., *R*_solar_ from 97.7% to 96.6%, and ε_ATW_ from 95.6% to 95.1%) were negligible, indicating the excellent potential of sh-ZANF as a fireproofing material in actual applications.

**Figure 4:**
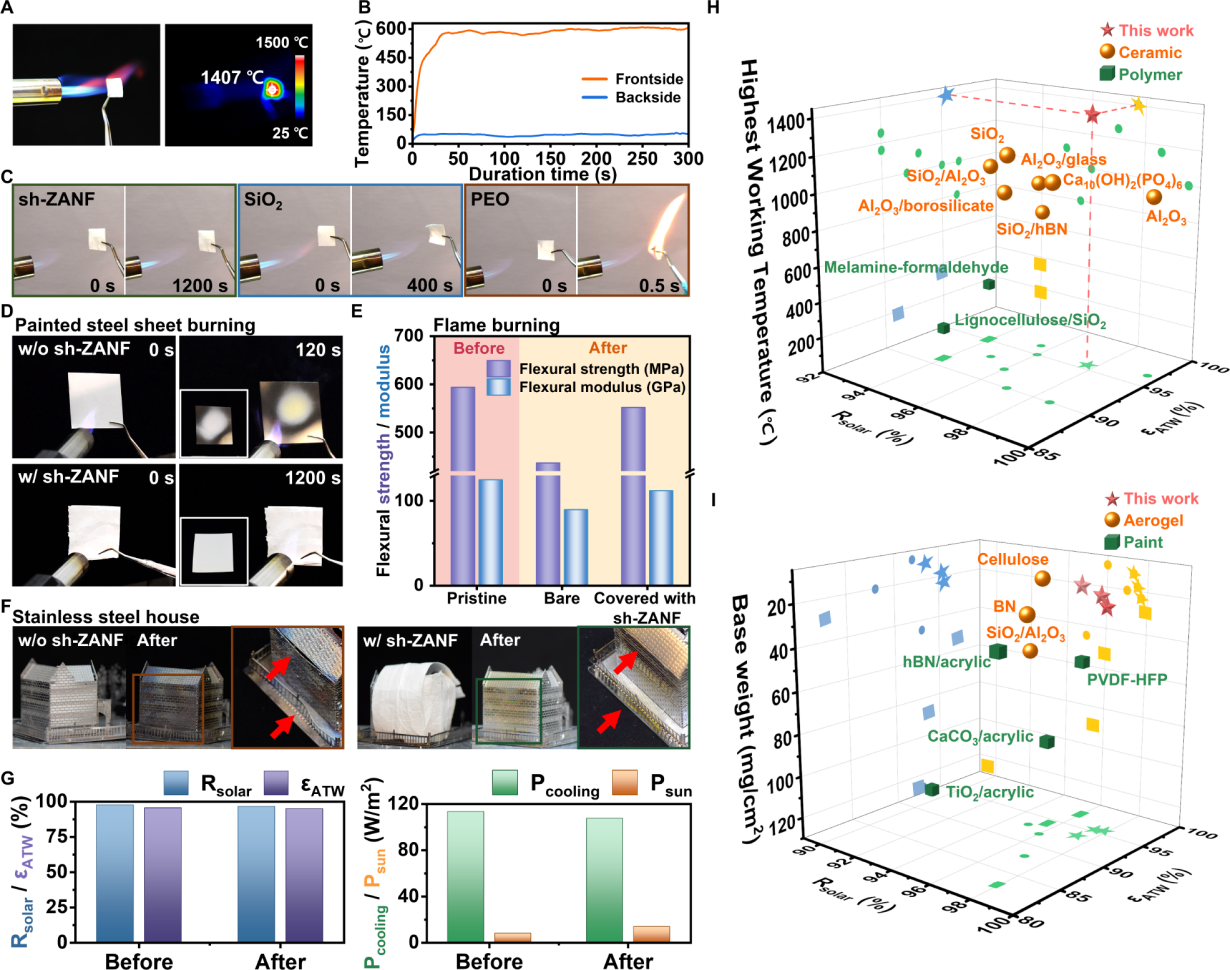
(A) Photograph and thermal infrared image of sh-ZANF exposed to blowtorch flame. (B) Illustration of the remarkable thermal insulation of sh-ZANF. (C) Photographs of sh-ZANF, SiO_2_, and PEO nanofiber membranes during the combustion test. (D) Photographs of painted steel sheets with and without coverage by sh-ZANF during exposure to blowtorch flame. Insets: photographs of the steel sheets after flame exposure. (E) Flexural strength and modulus of pristine, bare, and sh-ZANF-covered steel sheets before and after flame exposure. (F) Photographs of stainless-steel house models without/with sh-ZANF coverage before and after the combustion test. (G) Values of R_solar_, ε_ATW_, P_cooling_, and P_sun_ of sh-ZANF before and after the combustion test. (H) Comparison of working temperatures and optical features (R_solar_ and ε_ATW_) determined in this study and those reported recently for high-performance ceramic/polymeric PDRC materials. (I) Comparison of base weights and optical features (R_solar_ and ε_ATW_) determined in this study and those reported recently for lightweight PDRC materials.

Figure 4H compares the working temperatures and PDRC characteristics of various high-performance ceramic/polymeric PDRC radiators.^25,26,28–30,33,34,36,42^ All-ceramic coolers tended to have better PDRC performance and operating temperatures exceeding 1000 °C. Among them, our sh-ZANF membranes not only displayed exceptionally high *R*_solar_ and ε_ATW_ values, but also set a record operating temperature of 1407 °C, significantly surpassing previously reported PDRC emitters and meeting the flame protection for buildings in various fire scenarios (>1300 °C).^36,38,39^ In addition, the lightweight sh-ZANF has distinct advantages for weight-sensitive applications such as wearables, hand-held electronics, automobiles, and aerospace vehicles. Figure 4I compares the base weights and radiative cooling capacities of lightweight PDRC materials including aerogels,^36,69,70^ paints,^10,46^ and sh-ZANF. The base weight of sh-ZANF membranes was lower than or comparable to that of reported aerogels and paints, while displaying superior optical cooling performance. Therefore, among high-performance, lightweight PDRC coolers, sh-ZANF exhibits ultrahigh-temperature resistance, extremely low weight, and exceptional radiative cooling capacity. This combination implies unparalleled potential for diverse applications, especially in buildings, automobiles, and hand-held electronics.

### 2.5 Outdoor Cooling Performance of sh-ZANF

To verify the cooling performance of sh-ZANF during both daytime and nighttime, self-made equipment was used for outdoor field testing in Hsinchu, Taiwan (120.99°E, 24.79°N) (Figure 5A). Figure 5B illustrates the experimental setup, which used foams and polyethylene (PE) film to minimize heat transfer by conduction and convection while maintaining MIR transparency. To monitor the net cooling power, a polyimide (PI) heater was used to maintain the temperature of sh-ZANF (*T*_sh‑ZANF_) at the ambient temperature (*T*_amb_) inside the foam box, as recorded by a shaded thermocouple. Figure 5C–F display the real-time subambient temperature and measured cooling power profiles of sh-ZANF membranes during the day and night, respectively. With a maximum daytime *P*_cooling_ of 125 W/m^2^ (*I*_solar_ = 817 W/m^2^) and an average value of 110 W/m^2^, *T*_sh‑ZANF_ dropped by an average of 4.6 °C and a maximum of 6.6 °C (Figure 5C,E). At nighttime, *P*_cooling_ reached as high as 112 W/m^2^ with an average of 90 W/m^2^, resulting in a peak temperature drop of 4.6 °C (Figure 5D,F). The high *P*_cooling_ values in both daytime and nighttime agreed with the theoretically calculated values. The relatively lower *P*_cooling_ values at night might be attributed to the lower *T*_amb_ and higher relative humidity^71^ (Figure S20).

**Figure 5:**
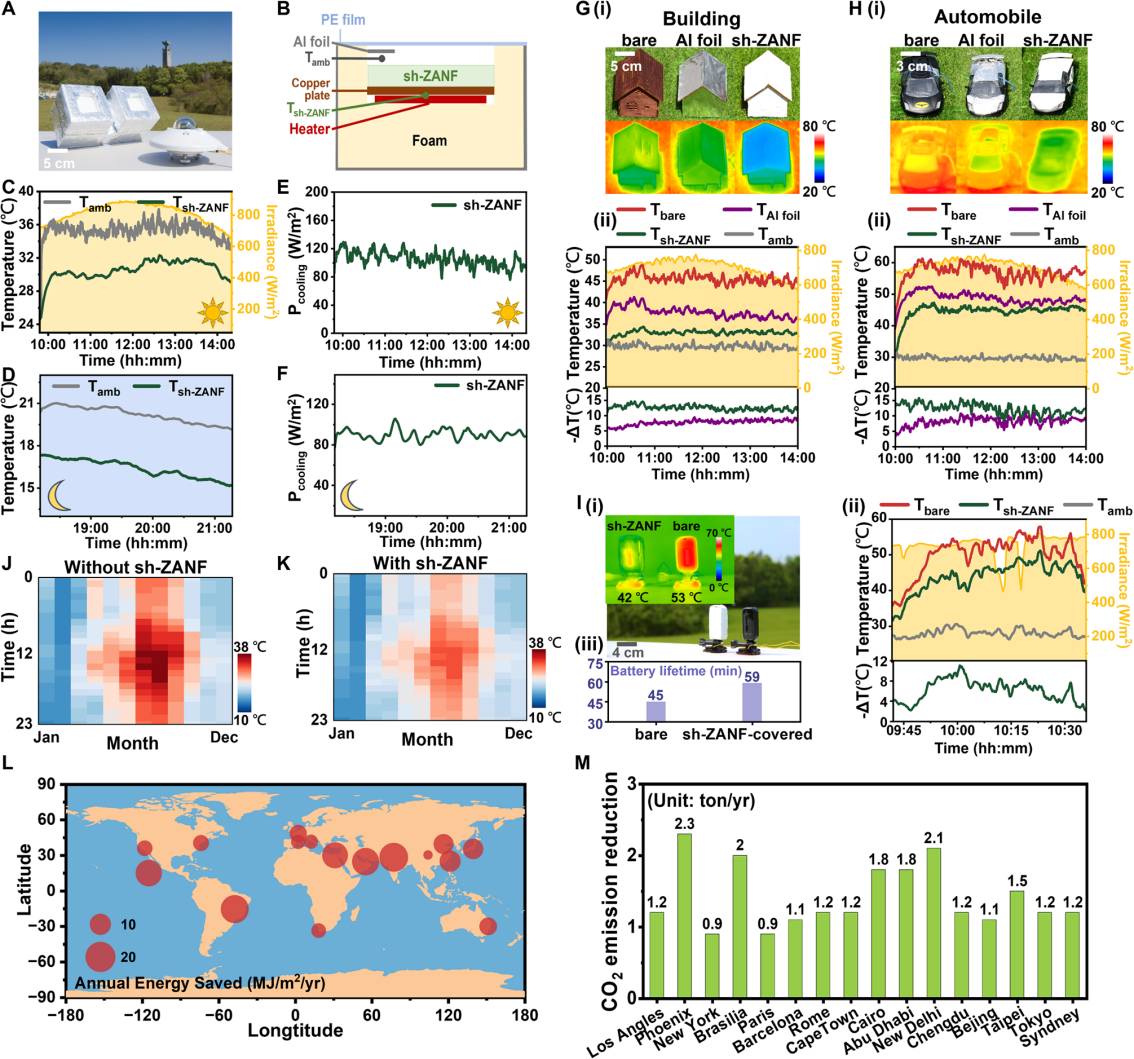
(A) Photograph and (B) schematic representation of the apparatus used for the thermal measurements. (C) Daytime and (D) nighttime temperature profiles of the subambient cooling performance of sh-ZANF, measured in Hsinchu, Taiwan (120.98°E, 24.78°N). (E,F) P_cooling_ values of sh-ZANF measured during (E) daytime and (F) nighttime. (G,H) (i) Photographs and thermal images of the (G) building and (H) automobile models covered with nothing (bare), Al foil, and sh-ZANF. (ii) Interior temperature changes in the (G) building and (H) automobile models and corresponding temperature decreases (−Δ*T* = *T*_bare_ – *T*_cooling material_) of two cooling materials. (I) (i) Photographs and thermal images of hand-held cameras covered with nothing and sh-ZANF. (ii) Surface temperature changes of the cameras and corresponding temperature decreases due to coverage by sh-ZANF. (iii) Battery lifetimes of the cameras covered with nothing and sh-ZANF. (J) Ambient temperatures and (K) calculated surface temperatures of sh-ZANF over 24 h and 12 months for Taipei. (L) Calculated annual energy saving and (M) CO_2_ emission reduction of sh-ZANF in 16 major cities around the world.

Next, we evaluated the practical outdoor cooling performance by continuously recording the interior temperatures and thermal infrared images of building/automobile models covered with nothing, aluminum foil, or sh-ZANF under solar irradiance (Figures 5G,H and S21). As shown in Figure 5G­(ii),H­(ii), the sh-ZANF membrane demonstrated exceptional PDRC capability for both models as compared with the bare one, and the maximum temperature drop was 14.7 and 16.8 °C for the building and automobile models, respectively, surpassing those of the Al foil-covered models. Also, the real-world feasibility of sh-ZANF was validated through tests on a wooden cabin and common construction materials, such as concrete blocks and tiles, where it demonstrated the ability to achieve significantly lower temperatures under sunlight exposure (Figure S22). The cooling performance of sh-ZANF was further examined in terms of preventing commercial hand-held cameras from overheating (Figure 5I). During thermal measurement, the cameras continuously recorded videos under sunlight to simulate real operational scenarios. The thermal infrared images and the real-time temperature profiles clearly indicate that the sh-ZANF-covered camera had much lower surface temperatures compared to the bare one, with a maximum temperature drop of 11.1 °C. Such efficient PDRC performance also dramatically extended the battery life of the camera by 31% (from 45 to 59 min).^72^ These findings demonstrate the suitability of sh-ZANF for use in portable electronic devices, ensuring better durability and operational efficiency in practical scenarios.

We also used the location of Taipei to assess the cooling requirements for human comfort throughout the year. Figure 5J displays the hour-month colored map of *T*_amb_ for Taipei, based on the World Meteorological Organization region and country weather data. The corresponding hour-month map of *T*_sh‑ZANF_ was obtained and shown in Figure 5K. Notably, sh-ZANF provided considerable cooling from April to October, which overlapped with the period of peak air-conditioning demand in subtropical areas (i.e., summer), thereby reducing energy consumption.

EnergyPlus simulations were employed for a more comprehensive house model to evaluate the year-round impact of sh-ZANF membranes on energy savings and CO_2_ emission reduction on a global scale (Figures 5L,M and S23). Sixteen representative cities were selected to reflect global climatic variations. The calculation results showed that sh-ZANF offered efficient energy savings in all considered cities, exceeding 10 MJ/m^2^/yr in tropical and subtropical regions. In addition, the energy savings of the building model were further converted into annual equivalent CO_2_ emission reductions for cities globally (Figure 5M). With the reduced electricity demand for cooling, the projected annual reduction in CO_2_ emission in these cities ranged from 11% to 27%. Cities with hot summers such as Phoenix, New Delhi, and Brasilia exhibited the most significant reductions (2.3, 2.1, and 2.0 tons/yr, respectively). These statistical simulations and calculations reveal the substantial potential of sh-ZANF to provide PDRC functions, emphasizing its capability as a carbon-neutral material that is particularly suitable for mitigating global warming in tropical and subtropical regions.

### 2.6 Durability of sh-ZANF for Long-Term Outdoor Applications

Weather resistance is critical for PDRC materials intended for long-term outdoor applications. Therefore, we evaluated the impact of various environments (*e.g.,* sludge buildup, sun damage, and acid rain corrosion) on the cooling performance of sh-ZANF. Figure 6A reveals that, owing to the fluorine-free surface coating of DMDMS and TEOS, sh-ZANF exhibited a high water contact angle (WCA) of 152°, whereas WCA was 154° for PFOT-modified sh-ZANF and 0° (hydrophilic) for the unmodified ZANF counterpart. In addition, the spectral measurements conducted before and after hydrophobic modification confirmed that the optical properties of ZANF remain nearly unchanged (Figure S24). After immersing sh-ZANF membranes in solutions at different pH, there were no obvious changes in their spectra or surface superhydrophobicity (Figures 6B,C and S25), indicating excellent resistance to acidic/basic corrosion. As illustrated in Figure 6D, mud residue left on the surface of sh-ZANF could be easily flushed off with water droplets, and this excellent self-cleaning ability allows the preservation of efficient cooling effect. In contrast, there was substantial sludge accumulation on the hydrophilic ZANF membrane, leading to significant absorption of solar power and loss of cooling performance. Moreover, sh-ZANF underwent additional one-month outdoor exposure, soil burial, abrasion, tape peel-off, and folding tests, maintaining hydrophobicity, optical properties, and antifouling performance, further confirming their robustness for real-world, long-term applications (see Figures S26–S30).

**Figure 6:**
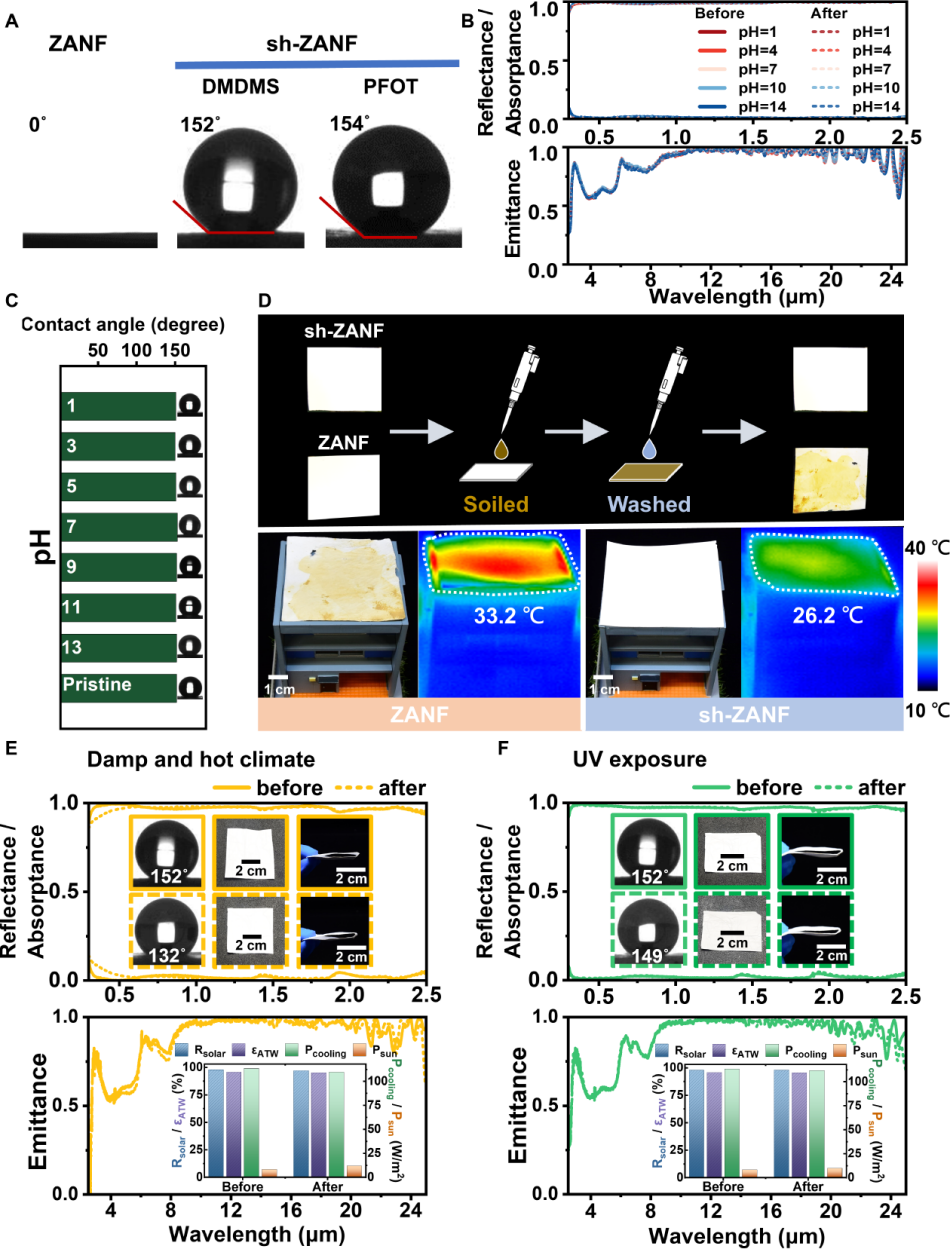
(A) Contact angles of the ZANF and sh-ZANF samples. (B) Solar reflectance/absorptance spectra and MIR emittance spectra, and (C) contact angles of sh-ZANF before and after immersion in solutions of various pH for 1 week. (D) Top: Photographs of ZANF and sh-ZANF samples treated with muddy water and then rinsed with water. Bottom: Photographs and thermal infrared images of the samples under direct sunlight. (E,F) Solar reflectance/absorptance and MIR emittance spectra of sh-ZANF before and after laboratory-scale accelerated aging tests in (E) damp heat and (F) UV precondition. Insets: the corresponding contact angles, appearance, flexibility, and PDRC performance.

Finally, laboratory-scale tests of accelerated aging, damp heat (DH), and UV preconditioning were conducted based on the IEC 61215-2:2021 standard^73^ to further examine the sustainable viability of sh-ZANF. This standard has been adopted a benchmark for evaluating photovoltaic modules and confirming their outdoor durability for up to 25 years under various climatic conditions in Europe and Australia.^74^ The DH test simulates the challenge of high-humidity and high-temperature conditions in tropical and subtropical climates, whereas the UV preconditioning test simulates prolonged exposure to intense UV radiation from sunlight. After the DH test, the membranes only showed neglectable changes in spectra and appearance (Figures 6E and S31, *R*_solar_ from 97.7% to 96.9% and ε_ATW_ from 95.6% to 95.2%). The hydrophobicity was also retained with a high WCA of 132°. The slight decrease in WCA may be attributed to the partial hydrolysis of siloxane bonds, leading to the formation of a few hydrophilic Si–OH groups on the surface.^75^ After exposure to intense UV light, sh-ZANF also maintained its spectral features and flexibility, with a trivial drop in WCA from 152° to 149° (Figures 6F and S32). These results strongly indicate the exceptional antiaging potential and weather resilience of sh-ZANF membranes, particularly in subtropical regions with harsh damp-heat environments. In addition, while current production of sh-ZANF nanofibers using electrospinning relies on a single-nozzle setup, advancements such as multineedle and needleless systems, expanded collector designs, and roll-to-roll techniques highlight the potential for efficient large-scale manufacturing.^76,77^ Coupled with its highly scalable production, flexibility, efficient radiative cooling performance, and ultrahigh fire retardancy, we conclude that sh-ZANF can serve as a highly efficient cooling enveloping material for prolonged use in diverse and challenging environments, significantly surpassing reported PDRC materials in terms of cooling performance and resistance to environmental aging (Table S2).

## 3 Conclusion

Superdurable electrospun sh-ZANF membranes were found to overcome the limitations of current polymeric- and ceramic-based materials for sustainable passive cooling in real-world applications. Theoretical and experimental spectra clearly revealed that the optimally engineered sh-ZANF has excellent PRDC capability, achieving exceptional *R*_solar_ of 97.7%, ε_ATW_ of 95.6%, and a theoretical *P*_cooling_ of 113.4 W/m^2^. Besides, sh-ZANF endured a record temperature of 1407 °C under butane flame, highlighting its potential in external building coverings. Moreover, it realized a substantial temperature drop of 6.6 °C in daytime and 4.6 °C in nighttime, with a maximum *P*_cooling_ of 125 and 112 W/m^2^, respectively. Building model, automobile model, and hand-held cameras covered by sh-ZANF all experienced significant cooling under sunlight exposure by 14.7 °C, 16.8 °C, and 11.1 °C, respectively, and the battery life of the camera was extended by 31% as a result. Finally, various extreme environmental tests confirmed the robust weather resilience of sh-ZANF, suggesting its suitability for long-term outdoor applications. In summary, the developed sh-ZANF functions as a flexible, ultralight, flame-retardant, and weather-resistant radiator for diverse exterior applications to mitigate global warming through efficient cooling.

## 4 Methods

### 4.1 Materials

Aluminum isopropoxide (AIP, ≥99.99%), aluminum chloride hexahydrate (AlCl_3_·6H_2_O, 99.99%), poly­(ethylene oxide) (PEO, *M*_w_ = 300 000 g/mol), zirconia acetate (15–17% Zr), ethanol (99.9%), acetic acid (≥99%), tetraethyl orthosilicate (TEOS, 99.999%), dimethoxydimethylsilane (DMDMS, 95%), isopropyl alcohol (IPA, 99.9%), hydrochloric acid (HCl, 37%), and perfluorooctyltriethoxysilane (PFOT, 97%) were purchased from Sigma-Aldrich. Double-distilled water (ddH_2_O) was purified via a Milli-Q system (resistivity: 18.2 MΩ cm). PVDF nanofibrous membranes were procured from Falco Tech Enterprise (Taiwan). Aluminum foil, aluminum sheets, painted stainless-steel sheets, and models for automobiles and houses were obtained from local markets.

### 4.2 Fabrication of sh-ZANF

ZANF was fabricated by modifying a previously reported protocol.^48^ First, an alumina precursor solution was prepared via hydrolysis and polycondensation by adding AIP (0.9 g) and AlCl_3_·6H_2_O (0.4 g) into a mixed solution of ddH_2_O/ethanol/acetic acid (2.8:3.5:1 w/w/w) and magnetic stirring at 800 rpm for 10 h. Subsequently, zirconia acetate was mixed with the alumina precursor solution (Al = 0, 6.5, 9, and 11.5 wt %) and PEO powder (1 wt %) and magnetically stirred at 800 rpm for 8 h to obtain the spinning solution. This solution was immediately transferred to syringes. Electrospinning was performed at a uniform flow rate of 1 mL/h, and a high voltage of 12 kV was applied to the needle tip. The resulting ZrO_2_/Al_2_O_3_/PEO hybrid nanofibrous membranes were harvested from the surface of the collection plate/rotor, calcined in air at 800 °C (heating rate: 2 °C/min) for 2 h, and naturally cooled to room temperature. Then, the membranes were impregnated with a DMDMS/TEOS/IPA solution (1.16/0.75/16 v/v/v) for 2 h and dried in air overnight to obtain sh-ZANF. To compare surface modification methods, the ZANF membranes were also impregnated with a PFOT solution. Specifically, ethanol (24 mL) and ddH_2_O (1 mL) were mixed, added with HCl (2 μL), and stirred for 1 min. This mixed solution was added with TEOS (1.2 mL) and PFOT (0.24 mL), followed by vigorous stirring at 360 rpm for 1 h. Nanofibrous membranes were impregnated this PFOT solution and dried under the same conditions. The membrane basis weights of 4, 6, 12, 18, 23, and 28 mg/cm^2^ correspond to thicknesses of approximately 60, 125, 240, 355, 475, and 585 μm, respectively.

### 4.3 Fabrication of SiO_2_ Nanofibrous Membranes

A SiO_2_ precursor solution was prepared at the molar ratio of TEOS:ddH_2_O:H_3_PO_4_ = 1:11:0.01 and stirred for 12 h at 800 rpm and room temperature. A PVA solution was prepared by dissolving PVA powder (10 wt %) in ddH_2_O at 80 °C and stirring for 6 h at 800 rpm. The SiO_2_ precursor solution was added to the PVA solution at 1:1 w/w and stirred for 4 h at 800 rpm to obtain a spinning solution, which was stored at room temperature for 16 h before transfer to a syringe. During electrospinning, the solution flow rate was fixed at 1 mL/h, and a high voltage of 15 kV was applied to the needle tip. The obtained TEOS/PVA hybrid membranes were calcined in air at 800 °C (heating rate: 3 °C/min) for 2 h to obtain the SiO_2_ nanofibrous membranes.

### 4.4 Fabrication of PEO Nanofibrous Membranes

PEO powder (10 wt %) was dissolved in ddH_2_O at room temperature, followed by continuous stirring for 24 h. This solution was immediately transferred to a syringe and used for electrospinning at a uniform flow rate of 1 mL/h. A high voltage of 12 kV was applied to the needle tip. The nanofibrous PEO membranes were harvested from the surfaces of the collection plate and rotor.

### 4.5 Characterization

Microstructural morphologies of sh-ZANF and ZrO_2_ nanofibers were observed using field emission scanning electron microscopy (FE-SEM; JEOL, JSM-7000F) coupled with energy dispersive spectrometry (EDS; JEOL, ARM200F) and field emission transmission electron microscopy (FE-TEM; JEOL, JEM-F200). The distribution of fiber diameter was estimated from the SEM images using ImageJ software. The crystalline phases of sh-ZANF and ZrO_2_ nanofibers were characterized using X-ray diffraction (XRD; Bruker D8 Discover) and Raman spectroscopy (B&W Tek, i-Raman Plus). Elemental identification was conducted using inductively coupled plasma-mass spectrometry (ICP-MS; Thermo Fisher Scientific, iCAP TQ). The chemical states of the elements were analyzed using X-ray photoelectron spectroscopy (XPS; ULVAC-PHI). The tensile strengths of sh-ZANF (5 cm × 3 cm × 400 μm) and ZrO_2_ nanofiber (4 cm × 3 cm × 400 μm) membranes were measured with a tensile testing machine (Shimadzu, Autograph AGS-X) with the tensile speed set at 5%/min. The solar spectra of sh-ZANF were obtained using a UV–vis-NIR spectrophotometer (Hitachi, UH4150) coupled with an integrating sphere (diameters of 6 and 15 cm) and Al_2_O_3_ white reference plates. The MIR spectra were measured using a Fourier-transform infrared (FTIR) spectrometer (Bruker, Invenio R) equipped with a gold-coated integrating sphere and gold-coated reference plate. The emissivity (ε) of sh-ZANF at variable angles was measured using a blackbody emitter simulator (Infrared System Development Corporation, IR-563/301 Blackbody Source and IR-301 Blackbody controller). The thermal stability of sh-ZANF was evaluated via thermogravimetric analysis (TGA; Mettler-Toledo, 2-HT) from 30 to 1100 °C at a heating rate of 10 °C/min. The heat distribution of sh-ZANF at high temperatures was monitored using a thermal infrared imaging camera (AVIO, InfReC Thermal GEAR G100EX). Water contact angles were measured using a contact angle meter (First Ten Angstroms, FTA 1000 B). The porosity (ρ) was measured using a mercury porosimeter (Micromeritics AUTOPORE 9520).

### 4.6 Optical Simulations

The effective complex refractive index (*N* = *n* + *ik*) of ZANF was calculated using the Maxwell Garnett effective medium expression.^78^ The dielectric constant of effective medium (ϵ) was obtained by

2 $$f_{z}\frac{\epsilon_{z}-\epsilon }{\epsilon_{z}+2\epsilon }+f_{a}\frac{\epsilon_{a}-\epsilon }{\epsilon_{a}+2\epsilon }=0$$

where *f*_z_ and *f*_a_ are the volume fractions of ZrO_2_ and Al_2_O_3_ (*f*_z_ = 0.85, *f*_a_ = 0.15), and ϵ_z_ and ϵ_a_ are the dielectric constants, respectively. The complex refractive index was obtained from the effective dielectric constant using the following formula:

3 $$\epsilon_{\mathrm{real}}=n^{2}-k^{2}$$

4 $$\epsilon_{\mathrm{imaginary}}=2ink$$

where *n* is the refractive index and *k* stands for the extinction coefficient.

The Mie scattering and absorption efficiencies of a single fiber were simulated in a previous work^79^ using MATLAB (MathWorks, R2021a) as a function of the wavelength and fiber diameter. The corresponding complex refractive indices of the ceramic fibers are provided in Figure S3, assuming a background refractive index of 1 for air. In addition, 2D-FDTD simulations (RSoft) were used to determine the near-field optical properties of ZANF membranes of different membrane thicknesses. The cross-section of the membranes was determined by randomly placing *M* circles in an area of *A*, and the porosity (*P*) of the ZANF structure was defined as

5 $$Ρ=1-\frac{M\pi r^{2}}{A}$$

where *r* is the fiber radius. The membrane thicknesses were set from 10 to 700 μm, the simulation domain had a width of 8 μm, and the wavelengths ranged from 0.3 to 25 μm. The diameter distribution of the randomly placed circles was set at 400 ± 60 nm. Figure S5 shows the optical simulation setup, including the material, plane-wave source, reflection, and transmission monitors. The reflectance (R), transmittance (T), and absorptance (A) were obtained by dividing the reflected (*P*_R_), transmitted (*P*_T_), and absorbed (*P*_A_) power by the incident power (*P*_incident_), respectively.

6 $$R=\frac{P_{R}\left(\lambda \right)}{P_{\mathrm{incident}}\left(\lambda \right)},,T=\frac{P_{T}\left(\lambda \right)}{P_{\mathrm{incident}}\left(\lambda \right)},,A=\frac{P_{A}\left(\lambda \right)}{P_{\mathrm{incident}}\left(\lambda \right)}$$

### 4.7 Calculation of Radiative Cooling Performance

The following equations were used to calculate the radiative cooling performance and implemented in Matlab (MathWorks, R2021a). Based on the reflectance data, the average solar reflectance (*R*_solar_) and average reflectance (*R*_avg_) were calculated as

7 $$R_{\mathrm{solar}}=\frac{\int_{0.3\mu m}^{2.5\mu m}R\left(\lambda \right)I_{AM1.5G}\left(\lambda \right)d\lambda }{\int_{0.3\mu m}^{2.5\mu m}I_{AM1.5G}\left(\lambda \right)d\lambda }$$

8 $$R_{\mathrm{avg}}=\frac{\int_{0.3\mu m}^{2.5\mu m}R\left(\lambda \right)d\lambda }{\int_{0.3\mu m}^{2.5\mu m}d\lambda }$$

where *R*(λ) represents the material reflectance at wavelength λ, and *I*_AM1.5G_(λ) is the solar illumination spectra with air mass 1.5.

The ATW emittance (ε_ATW_) and average MIR emittance (ε_avg_) were calculated as follows:

9 $$\varepsilon_{\mathrm{ATW}}=\frac{\int_{8\mu m}^{13\mu m}I_{BB}\left(T,\lambda \right)\varepsilon \left(\lambda \right)d\lambda }{\int_{8\mu m}^{13\mu m}I_{BB}\left(T,\lambda \right)d\lambda }$$

10 $$\varepsilon_{\mathrm{avg}}=\frac{\int_{8\mu m}^{25\mu m}\varepsilon \left(\lambda \right)d\lambda }{\int_{8\mu m}^{25\mu m}d\lambda }$$

where $I_{\mathrm{BB}}\left(T,\lambda \right)=\frac{2hc^{2}}{\lambda^{5}}\frac{1}{e^{\mathrm{hc}/\left(\lambda k_{B}T\right)}-1}$ is the spectral irradiance of a blackbody at temperature *T*. Additionally, *h*, *c*, and *k*_B_ are Plank’s constant, speed of light in vacuum, and Boltzmann constant, respectively. ε­(λ) represents the material emittance at wavelength λ.

The theoretical *P*_cooling_ of a PDRC system can be determined by balancing the energies defined in eq 1 The radiation power of the material [*P*_rad_(*T*)] at surface temperature *T* is expressed by eq 11:

11 $$P_{\mathrm{rad}}\left(T\right)=A\int d\Omega \cos\theta \int_{0}^{\infty }I_{BB}\left(T,\lambda \right)\varepsilon \left(\lambda ,\theta \right)d\lambda$$

Here, *A* is the surface area of the material, $\int d\Omega =2\pi \int_{0}^{\pi /2}d\theta \sin\theta$ is the angular integral over a hemisphere, and ε*(*λ, θ*)* is the spectral angular emissivity of the PDRC material depending on the wavelength. The absorbed solar power (*P*_sun_) can be expressed by eq 12

12 $$P_{\mathrm{sun}}=A\int_{0}^{\infty }\varepsilon \left(\lambda ,\theta_{\mathrm{sun}}\right)I_{AM1.5G}\left(\lambda \right)d\lambda$$

Moreover, radiation absorbed by the material from the atmosphere [*P*_atm_(*T*_amb_)] can be defined by eq 13:

13 $$P_{\mathrm{atm}}\left(T_{\mathrm{amb}}\right)=A\int d\Omega \cos\theta \int_{0}^{\infty }I_{BB}\left(T_{\mathrm{amb}},\lambda \right)\varepsilon \left(\lambda ,\theta \right)\varepsilon_{\mathrm{atm}}\left(\lambda ,\theta \right)d\lambda$$

where *T*_amb_ is the ambient temperature, $\varepsilon_{\mathrm{atm}}\left(\lambda ,\theta \right)=1-{t\left(\lambda \right)}^{1/\cos\theta }$ is the wavelength-dependent angular emissivity of atmosphere, and *t*(λ) is the transmittance of atmosphere in the zenith direction. According to Kirchhoff’s law, absorptivity is equal to emissivity at the same wavelength and direction under thermal equilibrium conditions. Thus, the derivation can be accomplished by substituting the absorptivity of the material with its emissivity, ε­(λ, θ), in eq 11 and eq 13. The nonradiative heat power caused by conduction and convection can be calculated using eq 14:

14 $$P_{\mathrm{cond}+\mathrm{conv}}\left(T,T_{amb}\right)=Ah_{c}\left(T_{amb}-T\right)$$

Here, *h*_c_ *= h*_cond_ *+ h*_conv_ is the comprehensive nonradiative heat transfer coefficient derived by considering the conductive heat exchanges.

### 4.8 Outdoor Radiative Cooling Measurement

The assembled apparatus consisted of a foam cover with aluminum foil (to reflect radiation from the surroundings) and a layer of PE film applied atop the window (to reduce heat convection). Inside the apparatus, uniform sample temperatures were verified by measurements using K-type thermocouples in contact with the copper plate under the samples. The data were collected continuously in real time using a multichannel temperature data logger (Pico Technology, TC-08). The ambient temperatures were measured using K-type thermocouples covered with Al foil. A PI heater was placed beneath the copper plate. The feedback-controlled mechanism was driven by a source meter (Keithley, 2400) using LabVIEW to maintain the sample temperature equal to ambient temperature through active feedback control of the integrated heater. The required heating power under thermal equilibrium was recorded as the *P*_cooling_ of sh-ZANF.^10,33^ Solar irradiance was recorded using a pyranometer data logger (METEON, CMP10), and both the relative humidity and wind speed were recorded using a weather station (Ecowitt, HP3501). Infrared images of the building/automobile models and hand-held cameras (SJCAM C100) were obtained with a thermal infrared imaging camera.

### 4.9 Calculations of Worldwide Energy Consumption

An energy-saving simulation was conducted using the Building Energy Optimization Tool (BEopt, version 2.7). The building energy saving model followed the standards for energy efficiency requirements^80^ and was estimated using a whole-building energy simulation. The building had the dimensions of 46 m (length) × 17 m (width) × 3 m (height). Building energy-saving performance was evaluated as the difference between the custom-defined sh-ZANF and dark metal. In the simulation, sh-ZANF was applied as the outer layer of the roof. The optical properties (solar absorptance/MIR emittance) of sh-ZANF and dark metal were set at (0.008/0.97) and (0.9/0.9), respectively. Additional detailed information is provided in Table S3.

### 4.10 Flame-Resistance Experiments

In the flame-resistance experiments, a blowtorch was applied to the sample, and the sample appearance over time was recorded using a digital camera. The temperature and thermal infrared images of sh-ZANF were recorded using a thermal infrared imaging camera (emissivity = 1.0). To validate the flame resistance of sh-ZANF, bending tests were performed to evaluate the flexural strength and modulus both before and after direct flame exposure according to ASTM D790 methods.^81^ To assess the potential of sh-ZANF as a fireproofing material for protecting construction materials, the temperature–time relationship in a furnace was evaluated following the ISO 834 standard (Figure S19A).^68^

### 4.11 Anticorrosion Assessment

sh-ZANF was immersed in aqueous solutions at pH = 1, 3, 5, 7, 10, and 14 for a week to investigate its chemical stability under corrosive conditions (such as acidic rain). The water contact angle and solar and MIR spectra were measured before and after immersion treatment.

### 4.12 Environmental Aging Tests

To quantitatively assess the resistance of sh-ZANF against environmental aging, the Industrial Technology Research Institute (ITRI, Taiwan) was commissioned to conduct both DH and UV preconditioning tests under laboratory conditions in accordance with the IEC 61215-2:2021 standard.^74^ For the DH test, sh-ZANF was exposed for 1000 h to 85% relative humidity and 85 °C ± 2 °C. For the UV preconditioning test, it was subjected to a cumulative UV irradiation dose of 5.4 × 10^7^ J/m^2^, encompassing a wavelength range of 0.28–0.4 μm and comprising 6.1% UV–B irradiance (0.28–0.32 μm).

## Supplementary Material



## Notes

Discussions on 2D-FDTD simulations of ZANF, cost analysis of sh-ZANF, blowtorch test for sh-ZANF, comparison of cooling performance between sh-ZANF and other construction materials, durability evaluations of sh-ZANF, high solar reflectance of sh-ZANF, Reststrahlen effect on ceramic thin films; fabrication scheme and ICP-MS analysis of sh-ZANF; optical constants and calculated absorption efficiencies of ZANF, ZrO_2_, Al_2_O_3_, and SiO_2_ nanofibers; schematic setup in 2D-FDTD simulation; mesh convergence test in 2D-FDTD simulation; additional calculated spectra of ceramic nanofibrous membranes; additional calculated spectra of ZANF with various thicknesses; additional measured spectra of ZANF with different Al_2_O_3_ weight ratios; XPS analysis of ZANF; HR-TEM images and XPS analysis of ZrO_2_; additional measured spectra of sh-ZANF with various base weights; SEM images, diameter distributions and additional measured spectra of SiO_2_, PVDF, and PEO nanofibrous membranes; measured spectra and corresponding *R*_solar_, ε_ATW_, *P*_cooling_, and *P*_sun_ values for five replicate sh-ZANF membranes; additional thermal infrared images of sh-ZANF exposed to blowtorch flame; TGA analysis of sh-ZANF; combustion test fir aluminum sheets; additional information for the ISO 834 standard; environment conditions during the outdoor thermal measurements; schematic representation of interior temperature measurement; additional outdoor thermal measurements for sh-ZANF and other construction materials; scheme of the model in EnergyPlus; measured spectra of ZANF before and after hydrophobic treatment; additional durability evaluations through pH immersion, one-month outdoor exposure, soil burial, abrasion, tape peel-off, folding cyclic, damp heat, UV preconditioning tests; absolute reflectance spectrum of an Al_2_O_3_ white plate; calculated spectra of ceramic thin films; estimated material cost of sh-ZANF and Al_2_O_3_ nanofiber; comparison of durable PDRC materials in previous and current studies (PDF)
