# Supplementary material for: Superdurable, Flexible Ceramic Nanofibers for Sustainable Passive Radiative Cooling
Source: ACS Nano. 2025 Jul 31;19(31):28280–94. doi: 10.1021/acsnano.5c05958 (PMC12356120; doi:10.1021/acsnano.5c05958)
Supplement: Supplementary file 1 [file nn5c05958_si_001.pdf]

Supporting Information

Superdurable, Flexible Ceramic Nanofibers for Sustainable Passive Radiative Cooling

Dai-Chi Chen[a,‡], Ching-Wen Hwang[a,‡], Ching Yin Chang[a], Chia-Ling Kuo[a], Hsuen-Li Chen[b,c], Pin-Hui Lan[a], Meng-Ting Tsai[a], Tzu-Wei Wang[d,e], and Dehui Wan[a,*]

[a]*Institute of Biomedical Engineering, National Tsing Hua University, Hsinchu, 300044, Taiwan*

[b]*Department of Materials Science and Engineering, National Taiwan University, Taipei, 106319, Taiwan*

[c]*Center of Atomic Initiative for New Materials, National Taiwan University, Taipei, 106319, Taiwan*

[d]*Department of Materials Science and Engineering, National Tsing Hua University, Hsinchu City 300044, Taiwan*

[e]*Institute of Oral Biology, College of Dentistry, National Yang Ming Chiao Tung University, Taipei 112304, Taiwan*

[‡]These authors contributed equally to this work.

[*]To whom correspondence should be addressed.

E-mail: dhwan@mx.nthu.edu.tw

Keywords: Ceramic nanofibers, passive radiative cooling, flame resistance, electrospinning, environmental aging resistance

S1. 2D-FDTD simulations for ZANF membranes

To facilitate systematic parameter exploration and optimization during the initial design phase, we employed 2D-FDTD simulations for comprehensive optical analysis (**Figure S5**)—a strategy previously validated in the literature[1-3]. This approach offers substantial computational efficiency compared to full 3D simulations, which are often computationally prohibitive due to the stochastic and aperiodic architecture of nanofibrous membranes. Prof. Yuan Yang and co-workers have demonstrated that, despite reduced dimensionality, 2D models reliably capture the essential scattering behavior of 3D systems, as individual pores exhibit comparable scattering efficiencies across various pore radii and wavelengths in both frameworks[1]. Therefore, the optimal fiber size distribution derived from 2D simulations is expected to closely approximate that of 3D cases. While we acknowledge that differences in circle/fiber density between 2D and 3D models may lead to deviations in absolute values, the qualitative trends are expected to remain consistent, making 2D simulations well-suited for investigating the optical behavior of nanofibrous membranes.

Also, we conducted mesh convergence testing in our 2D FDTD simulations for ZANF membranes with thicknesses of 10 μm and 50 μm, varying the grid size from 15 nm to 240 nm. As shown in **Figure S6**, the simulated solar spectra and the calculated R_{solar} values converged when the grid size was reduced below 60 nm for both membrane thicknesses. Based on these results, a grid size of 30 nm was selected for all subsequent simulations.

S2. Estimated material cost analysis of sh-ZANF

In this study, electrospinning enables the fabrication of high-quality sh-ZANF nanofibers with precise control over diameter, morphology, and composition, essential for achieving their excellent thermal and optical properties. To further evaluate the scalability of sh-ZANF for future mass production, we conducted a cost analysis of its primary materials (**Table S1**). The composition of 1 g of sh-ZANF includes 91% ZrO_2 and 9% Al_2O_3. The ZrO_2 is derived from $Zr(Ac)_4$, requiring 2.417 g at a cost of USD $1.09. The Al_2O_3 is sourced from $AlCl_3$•$6H_2O$ and AIP, requiring 0.136 g of $AlCl_3$•$6H_2O$ at USD $0.53 and 0.305 g of AIP at USD $3.87, respectively.

Consequently, the total cost for producing 1 g of sh-ZANF is USD \$5.50. In comparison, recently reported Al_2O_3 nanofibers[4] require 1.584 g of $AlCl_3{\bullet}6H_2O$ at USD \$6.22 and 3.325 g of AIP at USD \$42.18, resulting in a total production cost of USD \$48.40 per gram. This significant cost difference highlights the economic advantage of sh-ZANF for potential large-scale production.

S3. Estimated surface temperatures of sh-ZANF in the blowtorch test

In this study, under extreme thermal shock conditions, the surface temperatures of sh-ZANF were determined from thermal infrared images captured using an IR camera. Temperature readings were calculated assuming an emissivity of 1.0. In **Figure S16,** four independent blowtorch tests were conducted, yielding maximum surface temperatures of 1399 °C, 1404 °C, 1407 °C, and 1407 °C, respectively. In contrast, the emissivity of sh-ZANF was measured to be 0.956 at room temperature. When this value was applied for emissivity correction, the corresponding maximum surface temperatures increased to 1425 °C, 1429 °C, 1432 °C, and 1432 °C. However, given that emissivity can vary with temperature and in-situ emissivity measurement under such extreme conditions remains challenging, the values calculated using emissivity = 1.0 were adopted as conservative estimates. Additionally, as the tests were conducted indoors under windless conditions, the flame-induced temperature distribution appeared as uniform concentric rings across the sample surface. Notably, the flame temperature was higher than the measured surface temperatures of sh-ZANF.

S4. Comparison of cooling performance between sh-ZANF and other construction materials

To evaluate its real-world applicability, we further conducted sunlight exposure tests on a wooden cabin covered with sh-ZANF and various common building materials, including concrete block, red tile, and white tile, for durations of 10, 20, and 30 min (**Figure S22**). After 30 min of exposure, the bare wood cabin showed a significant temperature increase to 36°C, even at an ambient temperature of ~20°C. All other building materials, including white tiles similar in color to sh-ZANF, reached surface temperatures exceeding 25°C. In contrast, sh-ZANF demonstrated superior cooling performance, reaching the lowest temperature of 21.4°C. These results indicate

that applying sh-ZANF to actual wooden cabins or other construction materials can achieve a temperature reduction of up to 15°C, demonstrating its feasibility for future applications in the construction market.

S5. Comprehensive durability evaluations of sh-ZANF

The durability of sh-ZANF was thoroughly evaluated through comprehensive tests, including one-month outdoor exposure and soil burial to assess weather resistance, as well as abrasion, tape peel-off and folding tests for mechanical durability. Surface hydrophobicity and solar/MIR spectra were monitored throughout the evaluations.

In the outdoor exposure test, sh-ZANF was subjected to wind, sunlight, and rain for one month (2024.12.4–2025.1.1). As shown in **Figure S26**, no significant changes were observed in contact angles or spectral properties. During the soil burial test, both sh-ZANF and wood samples were placed in harsh, dirty conditions. After 30 days, **Figure S27** revealed that the surface of sh-ZANF remained clean, while soil penetrated the wood, demonstrating excellent anti-fouling properties. By preventing dirt accumulation, sh-ZANF avoids increased solar absorption and preserves cooling performance. Mechanical resilience was further assessed through abrasion and tape peel-off tests. For the abrasion test, following methods from previous studies[5, 6], a 20 g weight was applied to sh-ZANF placed face-down on P400 sandpaper. The sample was moved 10 cm, rotated 90°, and moved another 10 cm to complete one cycle, with 20 cycles performed in total (**Figures S28A and S28B**). The tape peel-off test, conducted using 3M Scotch Invisible Tape (model 810D) and methods from prior studies[5], involved adhering the tape to the surface and peeling it off to evaluate adhesion properties (**Figures S29A and S29B**). Despite these rigorous tests, sh-ZANF maintained its hydrophobic and optical performance, as evidenced in **Figures S28 and S29**. In addition, folding cyclic tests were conducted, in which each fold was maintained at a 180-degree angle (**Figure S30A**), and a total of 100 cycles were performed. After testing, sh-ZANF showed no notable changes in appearance (**Figure S30B**) or optical spectra (**Figure S30C–I**). These findings collectively confirm the exceptional durability and practicality of sh-ZANF for

real-world, long-term applications.

S6. High solar reflectance of **sh-ZANF**

The reflectance measurements using an integrating sphere yield values greater than 100% because the comparison is made relative to an Al_2O_3 white plate standard (Hitachi, P/N 210-0740). Consequently, when the reflectance of the sample exceeds that of the Al_2O_3 white plate, values greater than 100% may be observed. According to the absolute reflectivity data provided by Hitachi Astemo supplier (**Figure S33**), this effect may be due to weak absorption in the white plate in the UV band, potentially caused by binders (*e.g.*, silicates) used to enhance its mechanical properties.[7, 8] The feature of solar reflectivity has been observed in other research articles,[9-11] when a strong sunlight scatter was employed in their PDRC design.

S7. Disscusion of the Reststrahlen effect on MIR emittance ceramic thin films

In polar dielectric materials, the Reststrahlen effect arises from strong optical phonon absorption, which leads to a sharp increase in the *k* within a specific spectral range. This elevated *k* value results in high reflectance and thus significantly suppresses emittance in the corresponding region.[12] To further elucidate the Reststrahlen effect, we utilized Film Wizard to simulate the reflectance, transmittance, and emittance of 100 μm thin solid films composed of various ceramic materials within the ATW region (**Figure S34**). For instance, in SiC—a material with a pronounced Reststrahlen band—high *k* values lead to nearly total reflectance in the 10.5–12.5 μm wavelength range (**Figure S34B and S34D**), resulting in exceptionally low emittance (<5%) (**Figure S34F**). Similarly, Al_2O_3 and SiO_2 exhibit significantly high *k* values within the ATW spectral range (*c.a.*, 8–10 μm for SiO_2 and 11–13 μm for Al_2O_3), leading to substantial reflectance (R_{max} > 70%) and imposing a strict upper limit on their emittance (**Figure S34D and S34F**). In contrast, pure ZrO_2, the primary constituent of sh-ZANF, maintains consistently moderate *k* values across the entire ATW range, allowing for a significantly higher maximum emittance exceeding 90%. Although this analysis is based on thin-film optics, the underlying Reststrahlen mechanism

extends to nanofibrous membranes, explaining why ZANF achieves superior thermal emission performance compared to Al_2O_3 and SiO_2. This is particularly evident when considering our experimental results alongside the latest reported Al_2O_3 nanofibers[4], further reinforcing the advantages of sh-ZANF in passive radiative cooling applications.

To better illustrate this concept, we also simulated, as an example, 50 µm-thick solid films with varying *k* values, while keeping the *n* fixed at 1.5 across the 8–13 µm atmospheric transparency window. As shown in **Figure S35**, the initial increase in *k* (up to ~10^{-4}) enhances emittance (**Figure S35D**), which in turn decreases transmittance (**Figure S35C**). Once transmittance drops to zero, emittance reaches saturation. However, as *k* increases beyond 1, reflectance begins to rise sharply (**Figure S35B**), causing a significant decline in emittance **(Figure S35D**). **Figure S35E** indicates that high *k* values lead to increased reflectance, thereby limiting the maximum achievable emittance. In contrast, low *k* values result in insufficient emittance unless the material thickness is substantially increased. Materials with moderate *k* values, however, can achieve higher emittance even at relatively small thicknesses, offering a more efficient balance between material usage and radiative performance.

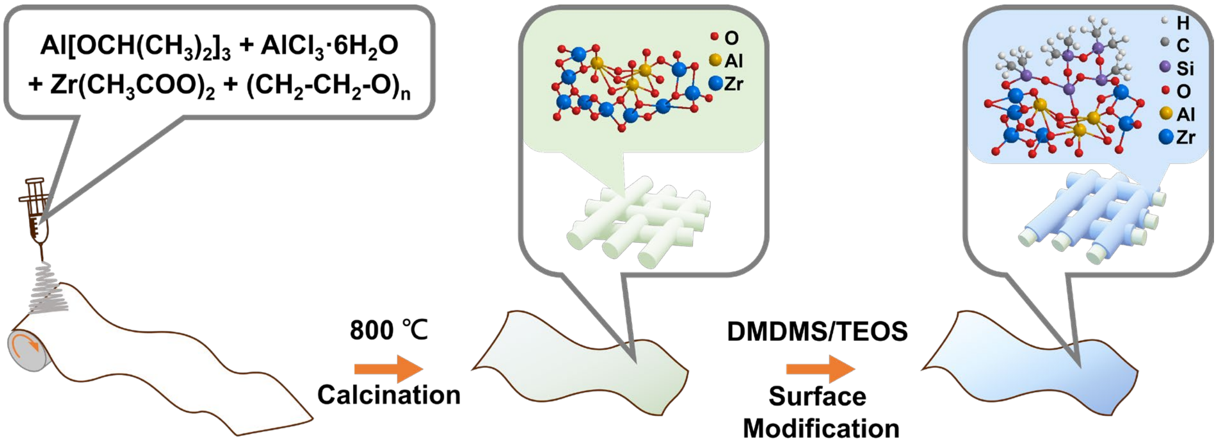

Figure S1. Schematic representation of the scalable fabrication process of sh-ZANF through the steps of sol-gel, electrospinning, calcination, and fluorine-free hydrophobic modification.

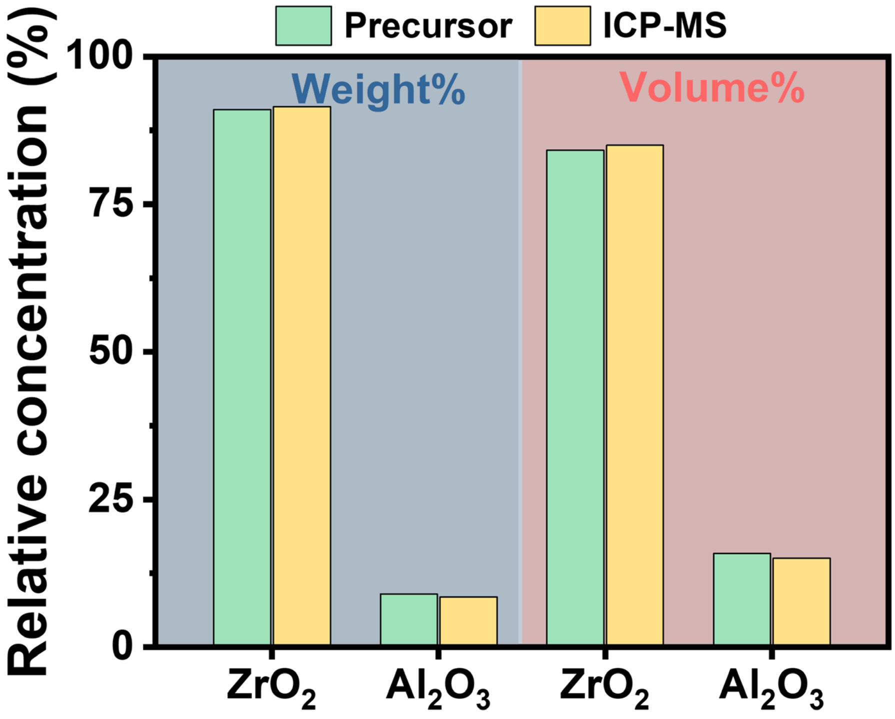

Figure S2. Weight and volume ratios of ZrO_2 and Al_2O_3 in sh-ZANF obtained from the theoretical precursor concentrations and ICP-MS analysis.

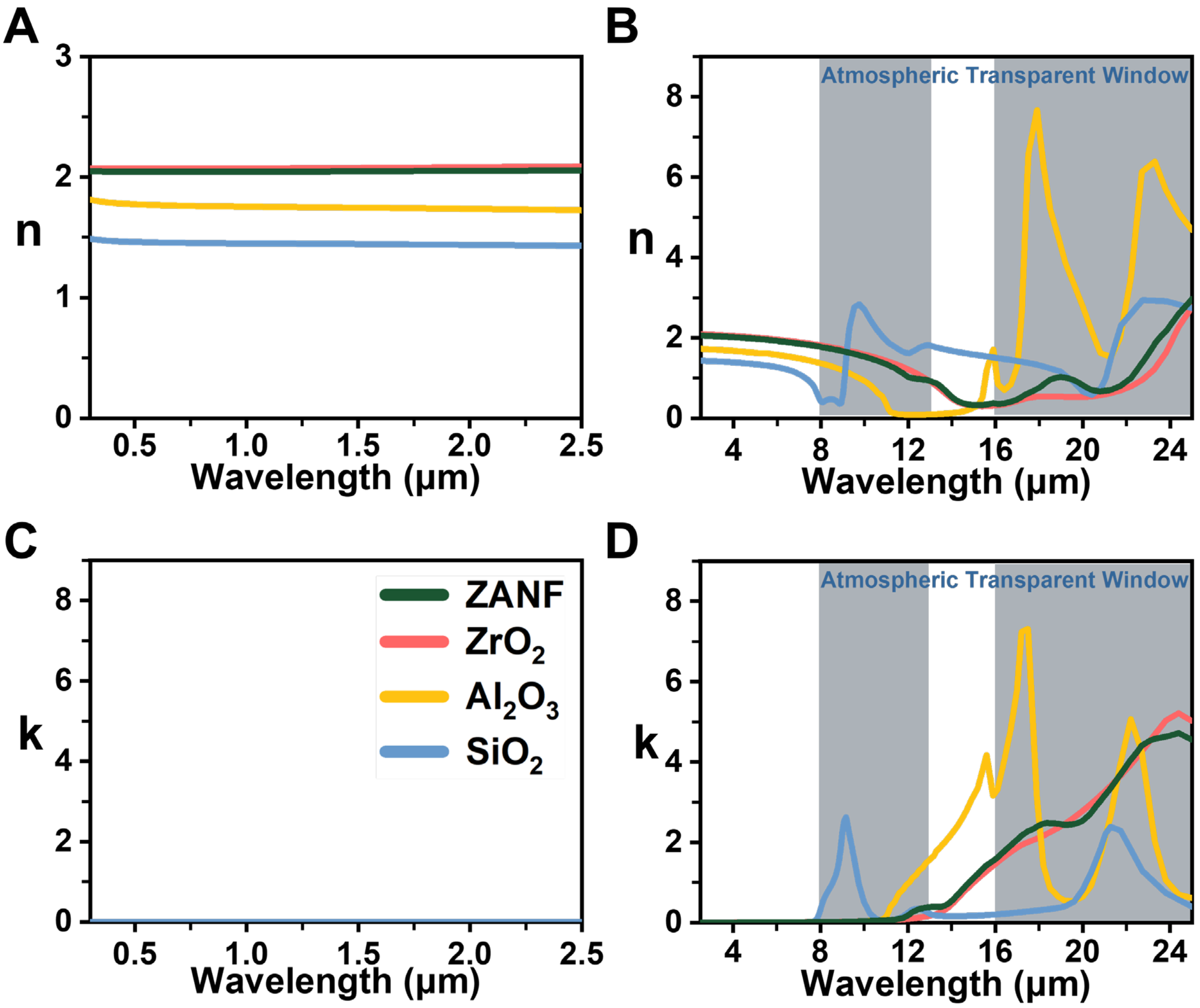

Figure S3. (A, B) refractive indices (*n*) and (C, D) extinction coefficients (*k*) of ZANF, ZrO_2, Al_2O_3, and SiO_2 in the (A, C) solar and (B, D) MIR bands.

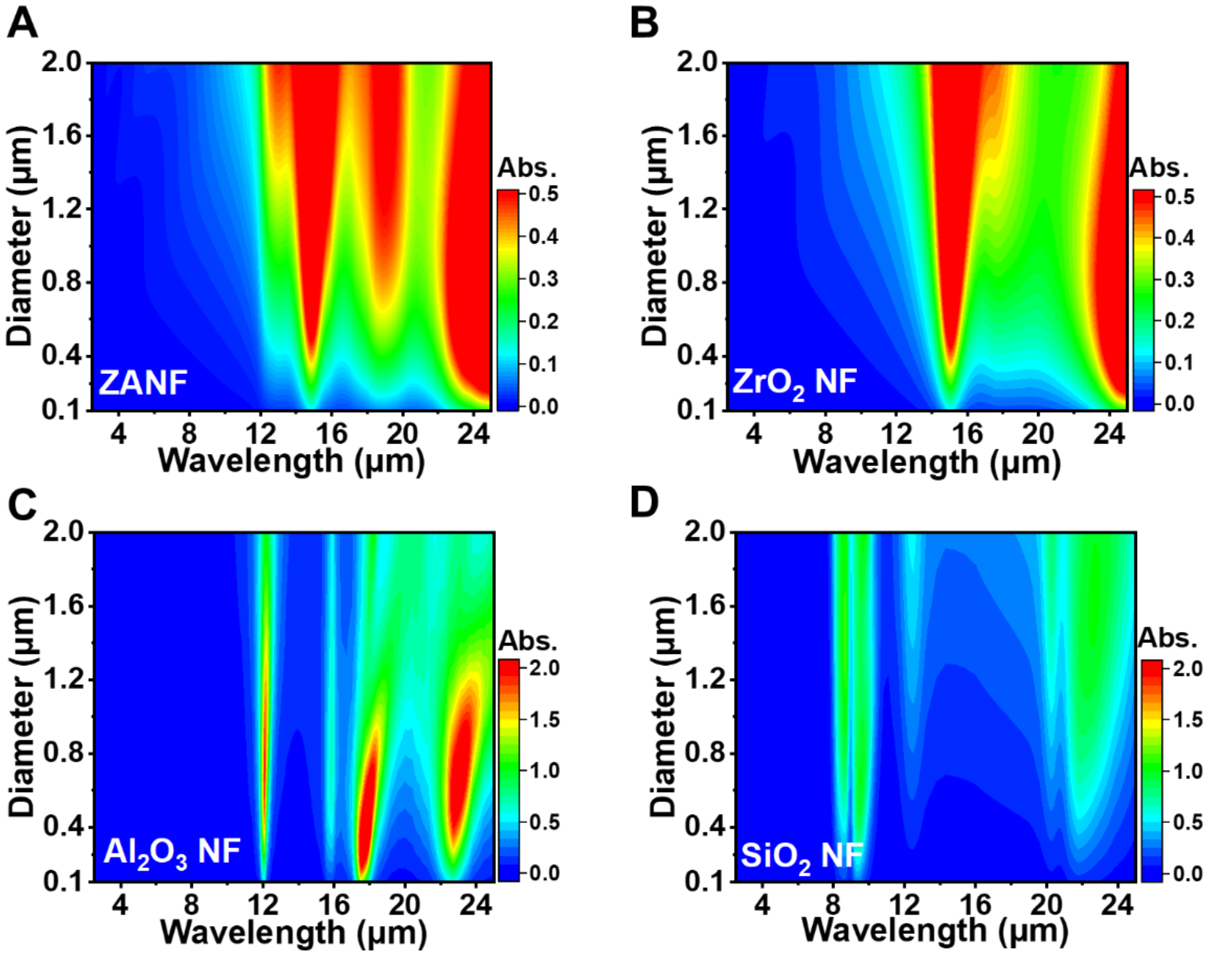

Figure S4. Calculated absorption efficiencies of (A) ZANF, (B) ZrO_2, (C) Al_2O_3, and (D) SiO_2 nanofibrous membranes in the MIR region.

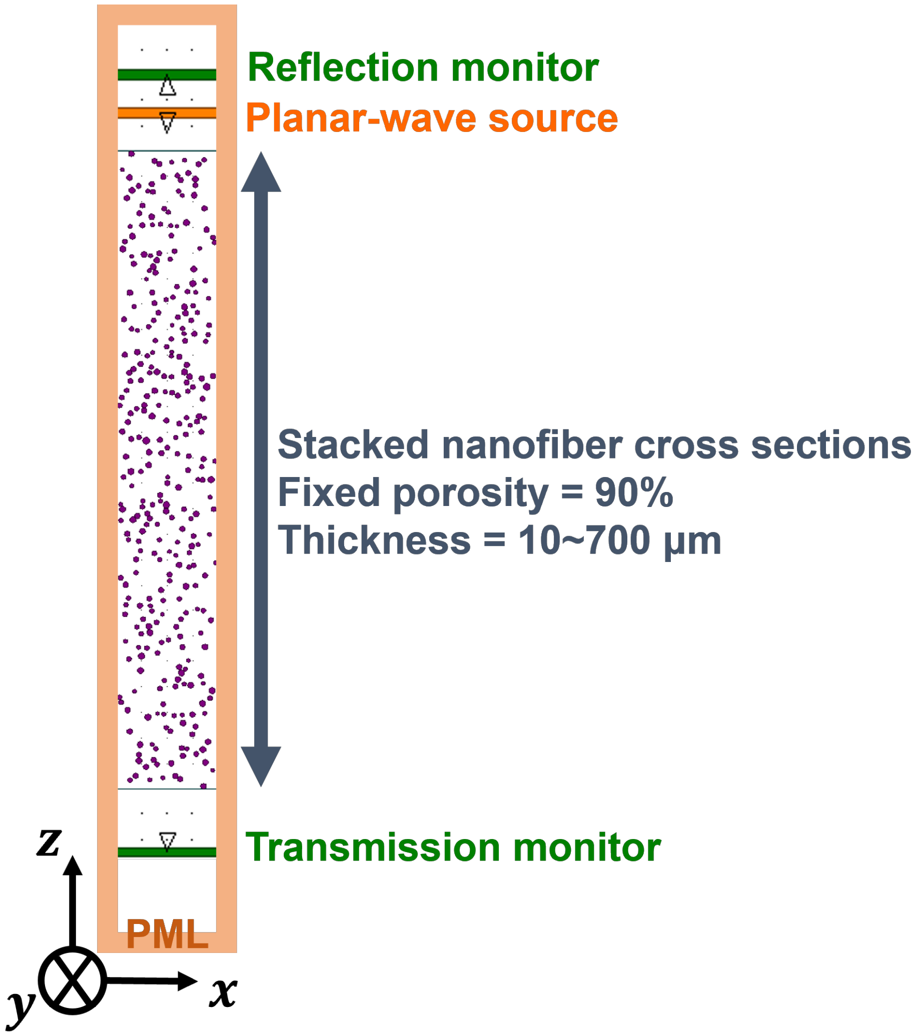

Figure S5. Schematic representation of the setup in 2D-FDTD optical simulation. Cross-sectional view of the simulation domain for ZANF, Al_2O_3, and SiO_2 nanofibers, with a diameter of 400 ± 60 nm and a porosity of 90%. The three hollow triangles on the planar-wave source, reflection monitor, and transmission monitor indicate the direction of light propagation. The thickness of the nanofibrous membrane varied from 10 to 1000 μm. PML = perfectly matched layer that was set around the material domain. The grid size for all FDTD simulations was 30 nm.

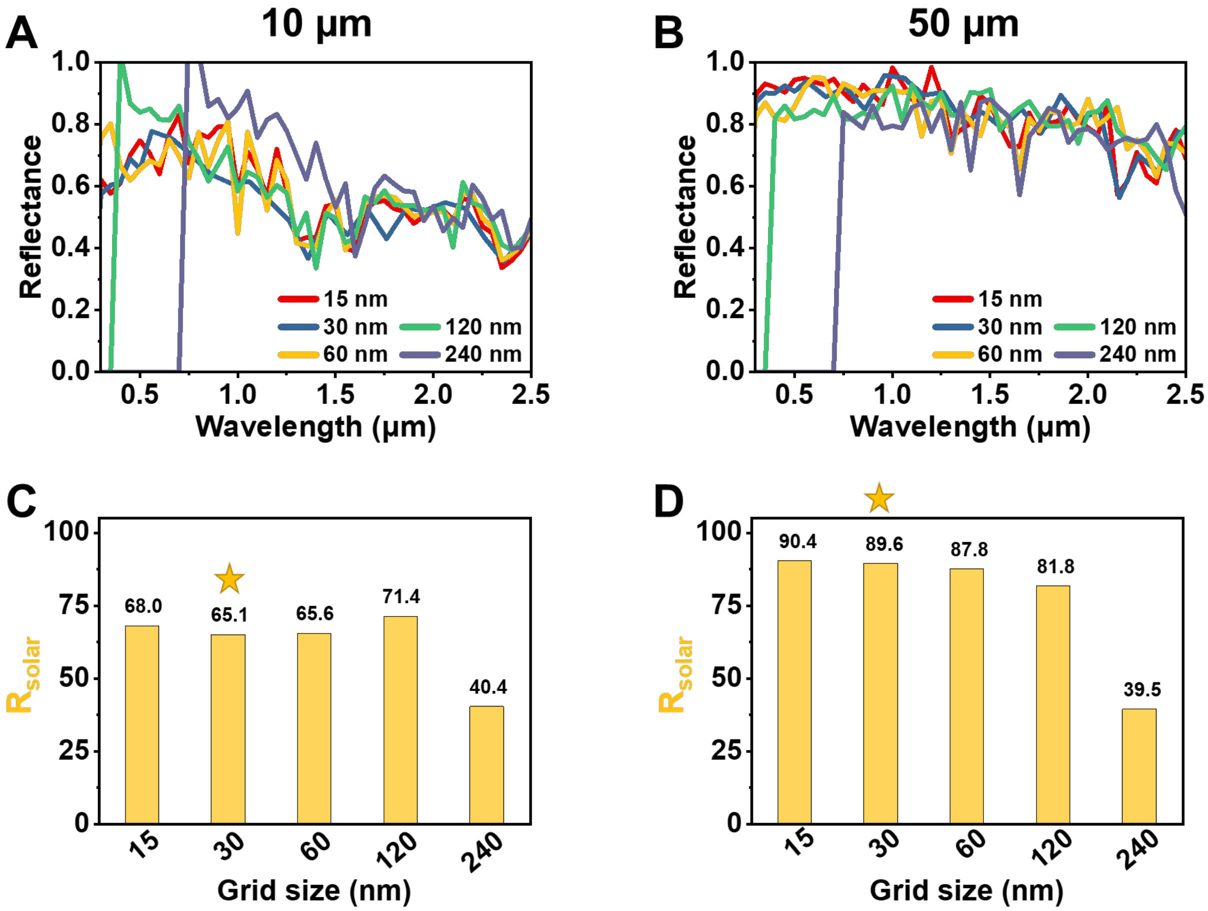

Figure S6. Calculated (A–B) solar reflectance spectra and (C–D) corresponding R_{solar} values of ZANF nanofiber membranes with thicknesses of (A, C) 10 μm and (B, D) 50 μm at a fixed porosity of 90%. The grid size for all FDTD simulations ranged from 15 nm to 240 nm.

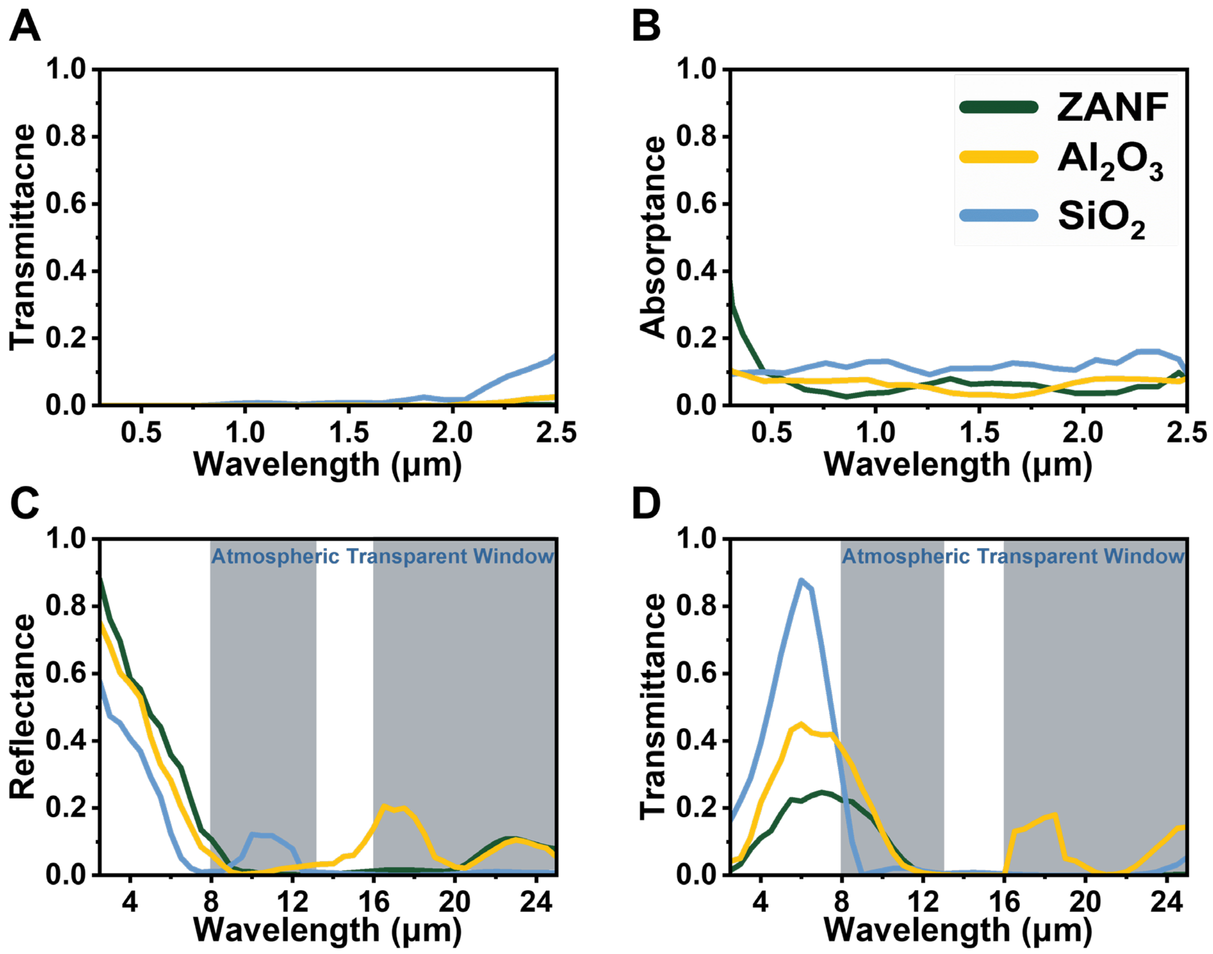

Figure S7. Calculated (A) solar transmittance, (B) solar absorptance, (C) MIR reflectance, and (D) MIR transmittance spectra of ZANF, Al_2O_3, and SiO_2 nanofibers. The thickness of the membranes was fixed at 500 μm with a porosity of 90%.

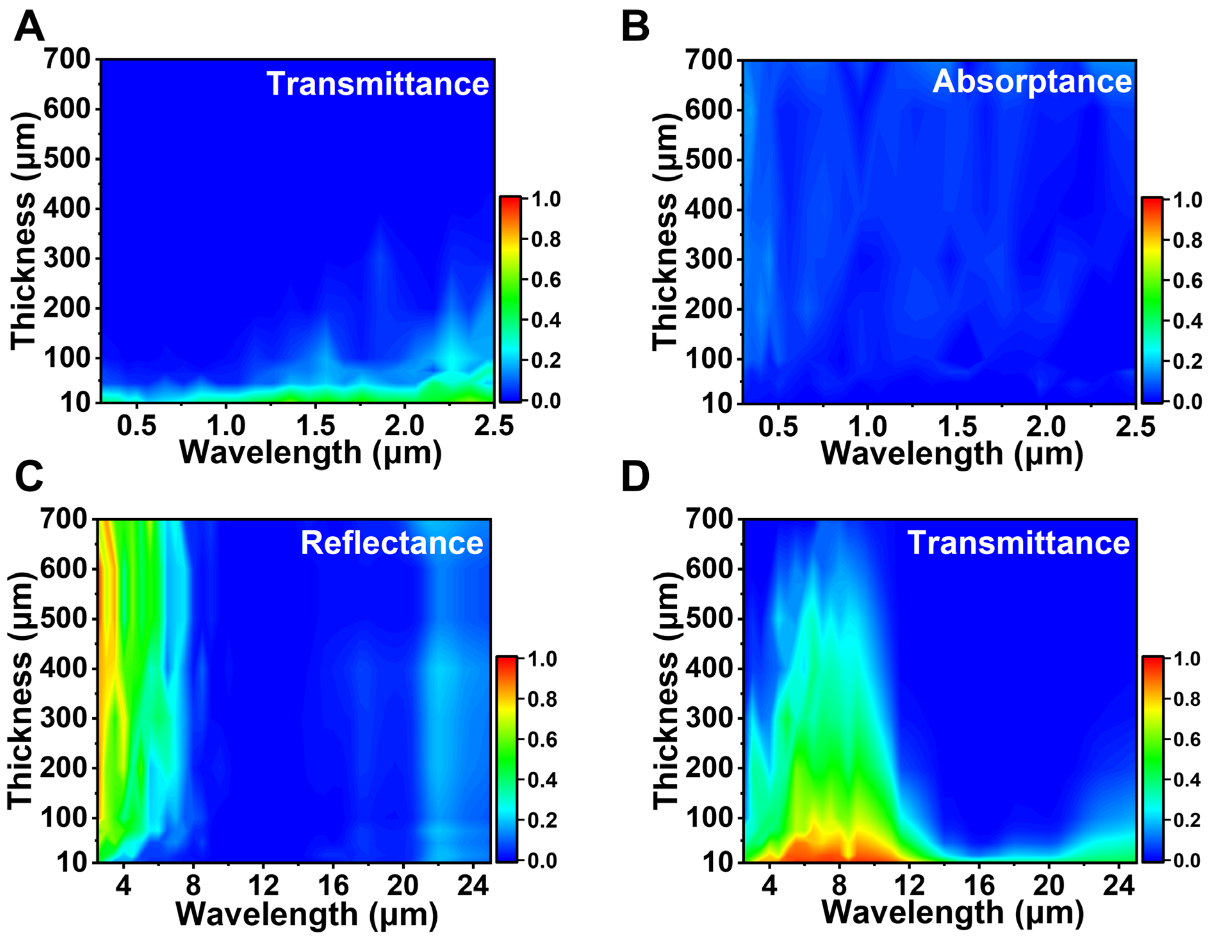

Figure S8. Calculated (A) solar transmittance, (B) solar absorptance, (C) MIR reflectance, and (D) MIR transmittance spectra of ZANF membranes at various thicknesses (10 to 700 μm) and a fixed porosity of 90%.

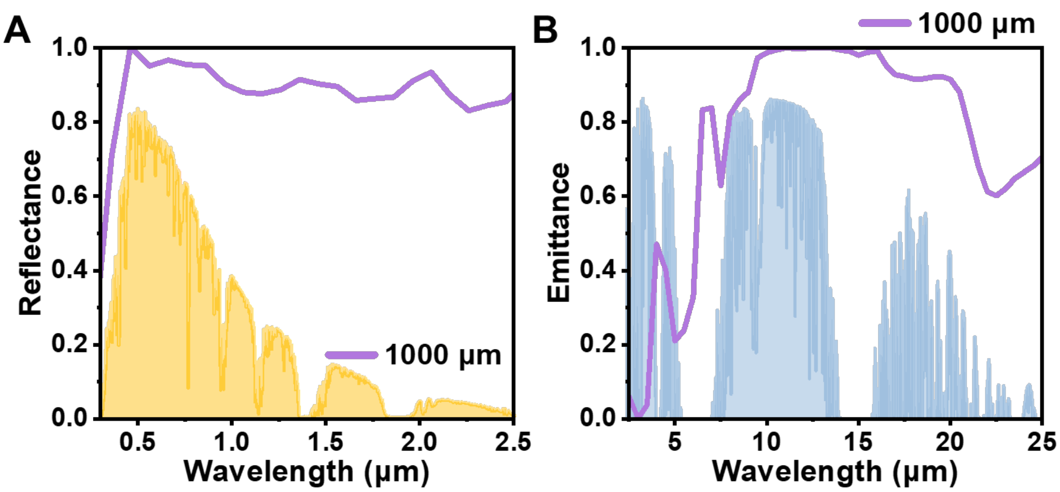

Figure S9. Calculated (A) solar reflectance and (B) MIR emittance spectra of a ZANF membrane with a thickness of 1000 μm and a porosity of 90%.

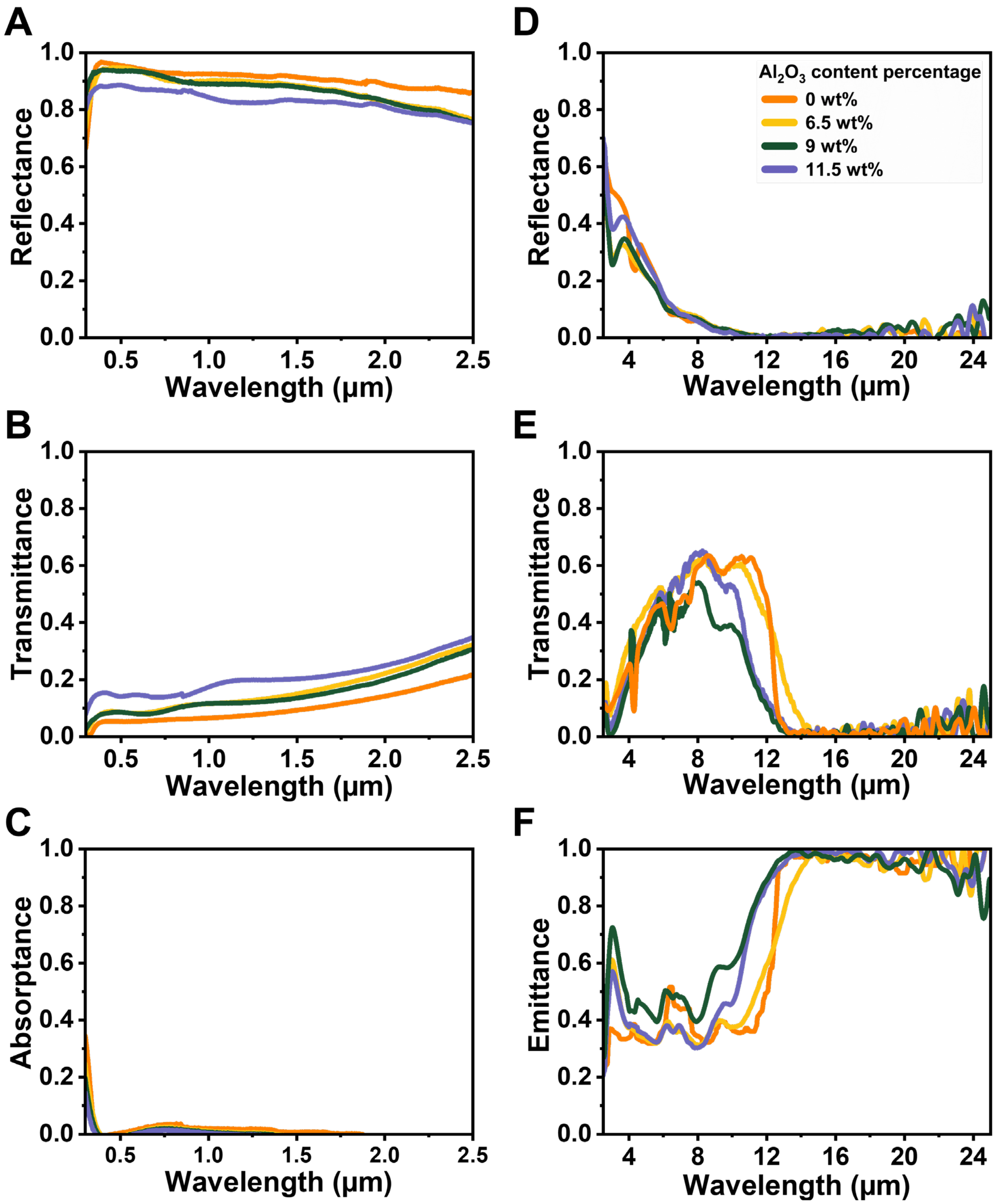

Figure S10. (A, D) Reflectance, (B, E) transmittance, and (C, F) absorptance spectra of ZANF membranes with different Al_2O_3 weight ratios cross the (A–C) solar and (D–F) MIR bands. The thickness of nanofibrous samples was fixed at 50 μm.

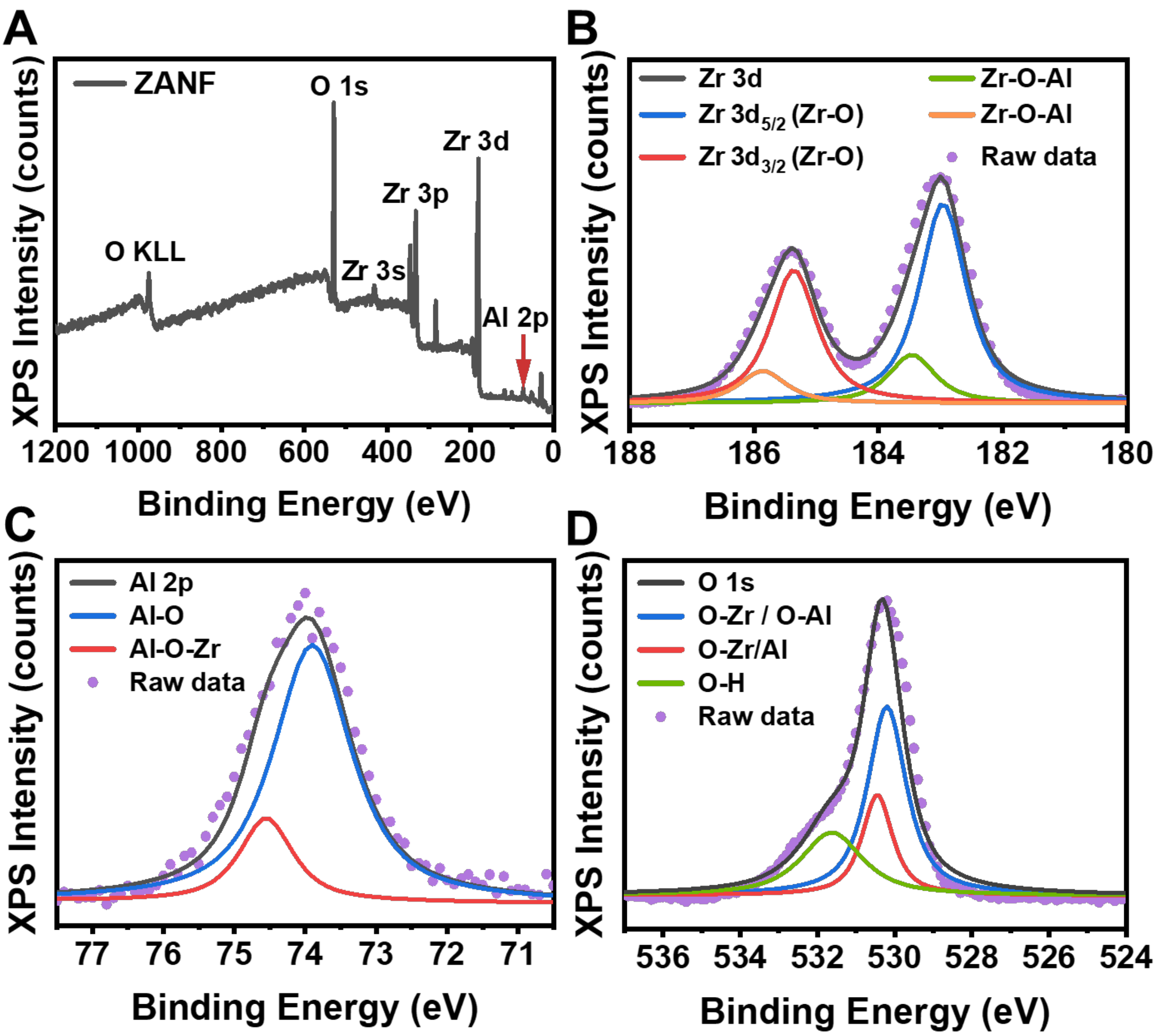

Figure S11. (A) XPS survey spectrum of a ZANF membrane. (B–D) High-resolution (B) Zr 3d, (C) Al 2p, and (D) O 1s spectra.

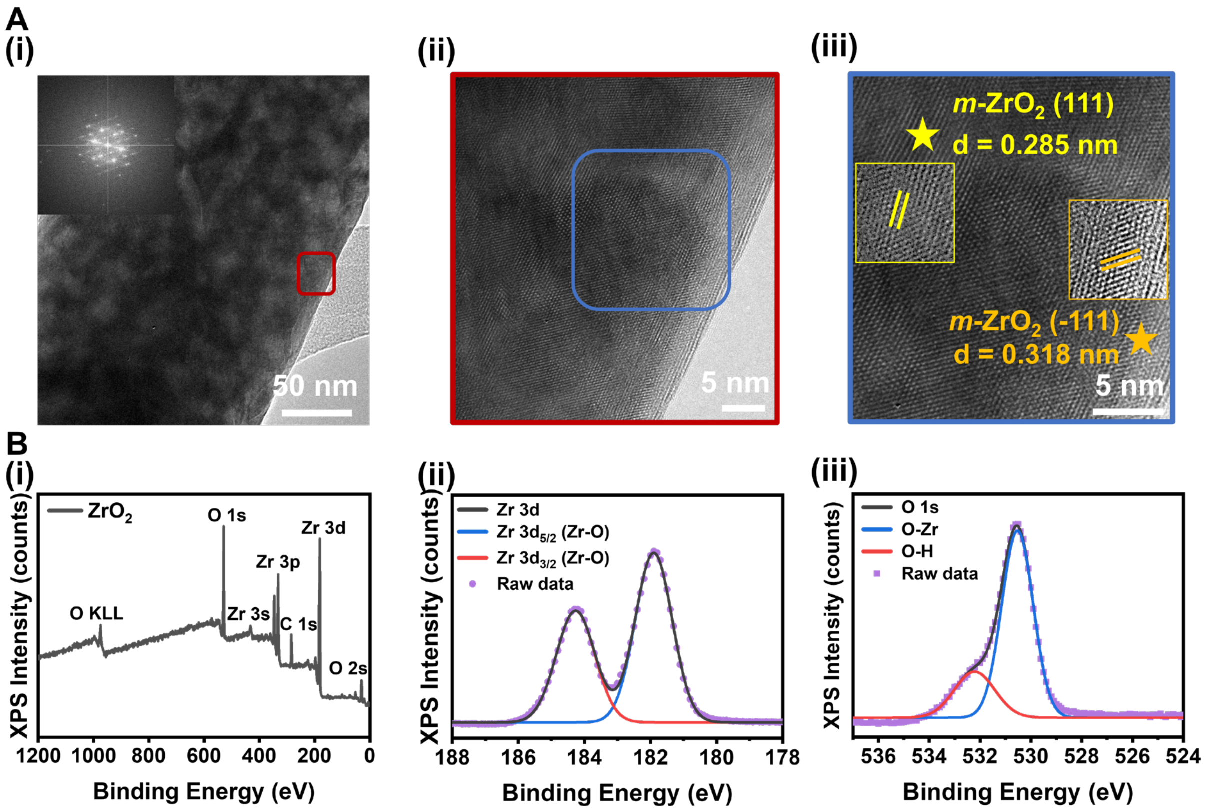

Figure S12. (A) HR-TEM images of a single ZrO_2 nanofiber. (i) Randomly arranged crystalline structures. (ii) Magnified view of the region outlined in red in (i). Areas outlined by white dash are the amorphous regions. (iii) Magnified view of the region outlined in blue in (ii), where the *m*-ZrO_2 crystallite planes of (111) and (-111) are distinguished by their lattice fringes. (B) XPS analysis of ZrO_2: (i) survey, (ii) high-resolution Zr 3d, and (iii) high-resolution O 1s spectra.

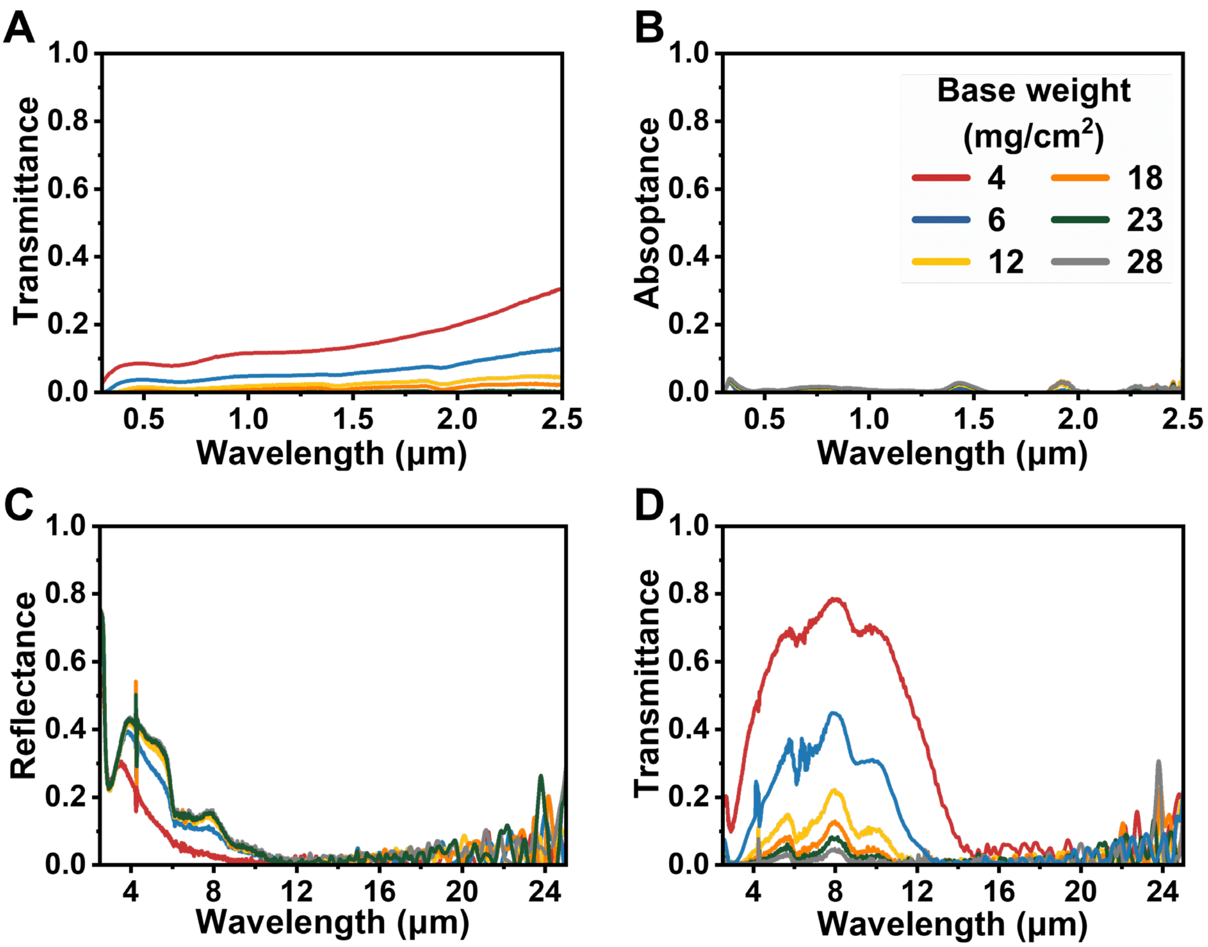

Figure S13. (A) Solar transmittance, (B) solar absorptance, (C) MIR reflectance, and (D) MIR transmittance spectra of sh-ZANF with base weights varying from 4 to 28 mg/cm^2.

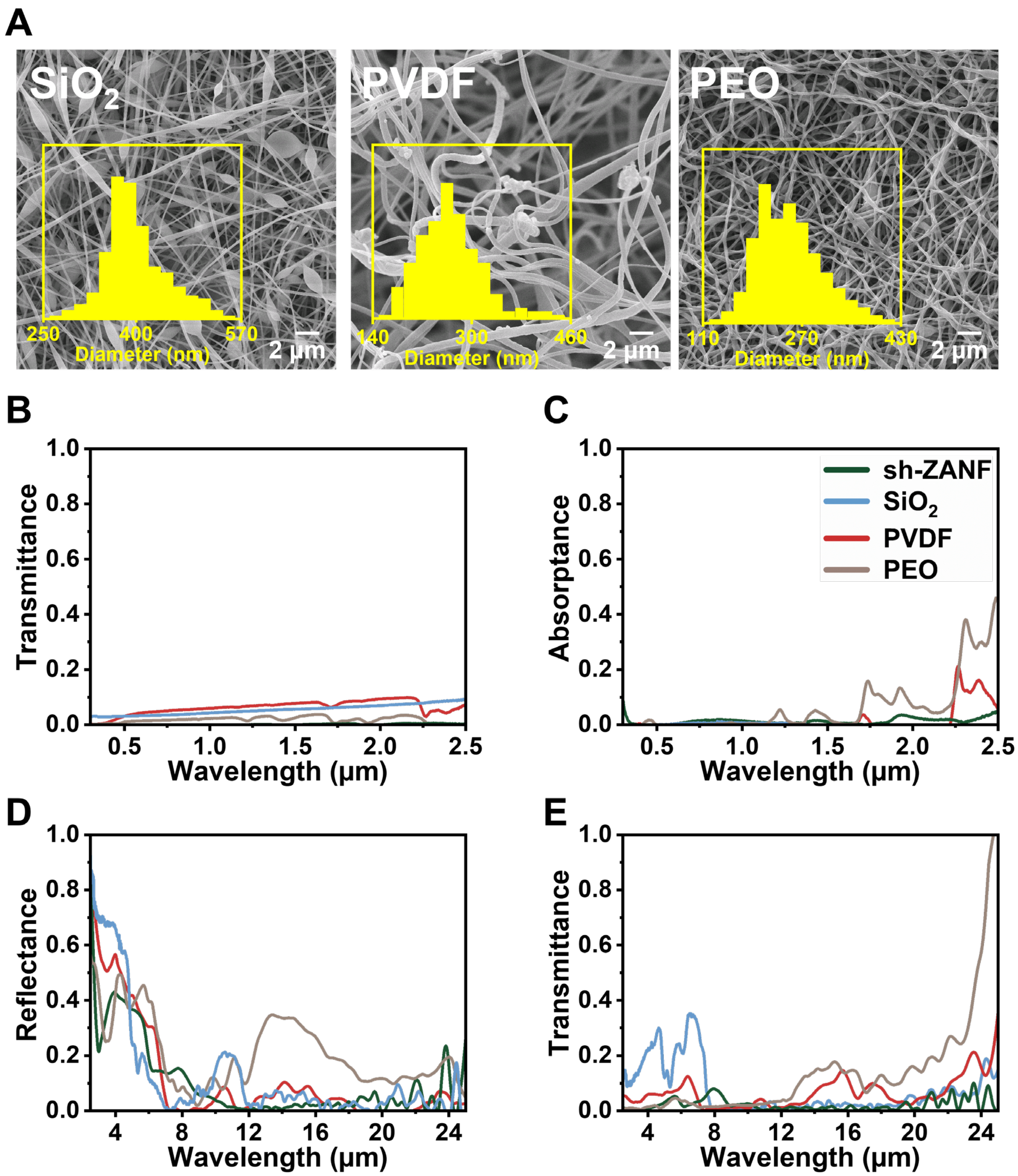

Figure S14. (A) SEM images and diameter distributions of SiO_2, PVDF, and PEO nanofibers. (B) Solar transmittance, (C) solar absorptance, (D) MIR reflectance, and (E) MIR transmittance spectra of sh-ZANF, SiO_2, PVDF, and PEO nanofibrous membranes.

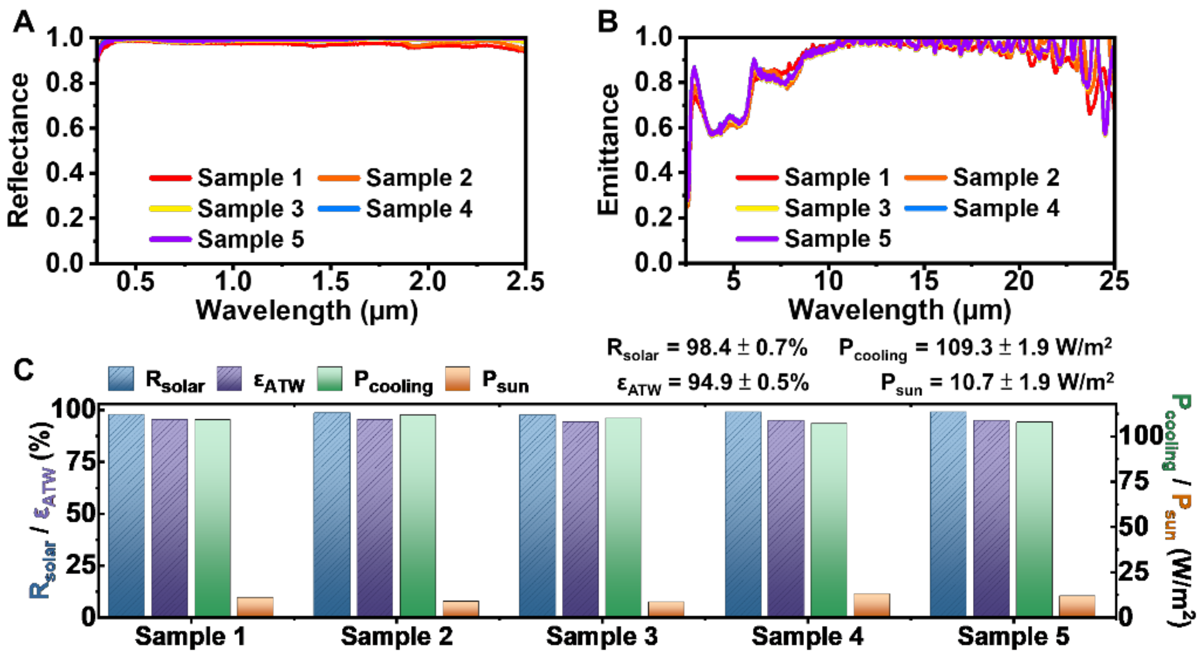

Figure S15. (A) Solar reflectance, (B) MIR emittance spectra and (C) calculated R_{solar}, ε_{ATW}, $P_{cooling}$, and P_{sun} values of sh-ZANF (n = 5).

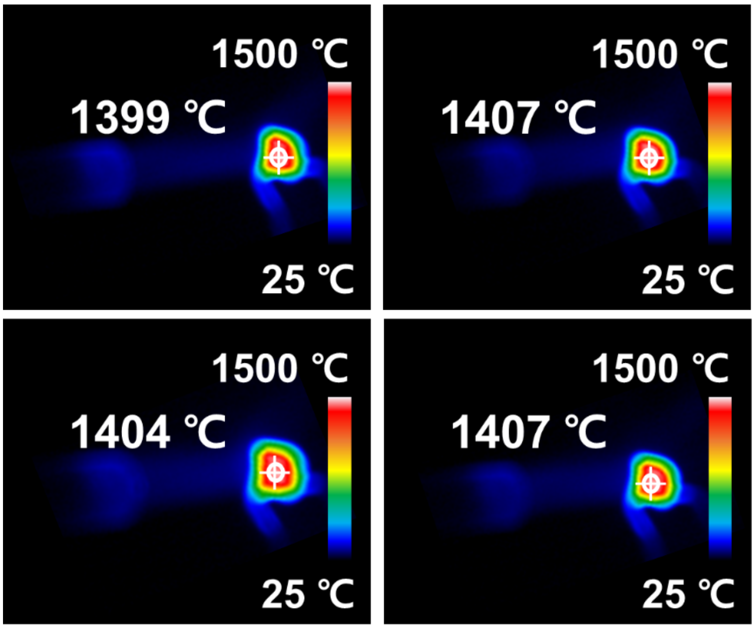

Figure S16. Representative thermal infrared images of sh-ZANF exposed to blowtorch flame (n = 4).

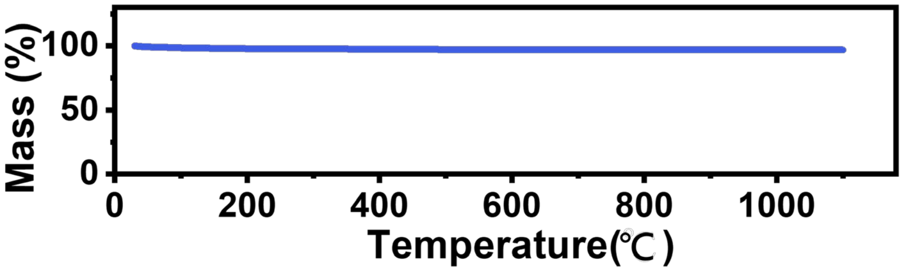

Figure S17. TGA analysis of sh-ZANF from 30 ℃ to 1100 ℃.

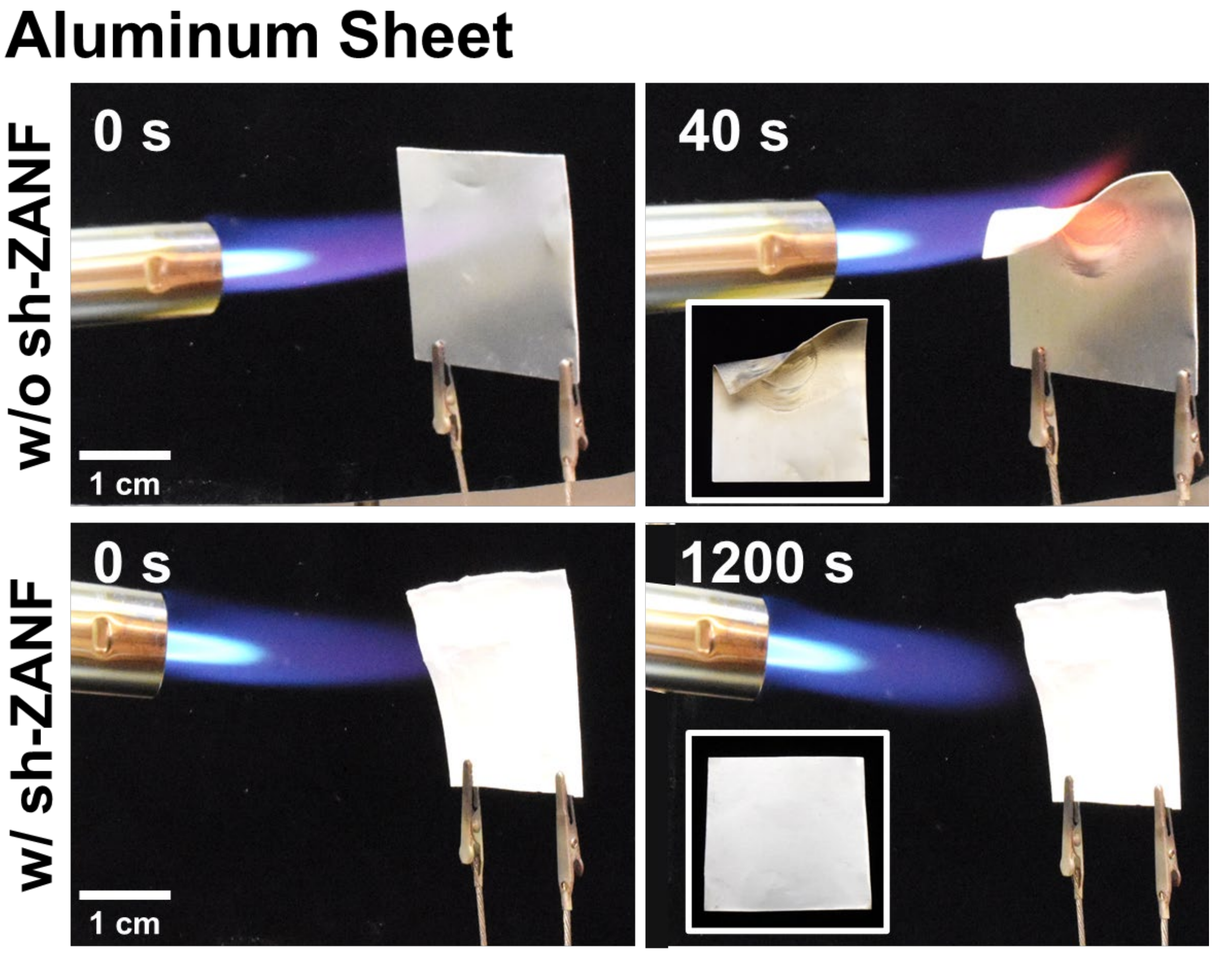

Figure S18. Photographs of aluminum sheets covered without/with sh-ZANF during the combustion test. Insets: the corresponding photographs after burning.

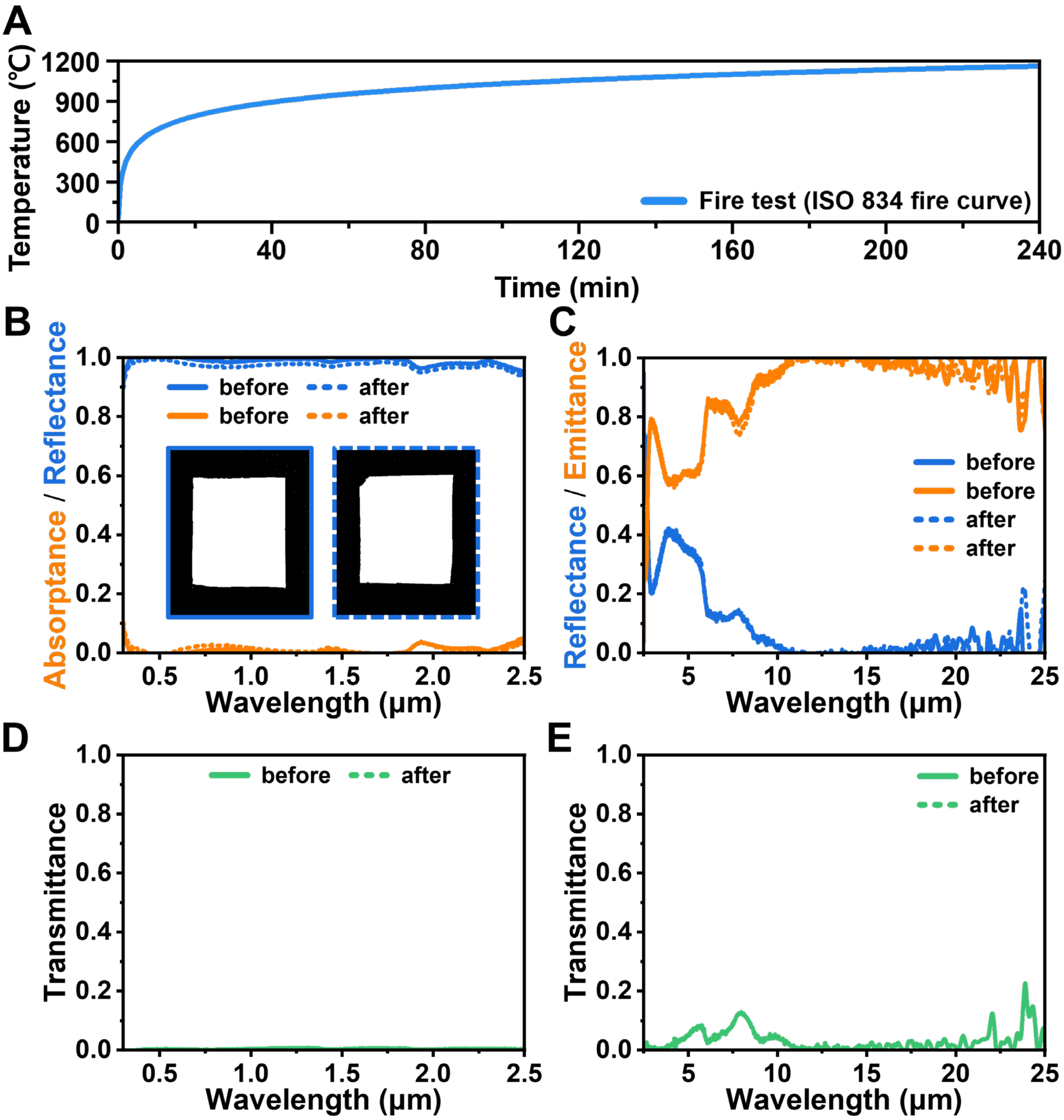

Figure S19. (A) Furnace temperature versus time curve according to the ISO 834 standard. (B–E) Solar and MIR spectra of sh-ZANF before and after the abovementioned fire test.

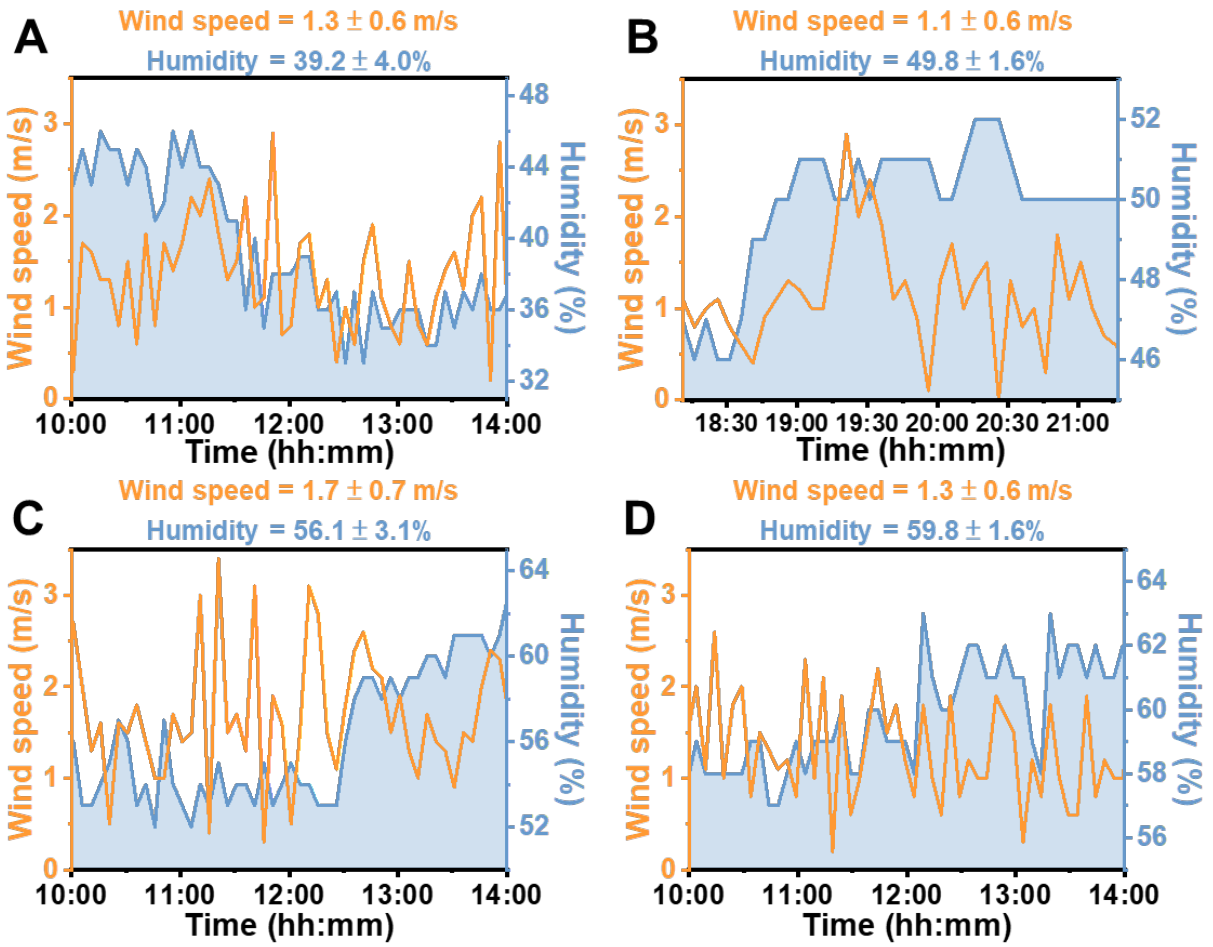

Figure S20. Wind speed and relative humidity during the thermal measurements for (A, B) sh-ZANF in (A) daytime and (B) nighttime, and (C) the building/automobile models and (D) handheld cameras.

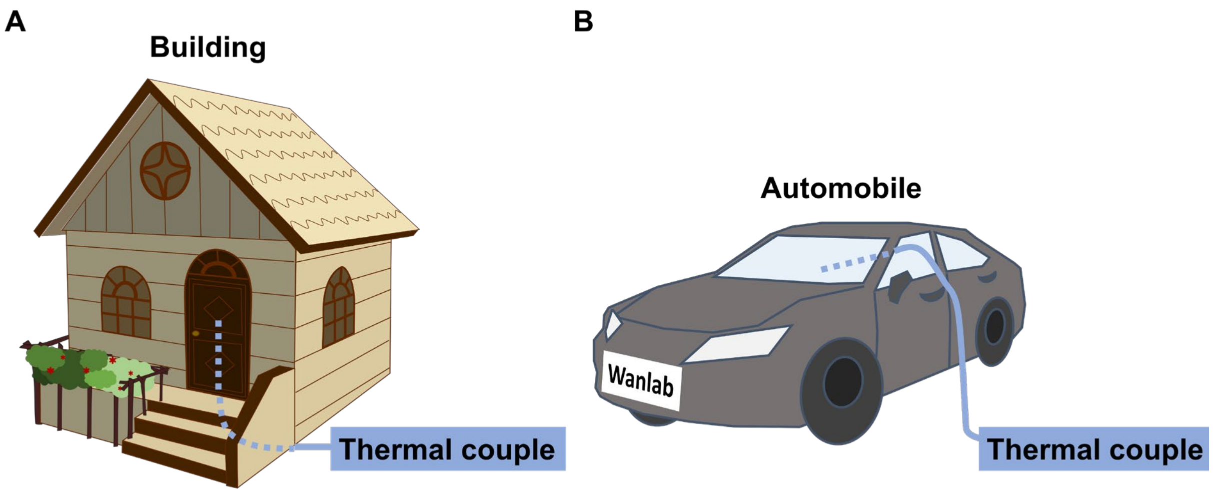

Figure S21. (A–B) Schematic representation of the points of interior temperature measurement in the (A) building and (B) automobile models.

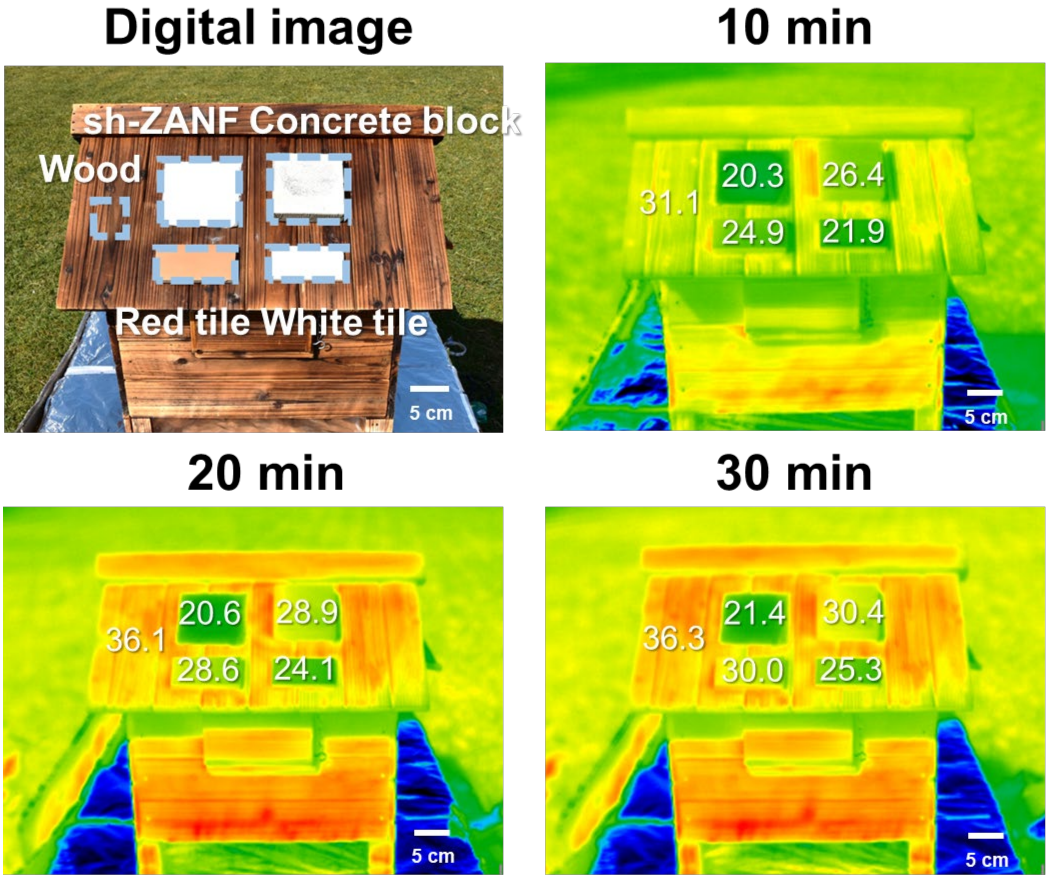

Figure S22. Sunlight exposure tests on a wooden cabin covered with sh-ZANF and various real construction materials for durations of 10, 20, and 30 minutes.

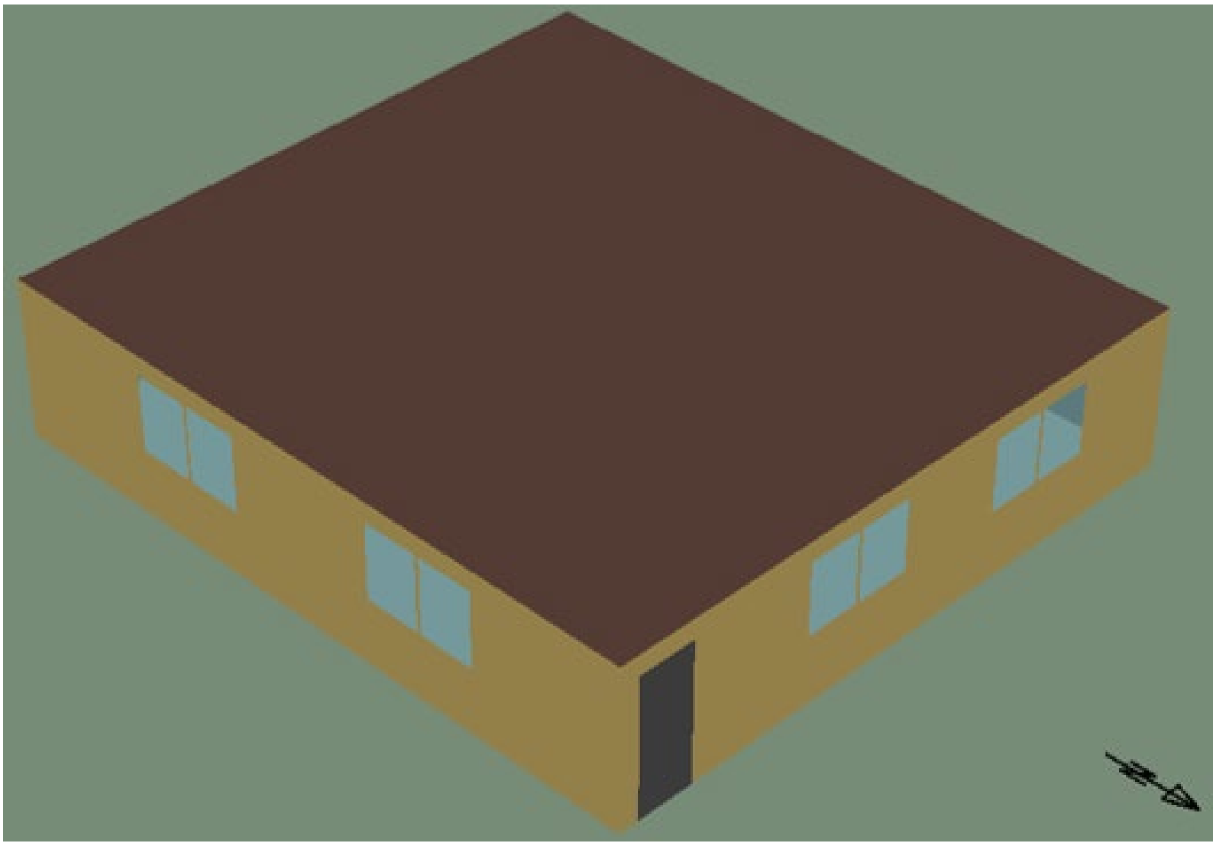

Figure S23. Schematic representation of a residential house with an area of 104 m^2, 8 windows (total area: 15 m^2), and 1 door (2 m^2). Building energy saving for this house was compared between two roof materials (sh-ZANF and dark metal). All-year weather data were obtained from EnergyPlus official website.

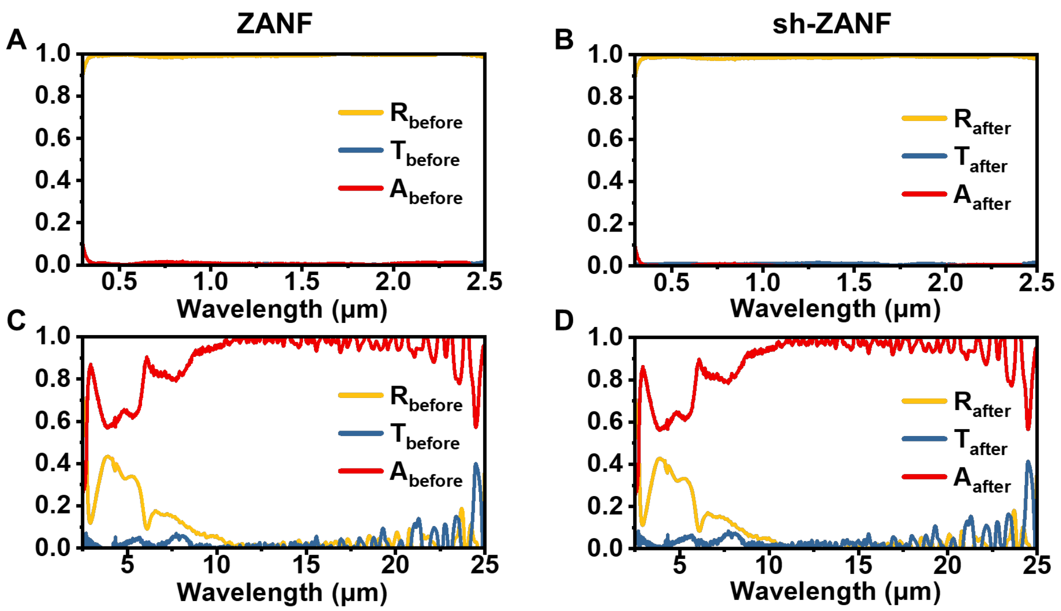

Figure S24. Reflectance, transmittance, and absorptance spectra of ZANF (A, C) before and (B, D) after hydrophobic treatment cross the (A, B) solar and (C, D) MIR bands.

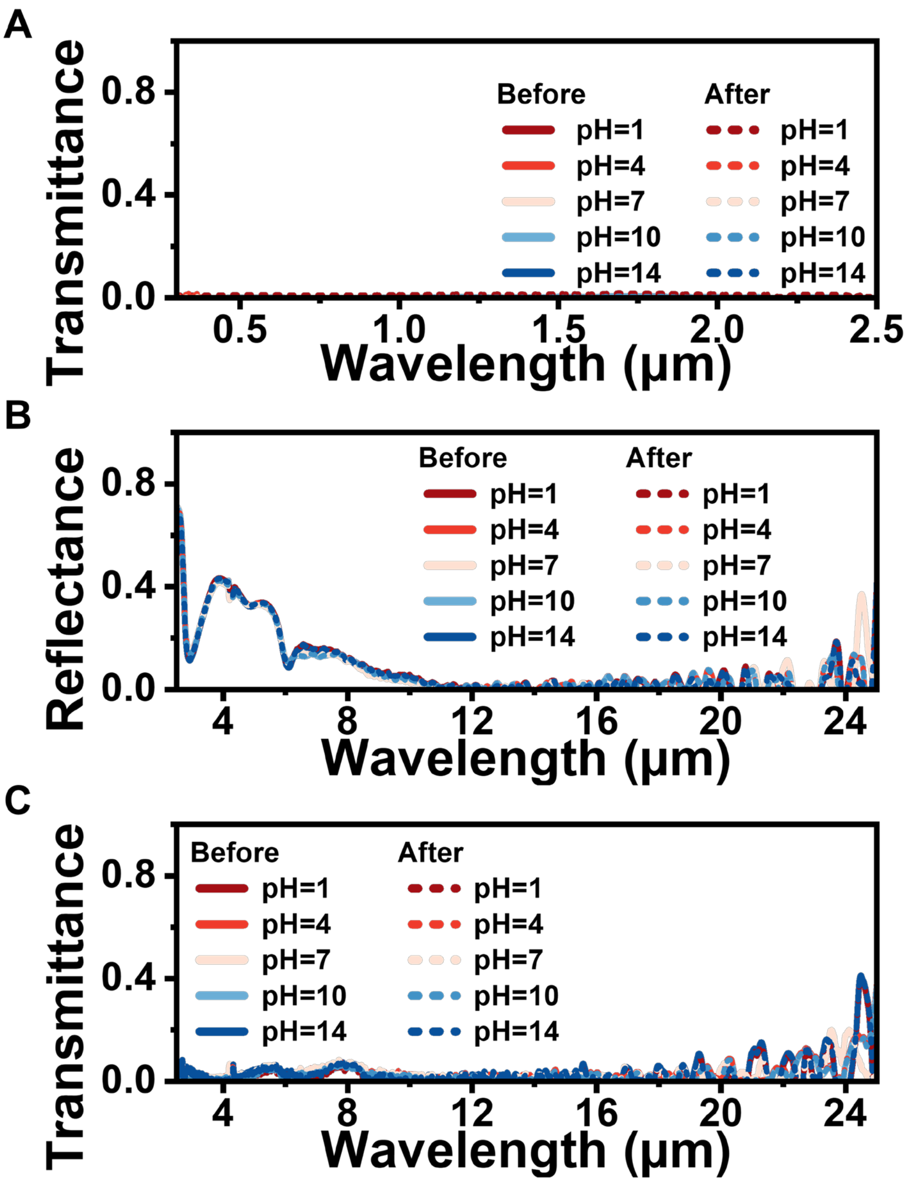

Figure S25. (A) Solar transmittance, (B) MIR reflectance, and (C) MIR transmittance spectra of sh-ZANF before and after immersion in solutions at various pH for 1 week.

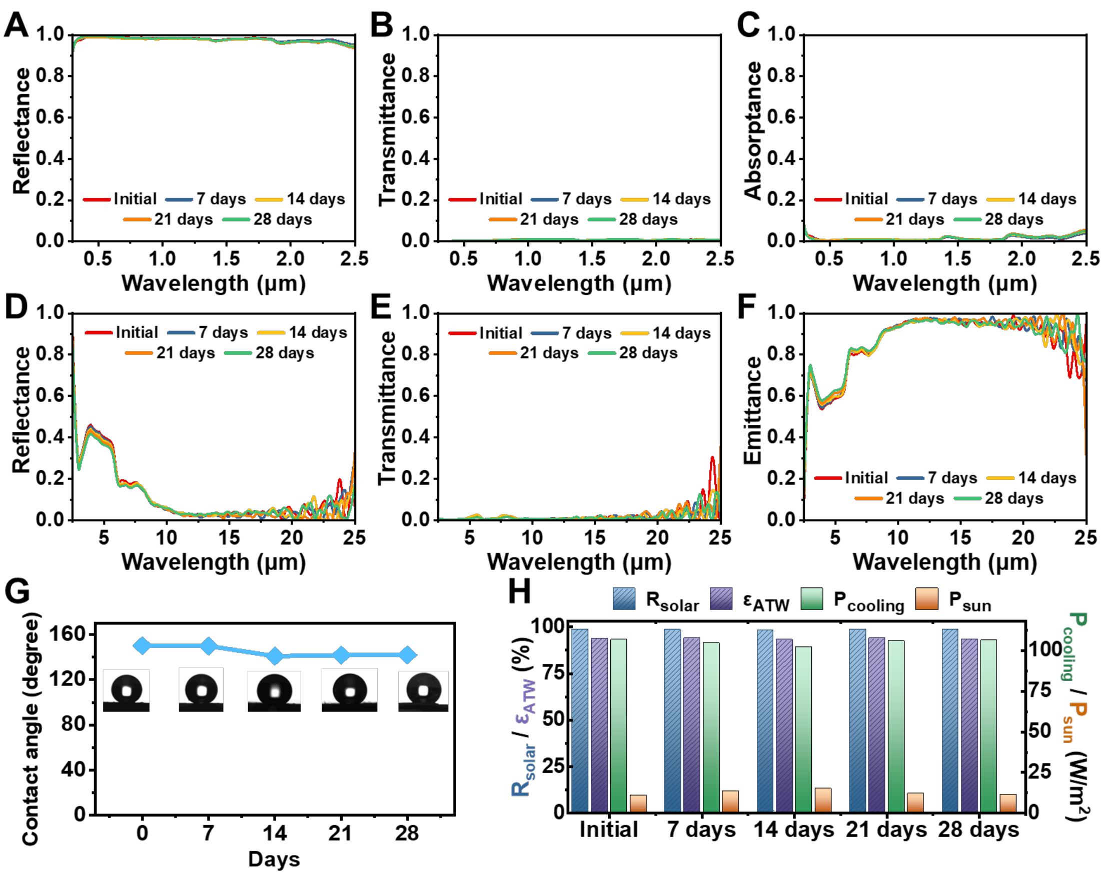

Figure S26. (A) Solar reflectance, (B) solar transmittance, and (C) solar absorptance spectra of sh-ZANF before and after the outdoor exposure tests. (D) MIR reflectance, (E) MIR transmittance and (F) MIR emittance spectra of sh-ZANF before and after the outdoor exposure tests. (G) Contact angles of sh-ZANF before and after the outdoor exposure tests. (H) Values of R_{solar}, ε_{ATW}, $P_{cooling}$, and P_{sun} of sh-ZANF before and after the outdoor exposure tests.

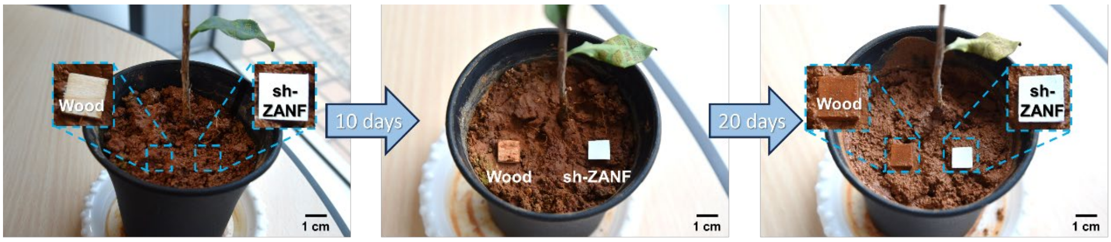

Figure S27. Soil burial test. After 30 days of burial, the sh-ZANF surface remained clean, while soil deeply infiltrated and saturated the wood.

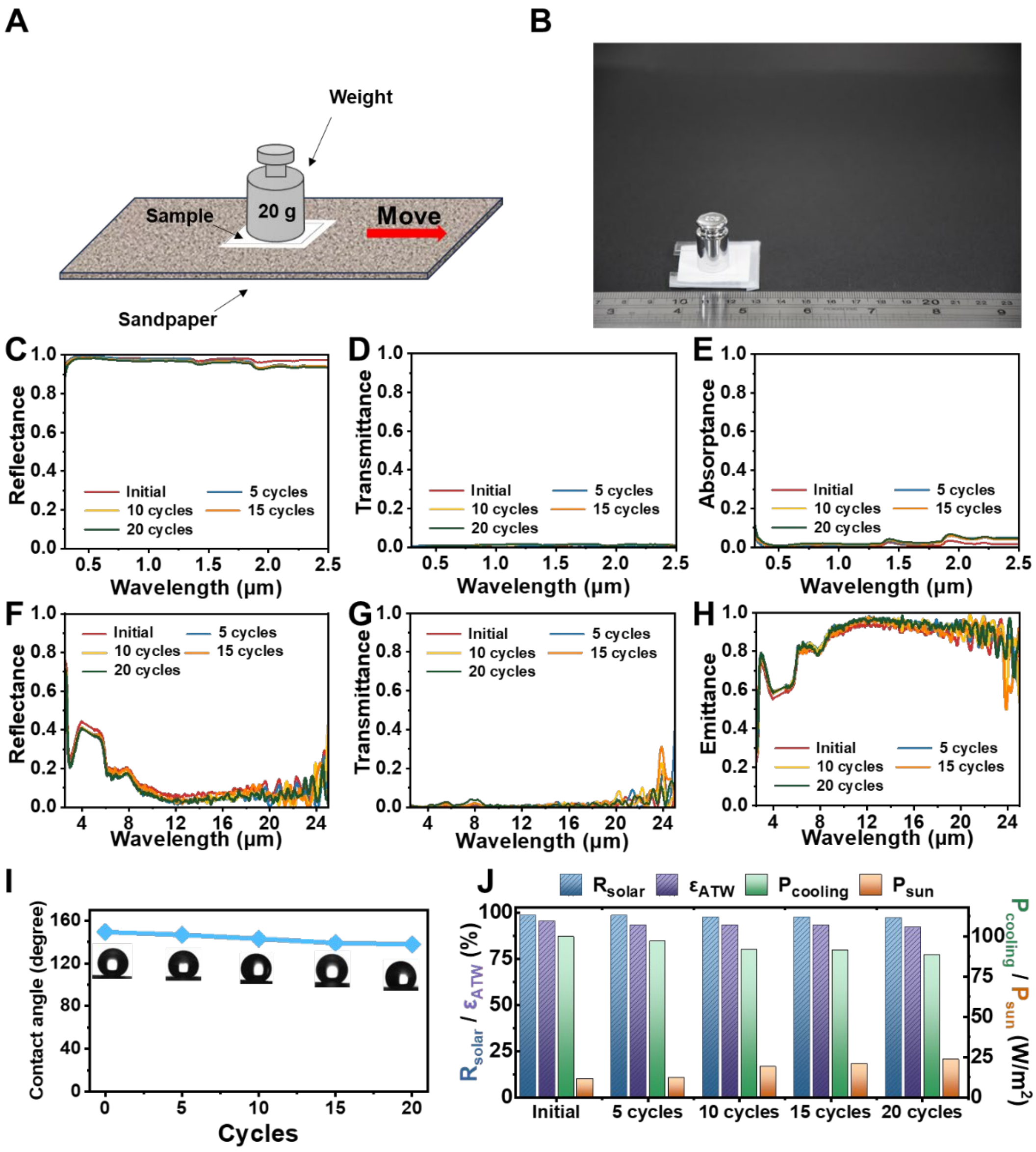

Figure S28. Abrasion tests. (A) Side view of the schematic setup for the abrasion tests. (B) A 20 g weight was placed on the sh-ZANF, which was positioned face-down on P400 sandpaper. The sample was moved 10 cm along a ruler, rotated 90°, and moved another 10 cm to complete one cycle. A total of 20 cycles were performed. (C) Solar reflectance, (D) solar transmittance, and (E) solar absorptance spectra of sh-ZANF before and after the abrasion tests. (F) MIR reflectance, (G) MIR transmittance and (H) MIR emittance spectra of sh-ZANF before and after the abrasion tests. (I) Contact angles of sh-ZANF before and after the abrasion tests. (J) Values of R_{solar}, ε_{ATW}, $P_{cooling}$, and P_{sun} of sh-ZANF before and after the abrasion tests.

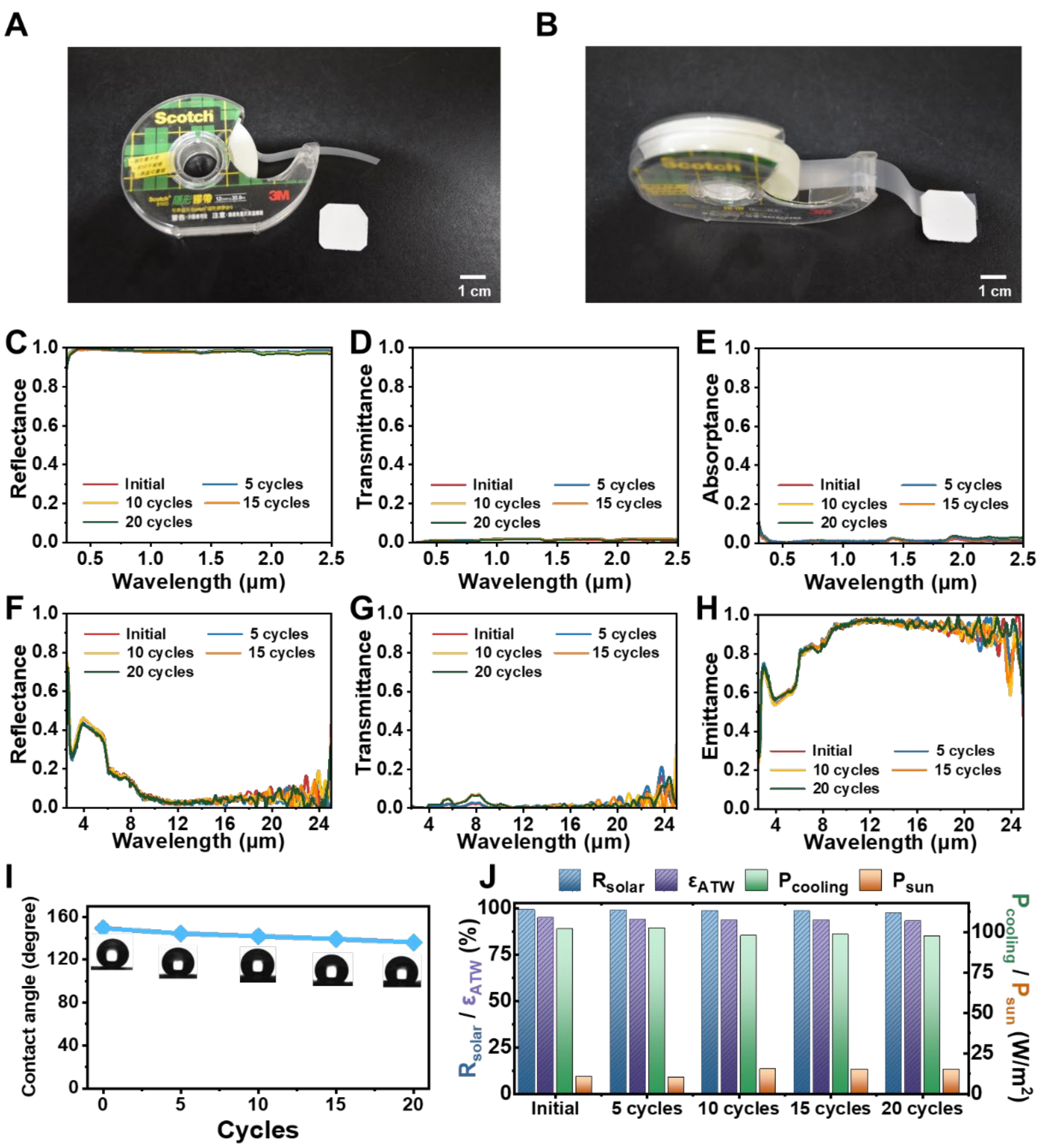

Figure S29. Tape peel-off tests. (A) Photographs showing the tape peel-off test performed on sh-ZANF using 3M Scotch Invisible Tape (model 810D, 12 mm × 32.9 m). (B) The tape was adhered to the material surface and then peeled off to evaluate adhesion properties. (C) Solar reflectance, (D) solar transmittance, and (E) solar absorptance spectra of sh-ZANF before and after the peel-off tests. (F) MIR reflectance, (G) MIR transmittance and (H) MIR emittance spectra of sh-ZANF before and after the peel-off tests. (I) Contact angles of sh-ZANF before and after the peel-off tests. (J) Values of R_{solar}, ε_{ATW}, $P_{cooling}$, and P_{sun} of sh-ZANF before and after the peel-off tests.

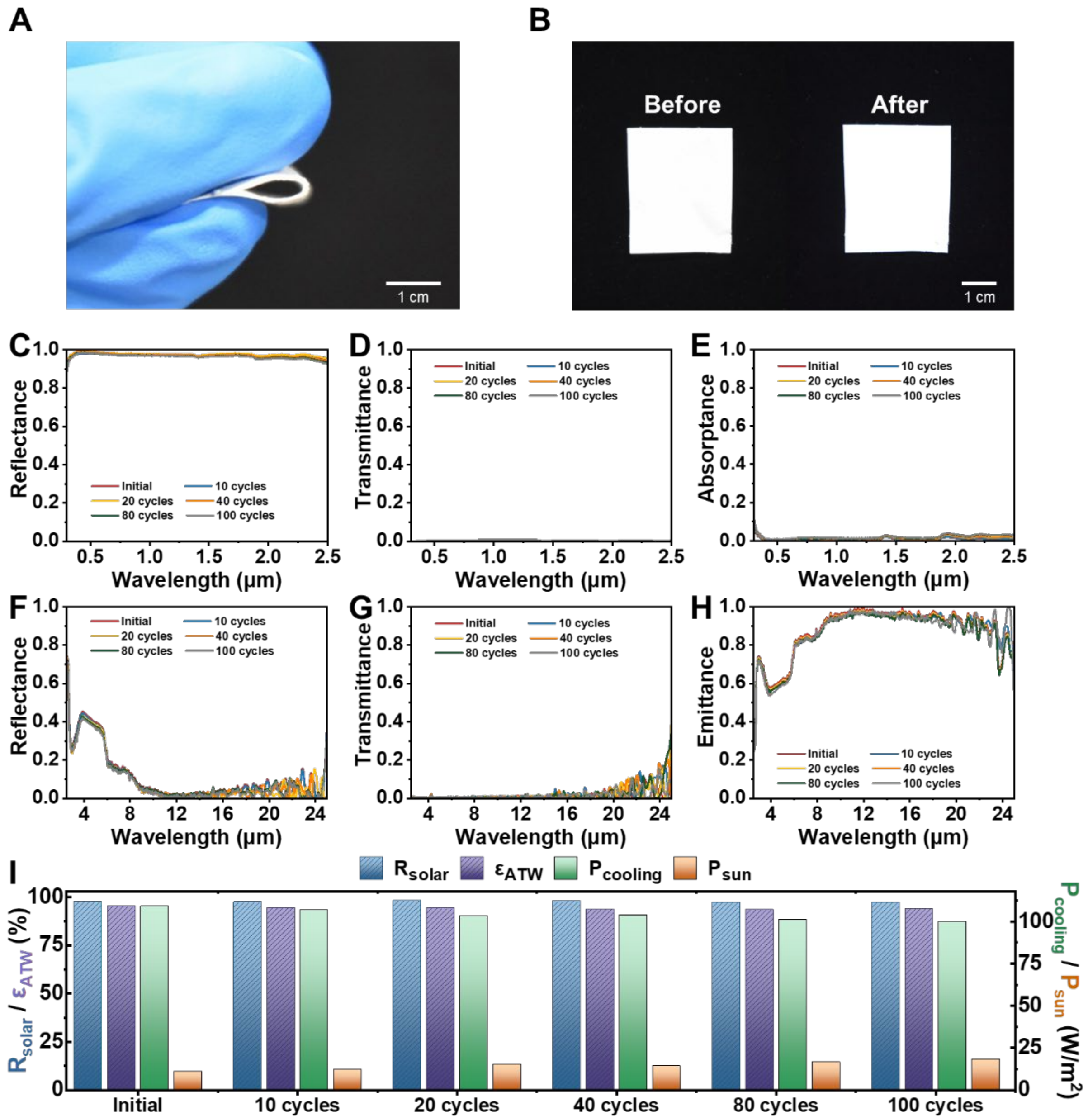

Figure S30. Folding cyclic tests. (A) Photographs demonstrating the folding test procedure on sh-ZANF with each fold maintaining a 180° angle. (B) Visual comparison of sh-ZANF appearance before and after folding tests. (C) Solar reflectance, (D) solar transmittance, and (E) solar absorptance spectra of sh-ZANF before and after the folding tests. (F) MIR reflectance, (G) MIR transmittance and (H) MIR emittance spectra of sh-ZANF before and after the folding tests. (I) Values of R_{solar}, ε_{ATW}, $P_{cooling}$, and P_{sun} of sh-ZANF before and after the folding tests.

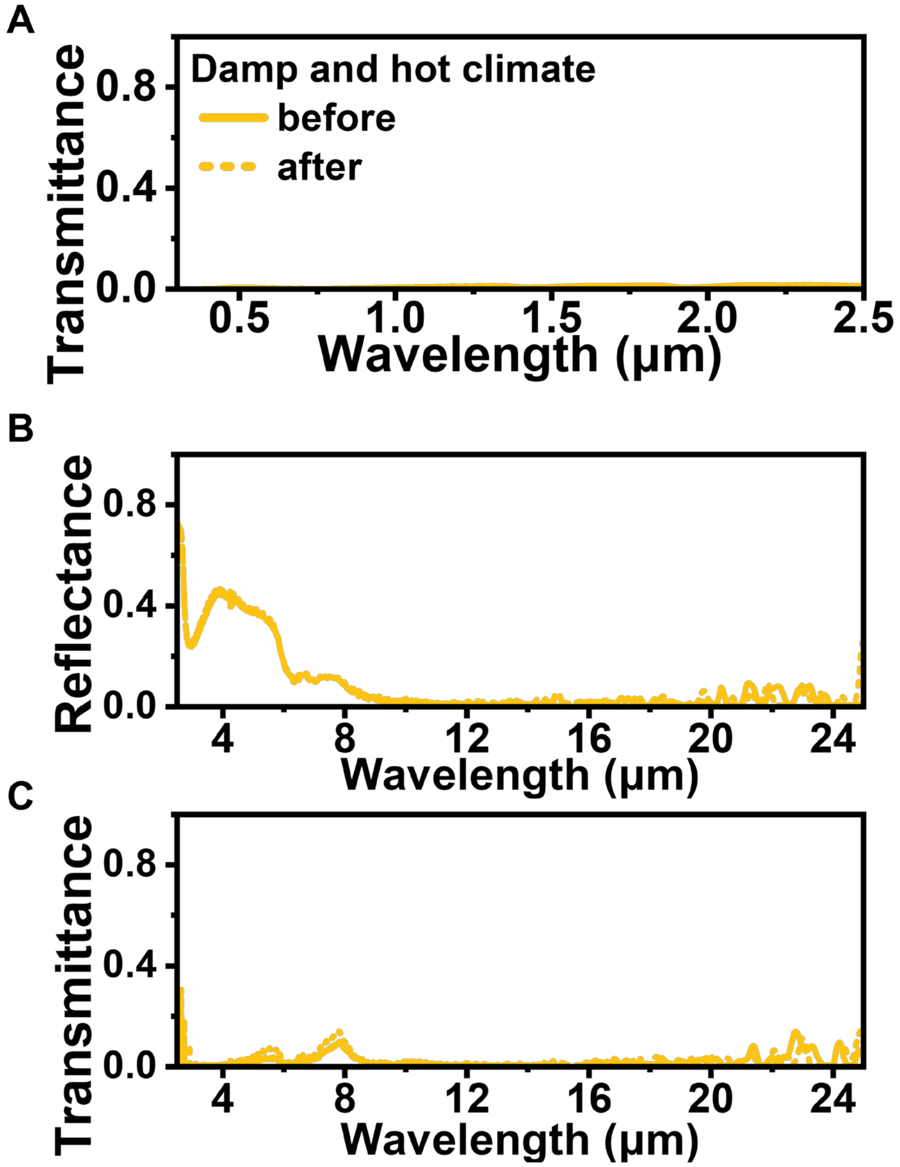

Figure S31. (A) Solar transmittance, (B) MIR reflectance, and (C) MIR transmittance spectra of sh-ZANF before and after damp heat test.

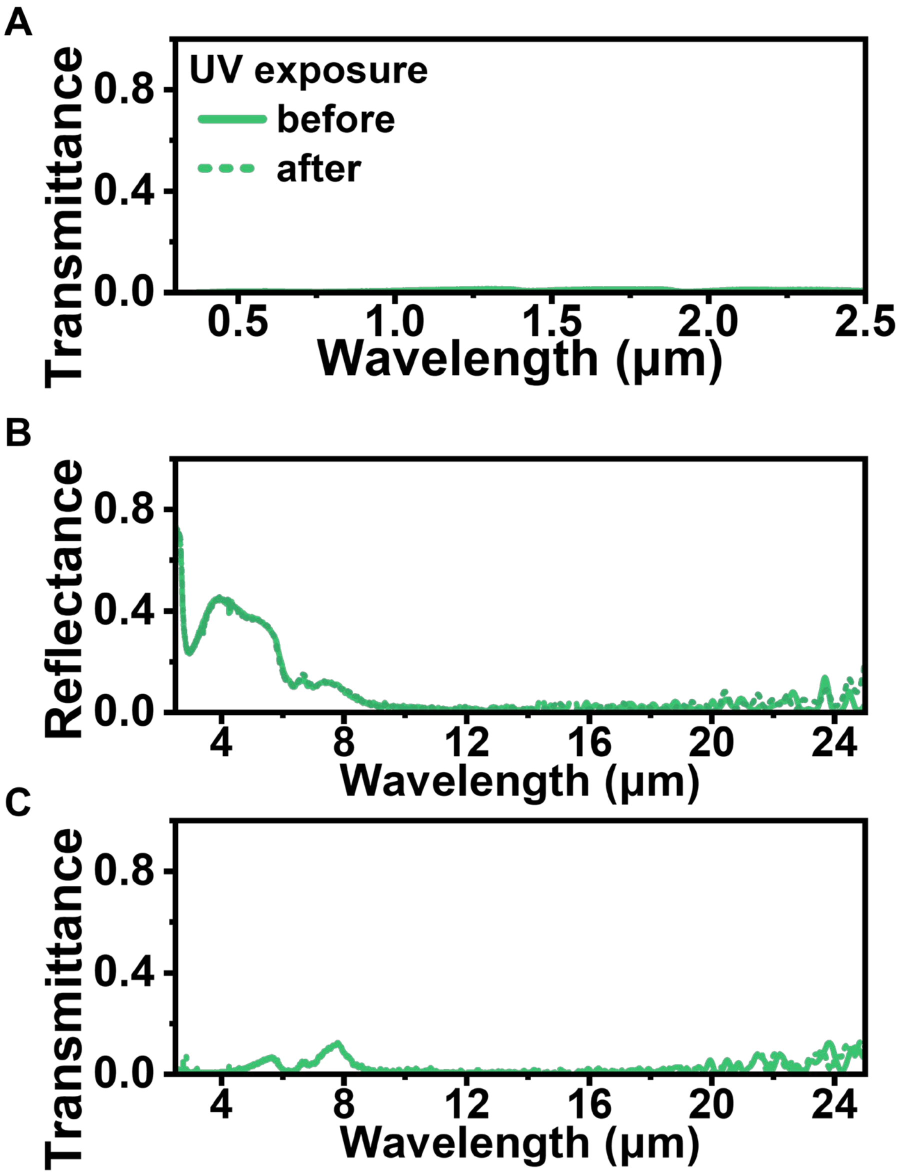

Figure S32. (A) Solar transmittance, (B) MIR reflectance, and (C) MIR transmittance spectra of sh-ZANF before and after UV preconditioning test.

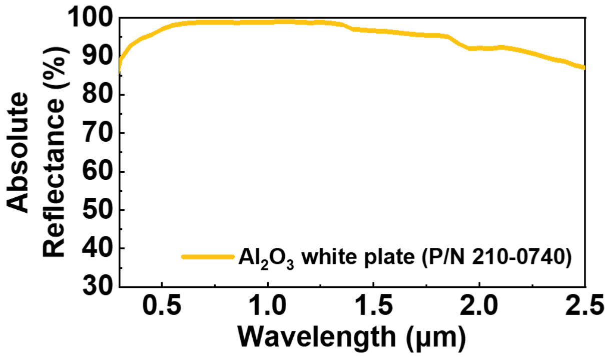

Figure S33. Absolute reflectance spectrum of an Al_2O_3 white plate (P/N 210-0740, Hitachi) over a wavelength range of 0.3-2.5 μm.

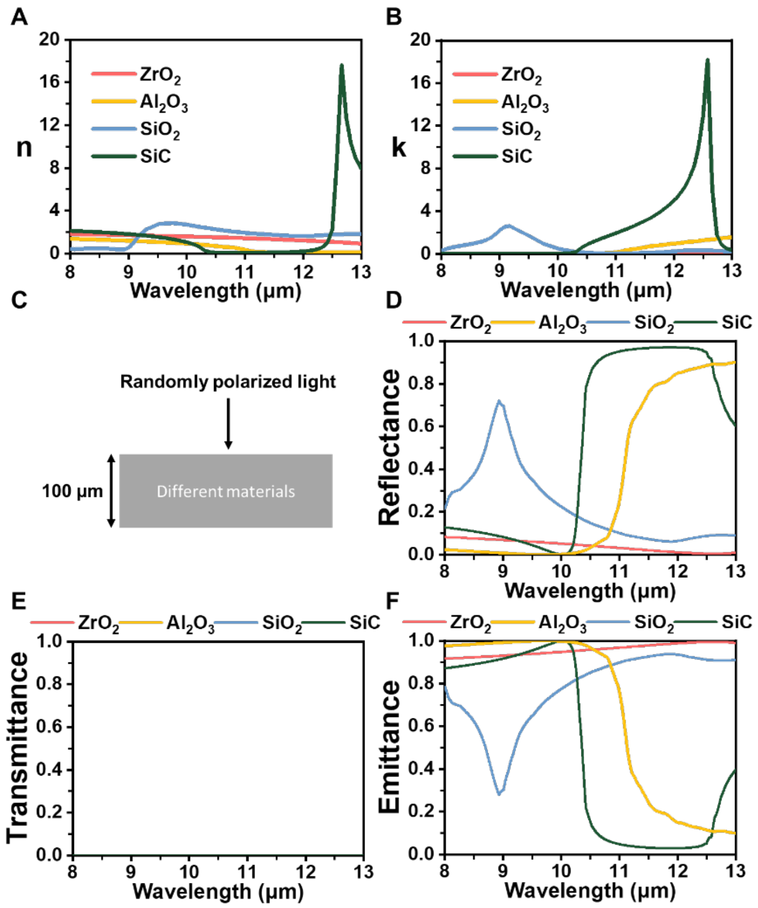

Figure S34. (A) refractive indices (*n*) and (B) extinction coefficients (*k*) of ZrO_2, Al_2O_3, SiO_2, and SiC in the atmospheric transparent window. (C) Schematic representation of the setup in Film Wizard optical simulation of 100 μm thin solid films of various ceramic materials. Calculated (D) reflectance, (E) transmittance, and (F) emittance spectra of ZrO_2, Al_2O_3, SiO_2, and SiC thin film with fixed thickness of 100 μm.

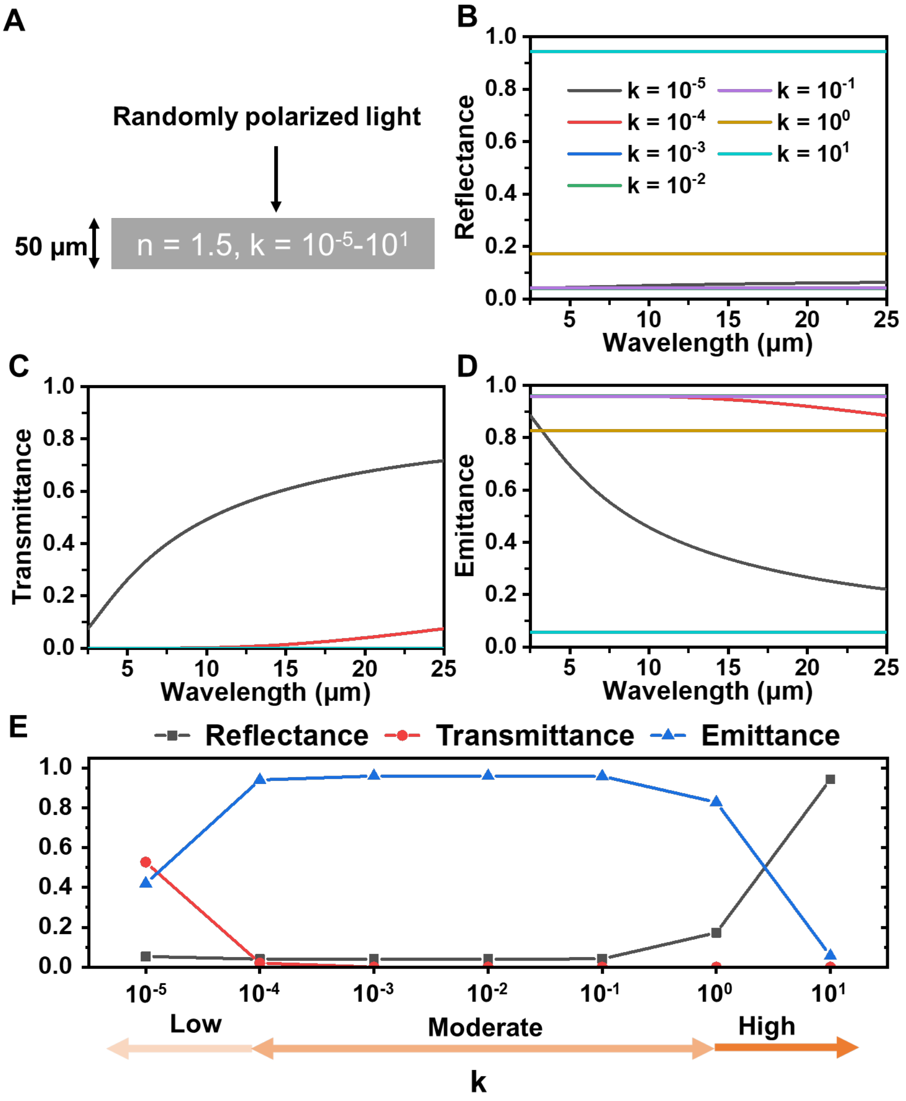

Figure S35. (A) Schematic representation of the setup in Film Wizard optical simulation of 50 μm thin films with varying *k* values while keeping *n* fixed at 1.5. Calculated (B) reflectance, (C) transmittance, (D) emittance spectra, and (E) average MIR optical properties of 50 μm thin films with varying *k* values (ranging from 10^{-5} to 10) while keeping *n* fixed at 1.5.

Table S1. Estimated material cost for producing 1g of sh-ZANF and 1g of Al_2O_3 nanofiber.

**Material**	**1g of sh-ZANF**			**1g of Al_2O_3 nanofibers**	
**Main ingredients**	$Zr(Ac)_4$	$AlCl_3$•$6H_2O$	AIP	$AlCl_3$•$6H_2O$	AIP
	USD $1.09	USD $0.53	USD $3.87	USD $6.22	USD $42.18
**Total cost**	USD $5.50			USD $48.40	

Table S2. Performance characteristics of durable PDRC materials in previous and current studies.

Material	$R_{solar}/\varepsilon_{ATW}$	Sub-ambient cooling	$P_{cooling}$ (calculated/ measured)	Flame resistance	UV anti-aging test	Anti-erosion test	Long-term test	Reference
PTFE MP/ cellulose MF	0.93/-	5 °C	-/104 W/m^2	-	30 d (3 mW/cm^2)	15 min/ in water	-	*ACS Appl. Mater. Interfaces* (2021)[5]
$K_2Ti_6O_{13}$/ PEO NF	0.94/0.91	8 °C	80 (303 K)/ 92 W/m^2	-	30 d under direct sunlight	-	30 d	*Adv. Mater.* (2022)[13]
TiO_2 NP/ PFOTS	0.93/0.97	3.6 °C	95 (303 K)/ 95 W/m^2	100 °C (1000 h)	1000 h (0.89 W/m^2)	-	6 months	*Nat. Commun.* (2022)[14]
ZrO_2 NP/ PDMS CT	0.92/0.95	7.5 °C	113 W/m^2 (308 K)/-	80 °C (240 h)	120 h	-	-	*Inorg. Chem. Commun.* (2023)[15]
ZnAl LDO MFR/kaolin CT	0.976/0.98	7 °C	100 W/m^2 (303 K)/-	-	6 h (1.4 kW/m^2)	-	-	*Chem. Eng. J.* (2023)[16]
Glass/Al_2O_3 porous CT	0.96/0.95	3 °C	60.1 W/m^2 (303 K)/-	1000 °C (10 s)	80 d (5 W/m^2)	-	3-y anti-soiled examination	*Science* (2023)[17]
SiO_2 NF	0.97/0.9	6 °C	109.36 W/m^2 (303 K)/ 112 W/m^2	1256 °C	180 d (60 mW/cm^2)	7 d pH=1, 3, 5, and 7	2 months	*Nano Today* (2023)[2]
BST@TPU membrane	0.972/0.932	10.3 °C	125.21 W/m^2 (303 K)/-	-	216 h (0.7 kW/m^2)	-	-	*Adv. Funct. Mater.* (2024)[18]
PVDF NF/silk NF/silk fabrics	0.965/0.971	5.1 °C	-	-	80 h (0.89 W/m^2)	-	-	*Adv. Funct. Mater.* (2024)[19]
Al_2O_3 NF	~0.96/~0.87	4.5 °C compared to concrete	-/75 W/m^2	-	30 d	-	-	*Adv. Funct. Mater.* (2025)[4]
sh-ZANF	0.977/0.956	10 °C	113.43 W/m^2 (303 K)/ 125 W/m^2	1407 °C (1200 s)	accumulated 15 kWh/m^2	7 d pH=1–14	25-y aging test 85 °C/RH85%	This work

Notes: MP = microparticles; MF = microfibers; NF = nanofibers; NP = nanoparticles; CT = coating; LDO = layered double oxides; MFR = microflowers; BST = strontium barium titanate; TPU = thermoplastic polyurethane

Table S3. Parameters used in the BEopt simulation.

U value	Window	0.38 Btu/hRft^2
	Door	0.2 Btu/hRft^2
Internal loads	Furnace	0.5 W/cfm
	Water heater	4.5 kW
	Lightning	751 kWh/year
	Refrigerator	434 kWh/year
	Dishwasher	111 kWh/year
	Clothes washer	42.9 kWh/year
	Plug load	1961 kWh/year
HVAC setpoints	Cooling	Morning: 85 °F<br>Evening: 76 °F
	Heating	63 °F
Weather files	Los Angeles	USA_CA_Los.Angeles.722950_TMY2.epw
	Phoenix	USA_AZ_Phoenix.722780_TMY2.epw
	New York	USA_NY_ New.York-Central.Park.725033_TMY3.epw
	Brasilia	BRA_Brasilia.833780_IWEC.epw
	Paris	FRA_Paris.Orly.071490_IWEC.epw
	Barcelona	ESP_Barcelona.081810_IWEC.epw
	Rome	ITA_Rome.162420_IWEC.epw
	Cape Town	ZAF_Cape.Town.688160_IWEC.epw
	Cairo	EGY_Cairo.623660_IWEC.epw
	Abu Dhabi	ARE_Abu.Dhabi.412170_IWEC.epw
	New Delhi	IND_New.Delhi.421820_ISHRAE.epw
	Chengdu	CHN_Sichuan.Chengdu.562940_CSWD.epw
	Beijing	CHN_Beijing.Beijing.545110_IWEC.epw
	Taipei	TWN_Taipei.466960_IWEC.epw
	Tokyo	JPN_Tokyo.Hyakuri.477150_IWEC.epw
	Sydney	AUS_NSW.Sydney.947670_IWEC.epw

References

(1) Chen, M.; Pang, D.; Mandal, J.; Chen, X.; Yan, H.; He, Y.; Yu, N.; Yang, Y. Designing Mesoporous Photonic Structures for High-Performance Passive Daytime Radiative Cooling. *Nano Lett.* **2021**, *21* (3), 1412-1418. DOI: 10.1021/acs.nanolett.0c04241

(2) Tsai, M.-T.; Chang, S.-W.; Chen, Y.-J.; Chen, H.-L.; Lan, P.-H.; Chen, D.-c.; Ko, F.-H.; Lo, Y.-C.; Wang, H.-C.; Wan, D. Scalable, Flame-Resistant, Superhydrophobic Ceramic Metafibers for Sustainable All-Day Radiative Cooling. *Nano Today* **2023,** *48*, 101745. DOI: 10.1016/j.nantod.2022.101745

(3) Pyun, K. R.; Jeong, S.; Yoo, M. J.; Choi, S. H.; Baik, G.; Lee, M.; Song, J.; Ko, S. H. Tunable Radiative Cooling by Mechanochromic Electrospun Micro-Nanofiber Matrix. *Small* **2024**, *20* (20), 2308572. DOI: 10.1002/smll.202308572

(4) Xin, Y.; Wang, Q.; Fu, C.; Du, S.; Hou, L.; Wei, X.; Wang, H.; Wang, X. Alumina Fiber Membrane Prepared by Electrospinning Technology for Passive Daytime Radiative Cooling. Adv. Funct. Mater. 2025, 35 (3), 2413813. DOI: 10.1002/adfm.202413813

(5) Tian, Y.; Shao, H.; Liu, X.; Chen, F.; Li, Y.; Tang, C.; Zheng, Y. Superhydrophobic and Recyclable Cellulose-Fiber-Based Composites for High-Efficiency Passive Radiative Cooling. *ACS Appl. Mater. Interfaces* **2021**, *13* (19), 22521-22530. DOI: 10.1021/acsami.1c04046

(6) Li, Y.; Ren, M.; Lv, P.; Liu, Y.; Shao, H.; Wang, C.; Tang, C.; Zhou, Y.; Shuai, M. A Robust and Flexible Bulk Superhydrophobic Material from Silicone Rubber/Silica Gel Prepared by Thiol–Ene Photopolymerization. *J. Mater. Chem. A* **2019**, *7* (12), 7242-7255. DOI: 10.1039/c8ta11111a

(7) Tsai, B. K.; Cooksey, C. C.; Allen, D. W.; White, C. C.; Byrd, E.; Jacobs, D. Exposure Study on UV-Induced Degradation of PTFE and Ceramic Optical Diffusers. *Appl. Opt.* **2019**, *58* (5), 1215-1222. DOI: 10.1364/AO.58.001215

(8) Chang, S.-W.; Chen, Y.-J.; Wan, D.; Chen, H.-L. Black and White: A Bifunctional Optical Standard for Near-Perfect White Light Diffuse Reflection and Blackbody Radiation. *Optica* **2024**, *11* (9), 1303-1312. DOI: 10.1364/OPTICA.529111

(9) Wang, X.; Liu, X.; Li, Z.; Zhang, H.; Yang, Z.; Zhou, H.; Fan, T. Scalable Flexible Hybrid Membranes with Photonic Structures for Daytime Radiative Cooling. *Adv. Funct. Mater.* **2020**, *30* (5), 1907562. DOI: 10.1002/adfm.201907562

(10) Fei, J.; Han, D.; Ge, J.; Wang, X.; Koh, S. W.; Gao, S.; Sun, Z.; Wan, M. P.; Ng, B. F.; Cai, L; Li, H. Switchable Surface Coating for Bifunctional Passive Radiative Cooling and Solar Heating. *Adv. Funct. Mater.* **2022**, *32* (27), 2203582. DOI: 10.1002/adfm.202203582

(11) Zhou, K.; Li, W.; Patel, B. B.; Tao, R.; Chang, Y.; Fan, S.; Diao, Y.; Cai, L. Three-Dimensional Printable Nanoporous Polymer Matrix Composites for Daytime Radiative Cooling. *Nano Lett.* **2021**, *21* (3), 1493-1499. DOI: 10.1021/acs.nanolett.0c04810

(12) Liu, B.; Wang, Z.; Zheng, Y.; Zeng, L.; Liu, J.; Fan, T.; Zhou, X. Suppressing Reflectance in Reststrahlen Bands of $Cu_{0.64}Cr_{1.51}Mn_{(0.85-x)}Co_xO_4$ to Achieve Broadband High Emissivity via Phonon Vibration Modes Coupling. *Mater. Today Phys.* **2025**, *51*, 101649. DOI: 10.1016/j.mtphys.2025.101649

(13) Yao, P.; Chen, Z.; Liu, T.; Liao, X.; Yang, Z.; Li, J.; Jiang, Y.; Xu, N.; Li, W.; Zhu, B.; Zhu, J.

Spider-Silk-Inspired Nanocomposite Polymers for Durable Daytime Radiative Cooling. *Adv. Mater.* **2022,** *34* (51), 2208236. DOI: 10.1002/adma.202208236
(14) Song, J.; Zhang, W.; Sun, Z.; Pan, M.; Tian, F.; Li, X.; Ye, M.; Deng, X. Durable Radiative Cooling Against Environmental Aging. *Nat. Commun.* **2022,** *13* (1), 4805. DOI: 10.1038/s41467-022-32409-7
(15) Yang, X.; Geng, J.; Tan, X.; Liu, M.; Yao, S.; Tu, Y.; Li, S.; Qiao, Y.; Qi, G.; Xu, R.; Nie, S. A Flexible PDMS@ ZrO_2 Film for Highly Efficient Passive Radiative Cooling. *Inorg. Chem. Commun.* **2023,** *151*, 110586. DOI: 10.1016/j.inoche.2023.110586
(16) Feng, S.; Yao, L.; Feng, M.; Cai, H.; He, X.; Bu, X.; Huang, Y.; Zhou, Y.; He, M. Superamphiphobic Interface-Enhanced Inorganic Coating for Multi-Environment Tolerant and Zero-Energy Building Radiative Cooling. *Chem. Eng. J.* **2023,** *475*, 146191. DOI: 10.1016/j.cej.2023.146191
(17) Zhao, X.; Li, T.; Xie, H.; Liu, H.; Wang, L.; Qu, Y.; Li, S. C.; Liu, S.; Brozena, A. H.; Yu, Z.; Srebric, J.; Hu, L. A Solution-Processed Radiative Cooling Glass. *Science* **2023,** *382* (6671), 684-691. DOI: 10.1126/science.adi2224
(18) Li, X.; Pattelli, L.; Ding, Z.; Chen, M.; Zhao, T.; Li, Y.; Xu, H.; Pan, L.; Zhao, J. A Novel BST@TPU Membrane with Superior UV Durability for Highly Efficient Daytime Radiative Cooling. *Adv. Funct. Mater.* **2024**, *34*(23), 2315315. DOI: 10.1002/adfm.202315315
(19) Wu, X.-E.; Wang, Y.; Liang, X.; Zhang, Y.; Bi, P.; Zhang, M.; Li, S.; Liang, H.; Wang, S.; Wang, H.; Lu, H.; Zhang, Y. Durable Radiative Cooling Multilayer Silk Textile with Excellent Comprehensive Performance. *Adv. Funct. Mater.* **2024,** *34* (11), 2313539. DOI: 10.1002/adfm.202313539
